# Evolutionary rate patterns of genes involved in the *Drosophila* Toll and Imd signaling pathway

**DOI:** 10.1186/1471-2148-13-245

**Published:** 2013-11-08

**Authors:** Ming Han, Sheng Qin, Xiaojun Song, Yafang Li, Ping Jin, Liming Chen, Fei Ma

**Affiliations:** 1 Laboratory for Comparative Genomics and Bioinformatics & Jiangsu Key Laboratory for Biodiversity and Biotechnology, College of Life Science, Nanjing Normal University, Nanjing 210023, P. R China; 2 The Key Laboratory of Developmental Genes and Human Disease, Ministry of Education, Institute of Life Science, Southeast University, Nanjing 210009, P. R China

**Keywords:** *Drosophila*, Toll and Imd signaling pathway, Molecular evolution, Selective constraint, microRNA

## Abstract

**Background:**

To survive in a hostile environment, insects have evolved an innate immune system to defend against infection. Studies have shown that natural selection may drive the evolution of immune system-related proteins. Yet, how network architecture influences protein sequence evolution remains unclear. Here, we analyzed the molecular evolutionary patterns of genes in the Toll and Imd innate immune signaling pathways across six *Drosophila* genomes within the context of a functional network.

**Results:**

Based on published literature, we identified 50 genes that are directly involved in the *Drosophila* Toll and Imd signaling pathways. Of those genes, only two (*Sphinx1* and *Dnr1*) exhibited signals of positive selection. There existed a negative correlation between the strength of purifying selection and gene position within the pathway; the downstream genes were more conserved, indicating that they were subjected to stronger evolutionary constraints. Interestingly, there was also a significantly negative correlation between the rate of protein evolution and the number of regulatory microRNAs, implying that genes regulated by more miRNAs experience stronger functional constraints and therefore evolve more slowly.

**Conclusion:**

Taken together, our results suggested that both network architecture and miRNA regulation affect protein sequence evolution. These findings improve our understanding of the evolutionary patterns of genes involved in *Drosophila* innate immune pathways.

## Background

Over the past few years, molecular evolution within a network architecture has been of great interest in population genetics [1-7]. Studies on major cellular pathways have demonstrated that network topology constrains the evolutionary pattern of genes. Many reports have demonstrated the existence of a positive correlation between gene pathway position and nucleotide substitution rate. For example, in the melanin synthesis pathway of silkworms [8], the anthocyanin biosynthetic pathway in plants [9-11], and the *Drosophila* Ras signal transduction pathway [12] the upstream genes are subjected to stronger evolutionary constraints. In contrast, an opposite effect was observed in other pathways, such as the animal Toll-like receptor (TLR) [5] and the yeast HOG [13] signaling pathways. Moreover, in the *Caenorhabditis* insulin/TOR signaling transduction pathway, the pattern of selective constraints is driven by expression level [1], whereas in the N-glycosylation metabolic pathways across primates, connectivity of each gene drives the strength of purifying selection [2].

Multiple factors may affect the evolution of genes within networks and pathways, including the gene position, gene expression level [14-16], protein length [17], codon bias [7,18], connectivity [19,20], the number of regulatory microRNAs (miRNAs) that target a gene [21], and the length of its 3′-untranslated region (3′-UTR) [22,23]. Furthermore, studies have demonstrated that miRNAs participate in the regulation of innate immunity and inflammatory responses [24,25]. Thus, miRNA regulation should be analyzed from an evolutionary perspective to improve our understanding of the impact of miRNAs on protein evolution.

**Figure 1:**
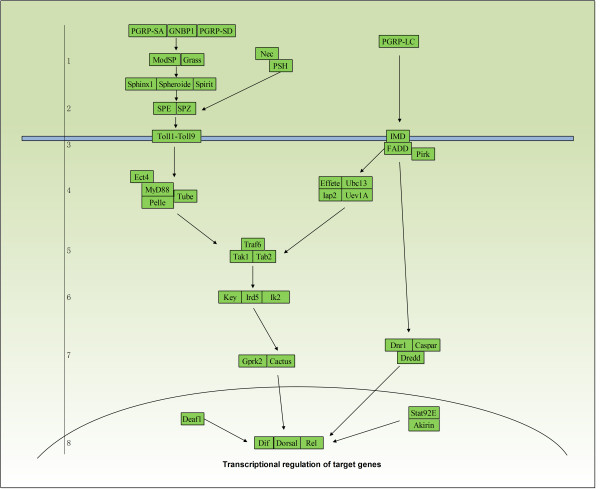
**The *Drosophila *** **Toll and Imd pathways.**

The nuclear factor κB (NF-κB) pathway plays a central role in innate immunity by which invertebrates defend against pathogens [26,27]. *Drosophila* possess two pathways (Toll and Imd innate immune response system; Figure 1) to activate NF-κB transcription factors [27,28]. The Toll pathway is responsible for defense against Gram-positive bacteria or fungi when the cleaved ligand Spatzle binds to the Toll receptor, eventually leading to the activation of the NF-κB family members Dorsal and Dorsal-related immunity factor (Dif). This pathway also participates both in developmental processes [29-31] and immunity [32,33]. In contrast, the Imd pathway controls resistance to Gram-negative bacterial infections [32,34]. The JAK-STAT pathway also correlates with the *Drosophila* innate immune response, but unlike the Toll and Imd pathways, it remains poorly understood, and only four genes (*UPD, DOME, JAK, STAT*) have been reported within this pathway [34-36]. Studies have shown that natural selection may drive the evolution of immune system proteins [37-39]. Yet, the mechanism by which network architecture influences protein evolution within innate immune systems remains unclear. The complete genome sequences of *Drosophila* species and our current knowledge of the innate immune pathways in *D. melanogaster* together offer opportunity for a fine-scale evolutionary analysis of the *Drosophila* Toll and Imd pathways within the context of a network framework.

By surveying recent papers, we identified 50 immune-related genes that are directly involved in the *Drosophila* Toll and Imd signaling pathways, by transferring signals from receptor to transcription factor. We further investigated the evolutionary mechanism of Toll and Imd pathway genes across *Drosophila* species to understand 1) whether there exists a correlation between the strength of purifying selection and gene pathway position within the Toll and Imd innate immune signaling pathways; and 2) which of the topological parameters that characterize network evolution contributes most to the observed selective patterns.

## Results

### Analysis of protein sequence evolution

Variation in selective constraints across immunity pathways was assessed with the help of the program PAML [40] and a comparison of alternative evolutionary models. For all 50 immune-related genes that we identified from the literature, the M0 (one *dN/dS* ratio) model calculated a single nonsynonymous/synonymous substitution rate (*ω* = *dN/dS*) ratio for all branches using a MUSCLE alignment [41]. According to our results, the *Drosophila* immune genes have undergone strong functional constraints, with *ω* values ranging from 0.0001 (*eff*) to 0.4290 (*Spz*) (Additional file 1: Table S1). Additionally, by comparing the M1a (nearly neutral) and M2a (positive selection) models, the M7 (beta) and M8 (beta & *ω*) models, and also the M8 and M8a (beta & *ω*_*s*_ = 1) models, positive selection was observed for *Spz, Mstprox, Tl, Toll-4, cact, Dif, Sphinx1, Ect4,* and *Dnr1* (Additional file 1: Table S2). However, after the false discovery rate test (*q* = 0.05), only five genes (*Dnr1*, *Sphinx1*, *Dif*, *Cact* and *Ect4*) remained significantly positively selected (Additional file 1: Table S2). To improve the reliability of our analysis, we further used a PRANK [42,43] alignment and detected positive selection for *Dnr1*, *Sphinx1*, *Mst* and *Tl* after the FDR test (*q* = 0.05) (Additional file 1: Table S3). The PAML results based on two different alignments (MUSCLE and PRANK) were only consistent for two genes, *Sphinx1* and *Dnr1*. Therefore, these two genes seemed to contain a rather robust signal of positive selection, strongly suggesting that they were indeed subjected to positive selective constraints.

### The strength of purifying selection within Drosophila Toll and Imd signaling pathways

**Figure 2:**
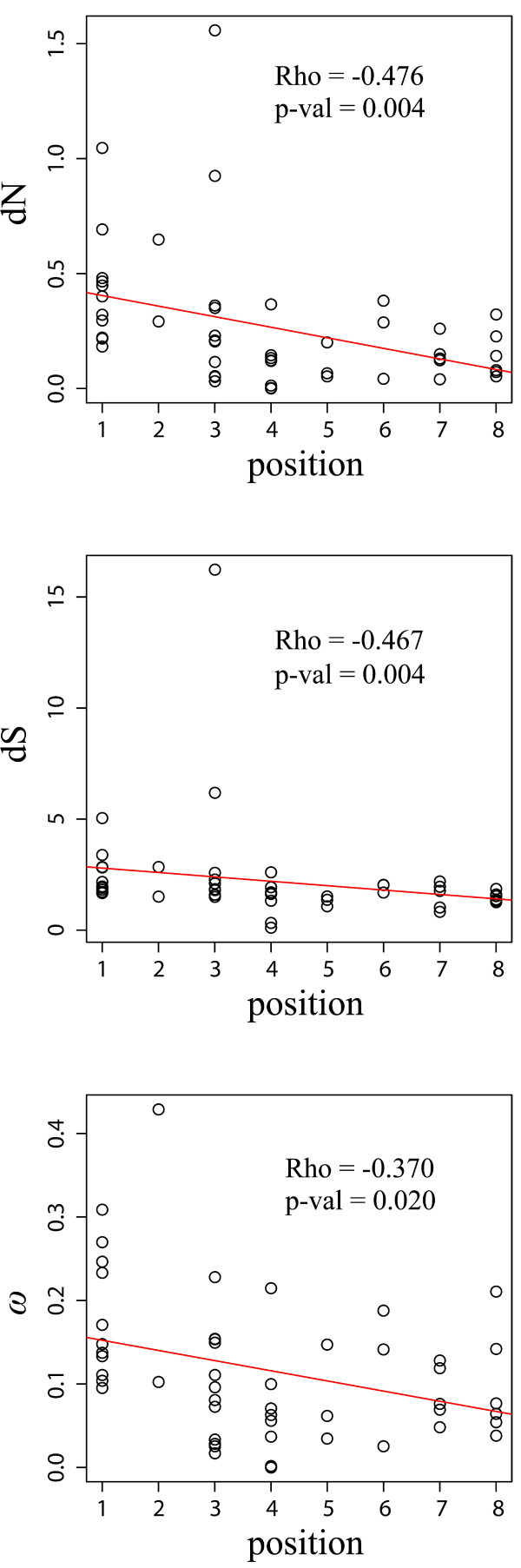
**Nonsynonymous (*dN*) and synonymous (*dS*) substitution rates and their ratio (*ω = dN*/*dS*) as functions of pathway position.** The significant negative correlation between *ω* and pathway position indicated that downstream genes were subjected to stronger purifying selection.

Correlations between evolutionary parameters (*dS*, *dN*, and *ω*) and topological factors (i.e., gene position within the pathway, gene expression level, protein length, codon usage bias, connectivity of individual genes, the number of regulatory miRNAs, the length of 3′-UTR) were estimated applying Spearman’s rank correlation coefficients (*ρ*). For different factors that may affect protein sequence evolution, a significant negative correlation between *ω* values and gene position was observed (*ρ* = −0.370 [−0.529, −0.114], *P* = 0.020 after FDR correction; Figure 2 and Additional file 1: Table S4), indicating that the downstream genes were more conserved and therefore underwent stronger purifying selection. In addition, *dN* and *dS* values were significantly correlated with gene position (*dN*: *ρ* = −0.476 [−0.649, −0.243], *P* = 0.004 after FDR correction; *dS*: *ρ* = −0.467 [−0.645, −0.237], *P* = 0.004 after FDR correction; Figure 2 and Additional file 1: Table S4).

Given that selective constraints can be affected by various topological factors, the relationship between different variables was further analyzed. According to our results, connectivity was significantly correlated with the expression level of immune genes after infection by bacterial (Exp1) or fungal (Exp2) (Exp1: *ρ* = 0.450 [0.197, 0.671], *P* = 0.005 after FDR correction; Exp2: *ρ* = 0.565 [0.329, 0.737], *P* < 0.001 after FDR correction; Additional file 1: Table S4). When we estimated the expression levels after infection at each separate time point, similar results were observed (Additional file 1: Table S5). The highly significant correlation between connectivity and gene expression level indicated that the more connected a protein was, the higher gene expression level it had.

**Figure 3:**
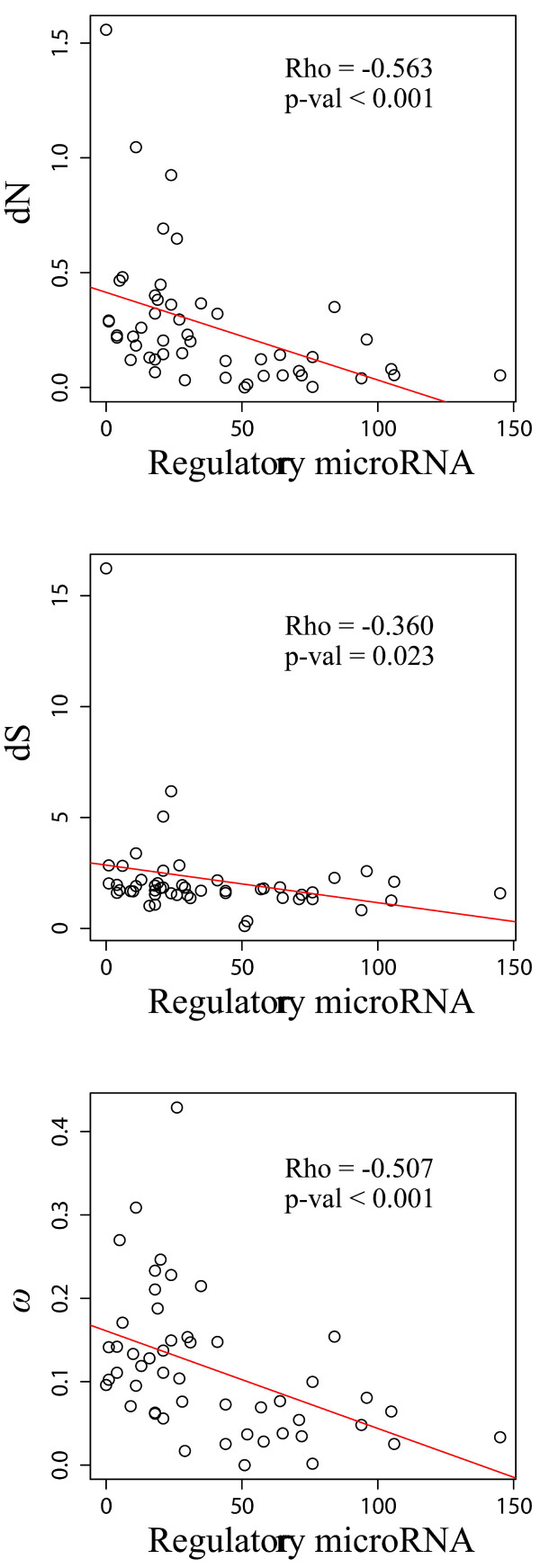
**Nonsynonymous (*dN *) and synonymous (*dS *) substitution rates and their ratio (*ω = dN*/*dS*) as functions of the number of regulatory microRNAs.** The significant negative correlation between *ω* and the number of regulatory miRNAs indicated that genes regulated by more miRNAs were more conserved and therefore evolved more slowly.

We also observed that the number of regulatory miRNAs (*N*_*miR*_, predicted using Miranda 3.3 [44] with default parameters) and the length of the 3′-UTR (*L*_*3′UTR*_) both significantly correlated with *dN*, *dS*, and *ω* (Figure 3 and Table 1). To test the robustness of our predicted miRNA targets, we set the Miranda score to a higher level of 150.0, 160.0, and the correlation remained significant (Additional file 1: Table S4). These results confirmed that genes regulated by more miRNAs were likely to undergo stronger functional constraints and therefore evolve at slower rates [21,45].

**Table 1:** **Spearman’s rank correlation coefficient (*ρ*) of the number of regulatory miRNA and the length of 3′-UTR with *dN*, *dS *** **and *ω***

		***dN***	***dS***	***ω***
N_miR_	*ρ*	−0.563*	−0.360*	−0.507*
	P	<0.001	0.023	<0.001
	lower	−0.702	−0.593	−0.660
	upper	−0.357	−0.111	−0.283
L_3′UTR_	*ρ*	−0.502*	−0.377*	−0.503*
	P	<0.001	0.021	<0.001
	lower	−0.688	−0.599	−0.661
	upper	−0.253	−0.107	−0.259

This table is calculated based on all the genes involved in Toll and Imd pathways;

Lower and upper indicates the confidence intervals of the correlation;

*, *P* < 0.05 after the FDR correction at q = 0.05.

### Multivariate analysis

To investigate whether the observed correlations resulted from direct or indirect influences, two multivariate analysis techniques (partial analysis and path analysis) were performed. As shown in Additional file 1: Table S4, gene pathway position, the number of regulatory miRNAs, and the length of the 3′-UTR are correlated with each other and with the *dN, dS*, and *ω* values. Partial analysis revealed that, when controlling for *N*_*miR*_ and *L*_*3′UTR*_, the correlation between position and *dN* remained significant (*ρ* = −0.318 [−0.506, −0.168], *P* = 0.028) while those between position and *dS* and between position and *ω* disappeared (*dS*: *ρ* = −0.203 [−0.465, −0.076], *P* = 0.167; *ω*: *ρ* = −0.233 [−0.422, 0.010], *P* = 0.112). Similarly, when controlling for *L*_*3′UTR*_, the significant correlations disappeared, except for those between *dN* and position (*ρ* = −0.333 [−0.504, −0.206], *P* = 0.019), between *N*_*miR*_ and *dN* (*ρ* = −0.384 [−0.664, −0.044], *P* = 0.007), and between *N*_*miR*_ and *dS* (*ρ* = −0.361 [−0.701, 0.156], *P* = 0.011), suggesting that the correlations between *N*_*miR*_ and *dN* and between *N*_*miR*_ and *dS* were not mediated by the length of the 3′-UTR. However, when controlling for *N*_*miR*_, only the correlation between *dN* and position remained significant (*ρ* = −0.304 [−0.486, −0.126], *P* = 0.034), while the correlations between *L*_*3′UTR*_ and *dN, dS*, and *ω* were no longer significant (*dN*: *ρ* = 0.072 [−0.372, 0.554], *P* = 0.624; *dS*: *ρ* = 0.270 [−0.373, 0.670], *P* = 0.060; *ω*: *ρ* = −0.248 [−0.439, 0.011], *P* = 0.085), indicating that the correlation between gene evolutionary rate and *L*_*3′UTR*_ was mediated by the number of regulatory miRNAs. Throughout our partial analysis, the significant correlation between pathway position and *dN* consequently indicated that gene position within the pathway is an important parameter that influences protein sequence evolution within a network framework.

**Figure 4:**
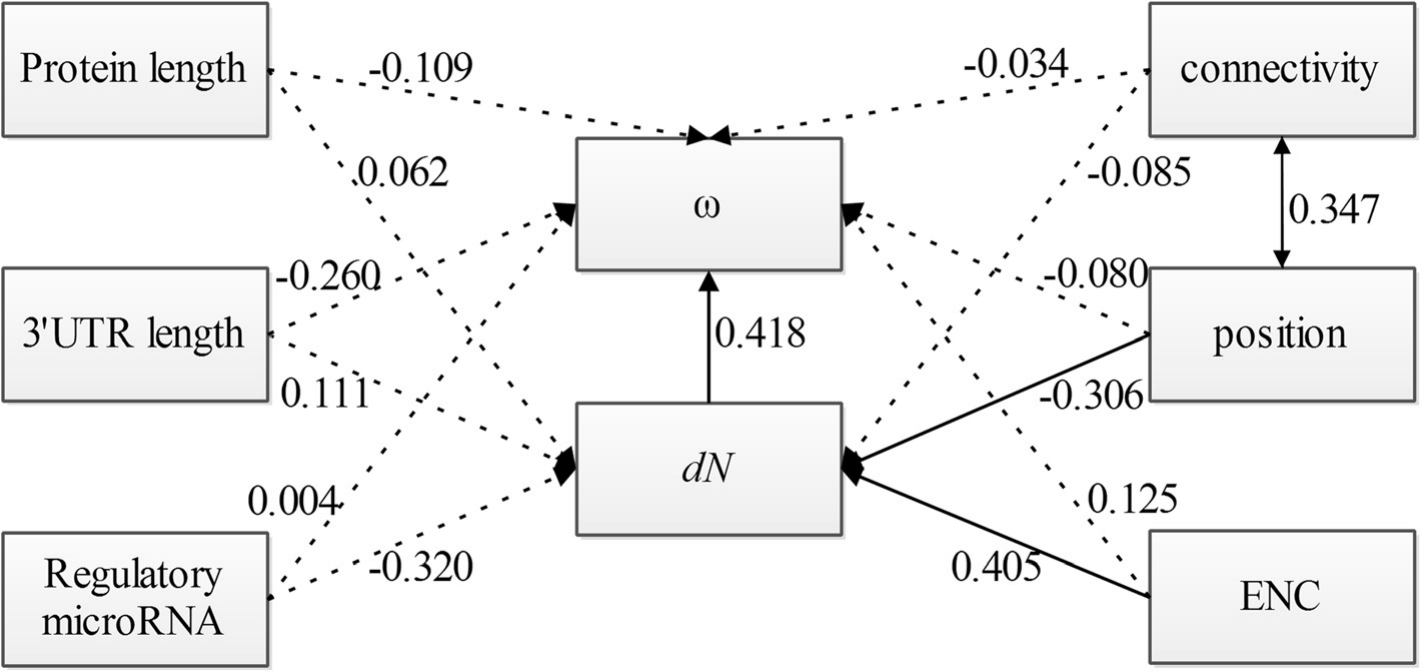
**Path analysis of the relationships among codon bias (ENC), gene position, nonsynonymous substitution rate (*dN*), *dN*/*dS *ratio (*ω*), protein length, PPI, the number of regulatory miRNAs, and the length of the 3′-UTR.** Continuous and broken lines represent significant and nonsignificant relationships, respectively. Double-headed and single-headed arrows indicate the correlations and causal models assumed in the path analysis, respectively. Numbers on the arrows represent the standardized path coefficients (*β*). *dN* and *ω* were considered endogenous variables, while the other variables were considered exogenous.

Through path analysis, the regression coefficients can be decomposed into direct and indirect correlations under the user-defined causal model. We therefore applied path analysis to investigate which contributor primarily constrained protein evolution within the network framework. During our path analysis (Figure 4), *dN* and *ω* were considered endogenous variables while the other variables were considered exogenous. Consistent with the results of partial correlation analysis, we observed that *dN* was significantly affected by position (*β* = −0.306, *P* = 0.020) while *ω* was only affected by *dN* (*β* = 0.418, *P* = 0.008) even after removing the influence of other topological features. In addition, *dN* was significantly correlated with ENC (*β* = 0.405, *P* = 0.002). Thus, Figure 4 demonstrates that pathway position and ENC can affect *ω* by influencing *dN*. Similar results were observed when ENC was considered as an endogenous variable in addition to *dN* and *ω*. Together, these results suggest that among the parameters that describe the network, pathway position is a major contributor to the tendency of the selective constraints.

## Discussion

### Positive selection acting on Toll and Imd pathway genes

There were 40 genes in common between the study by Sackton et al. on the dynamic evolution of the *Drosophila* innate immune system [37] and ours. Sackton and his colleagues detected signals of positive selection for *Dnr1*, *ModSP*, *PGRP-LC*, and *Nec*, whereas we detected signals of positive selection for *Dnr1*, *Sphinx1*, *Dif*, *Cact*, and *Ect4*. The discrepancy between these studies might result from the use of different aligners (Sackton et al.: T-Coffee, us: MUSCLE). To explore the hypothesis, we performed the similarity analysis again using PRANK. Our results showed positive selection for *Dnr1*, *Sphinx1*, *Mst*, and *Tl* after the FDR test (*q* = 0.05). Only two of the genes, *Sphinx1* and *Dnr1*, were consistently identified in at least two of the three analyses based on different alignments. Therefore, these two genes seemed to exhibit a rather robust signal of positive selection, strongly indicating that they have indeed undergone such selective constraints. Overall, the discrepancy between different alignments confirmed that the choice of alignment algorithm has a strong impact on estimates of positive selection [46]. As described in Markova-Raina et al., not all alignment errors are created equal, and even PRANK, which was reported to outperform other aligners in simulation [46], still has a high false positives rate; therefore, one must compare the results of different alignment programs to reduce the rate of false positives in estimates of positive selection.

For *Sphinx1*, which is located upstream in the innate immune pathway, the positive selection acting on it might make it more functional, better adapted to the changing extracellular environment, and subsequently, gain new functions through adaptive evolution [38,47,48]. Furthermore, a quantitative analysis by Obbard et al. [3] confirmed that adaptive evolution is a major factor driving molecular evolution within the *Drosophila* immune system. While we performed our network-level analysis from a phylogenetic perspective, Obbard et al. focused on population genetic data (for *D. melanogaster* and *D. simulans*) to quantify the effects of natural selection. Despite different data and methods in our study and that of Obbard et al., we both found that the peptidoglycan recognition protein and Gram-negative binding protein showed no signs of positive selection, perhaps because of their roles in binding with highly conserved microbial molecules [49].

### Selective constraints and pathway architecture

Because upstream genes are more exposed to the hostile environment, to defend against pathogens, mutations in these genes are likely to have more pleiotropic effects than those in genes acting downstream. Studies have also demonstrated that immune system genes tend to exhibit higher rates of adaptive evolution, which have been attributed to their coevolution with pathogens [37,39,50]. Indeed, in our analysis, we detected a robust negative correlation between the rate of protein evolution and a gene’s position in the *Drosophila* Toll and Imd signaling pathways, indicating that upstream genes experienced more relaxed selective constraints. A similar distribution of purifying selection has also been observed along the insulin/Tor pathways in vertebrates and *Drosophila*[51,52], the animal TLR signaling pathway [5], the N-glycosylation metabolic pathway in primates [2], and the HOG signal transduction pathway in yeast [13].

Throughout our multivariate analysis, the correlation between gene position and *dN* was significant, and given the results of path analysis, we see that pathway position can influence *ω* values through an effect on *dN*. Because *dN/dS* is the measure of selection/constraint, *dN* is actually the metric of selective pressure; these results overall demonstrated that gene position within the network was an important factor driving the polarity of selective constraints along Toll and Imd pathways. Although in the *Caenorhabditis* insulin/TOR signaling transduction pathway, the pattern of selective constraints was driven by expression level [1], and in the N-glycosylation metabolic pathway in primates, connectivity was the main contributor [2] a study of animal TLR signaling pathway [5] agrees with our results in finding a negative relationship between evolutionary constraint and gene position. Because the Toll and Imd signaling pathways are homologous to the mammalian TLR signaling pathway, which plays a vital role in animal innate immunity [32], the shared evolutionary pattern provides strong supporting evidence for our observations.

### The negative correlation between the number of regulatory miRNAs and protein sequence evolution

In addition to the negative correlation between gene evolutionary rates and pathway position, we observed significant correlations between *N*_*miR*_ and the *dS*, *dN*, and *ω* values, and between *L*_*3′UTR*_ and the *dS*, *dN*, and *ω* values. Given the significant correlation between *N*_*miR*_ and *L*_*3′UTR*_, to determine which was the main factor influencing evolutionary rates (or whether they both had an influence), partial analysis was performed. When controlling for *L*_*3′UTR*_, the correlation between *N*_*miR*_ and *dN* and between *N*_*miR*_ and *dS* remained significant. However, when controlling for *N*_*miR*_, the significant correlation between *L*_*3′UTR*_ and *dN, dS* and *ω* all disappeared, indicating that the correlation between gene evolutionary rates and *L*_*3′UTR*_ was mediated by the number of regulatory miRNAs. One possible explanation is that genes regulated by more miRNAs tend to have more molecular functions in different biological processes [53]. Consequently, these pleiotropic genes require more complex and precise regulation by miRNA [45].

In this study, we estimated different topological parameters that may influence the evolution of immune-related gene within a network framework. Because *dN*, *dS*, and *ω* are subjected to many evolutionary pressures, there might be other topological factors that we did not consider in this study. Notably, to estimate the evolutionary pattern of genes in the *Drosophila* immune pathways Toll and IMD, the expression data we analyzed were gene expression levels after infection rather than constitutive expression. Compared with previous studies, we added two more topological parameters to our analysis (the number of regulatory miRNAs and the length of the 3′-UTR) to improve our understanding of the impact of miRNAs on protein sequence evolution.

## Conclusion

We found a polarity in the strength of purifying selection along the *Drosophila* Toll and Imd pathways, with the downstream genes being more conserved. Of all the immune genes investigated, two (*Sphinx1* and *Dnr1*) exhibited signals of positive selection. Notably, we provided strong evidence to show that gene position within the pathway was an important parameter influencing protein sequence evolution within the *Drosophila* Toll and Imd innate immune response systems. Moreover, the negative correlation between protein sequence evolution and the number of regulatory miRNAs confirmed that genes regulated by more miRNAs are likely to undergo stronger functional constraints, and therefore exhibit slower gene evolutionary rates. Further studies investigating the patterns of molecular evolution within different pathways will undoubtedly improve our understanding of natural selection in pathways and networks.

## Methods

### Data collection

By searching research articles and recent reviews [26,32,34-36,54-57], we compiled a list of immune-related genes involved in the Toll and Imd pathways and depicted their relationships (Figure 1). We first downloaded the protein-coding DNA sequences (CDS) of all immune genes (Additional file 1: Table S6) in the *D. melanogaster* genome from FlyBase [58] and then identified orthologs of these genes in other *Drosophila* species (*D. simulans, D. sechellia, D. yakuba, D. erecta,* and *D. ananassae*) using the Database of Orthologous Groups (OrthoDB, http://cegg.unige.ch/orthodb6) [59]. If one gene had several transcripts, the longest was chosen as the ortholog for later analysis.

We recovered genes that were not annotated in OrthoDB through two BLAST steps. We first used protein sequences from *D. melanogaster* as queries and conducted BlastP searches against the whole genome of interest at NCBI, with a BLAST score >150 and length >50. The resulting sequence was then BLAST screened against the *D. melanogaster* genome. If the resulting protein sequence was the same as the original we obtained from FlyBase, we considered the resulting sequence an ortholog of the *D. melanogaster* one.

Some sequences appeared to have long deletions when aligned to orthologous genes; many such cases were artifacts rather than the true cases of deletion. Such sequences often contain stop codons when aligned, and consequently could not be analyzed in PAML. We recovered these incomplete sequences through several steps, as follows: we first conducted a Blat search on the UCSC genome browser [60], and the resulting sequence was used as a query to be predicted in the GeneWise tool with default parameters [61]. Predictions that did not contain frameshift mutations or internal stop codons were considered to be orthologs to *D. melanogaster* sequences. Alignments of these newly-annotated gene sequences are provided in Additional file 2.

### Multiple sequence alignment and phylogenetic analysis

Multiple sequence alignment of the orthologous CDSs was conducted using MUSCLE [41] with default options. Using the resulting alignments, we reconstructed a *Drosophila* phylogeny with MrBayes [62], applying a mixed amino acid substitution model. Four chains with independent runs of 10,000,000 generations were performed to examine the parameter space. We sampled the trees every 1000 generations. The first 2,500 trees were discarded as burn-in. The analyses gave a well-supported topology that corresponded to the known relationships among *Drosophila* species [63]. This topology was used in subsequent codon-based tests of selection.

### Protein sequence evolution analysis

The strength of selective pressures was examined by calculating *dS*, *dN*, and *ω* using the CODEML program in the PAML4.4 package [40]. Values of *ω* < 1, = 1 and > 1 indicate purifying selection, neutral evolution, and positive selection of the target gene, respectively. To avoid synonymous site saturation, which would prevent us from analyzing the more divergent CDS alignments [63], we limited this analysis to the six species in the *melanogaster* group (*D. melanogaster, D. sechellia, D. simulans, D. ananassae, D. erecta,* and *D. yakuba*). The F3 × 4 codon frequency model [64] was applied throughout our codon-based analyses. We first conducted analyses using different models to evaluate changes in selective pressure: M0 (one ratio), M1a (nearly neutral), M2a (positive selection), M7 (beta), M8 (beta & *ω*), and M8a (beta & *ω*_*s*_ = 1). We further tested whether some codon positions have undergone positive selection using a likelihood ratio test (LRT) [65] to compare models, specifically the M1a and M2a models, the M7 and M8 models, and the M8 and M8a models. To avoid false signals of positive selection, we also conducted a false discovery rate (FDR) test [66] controlling the *q* value at 0.05. We also performed our analyses using alignments obtained from PRANK [42,43], which was reported to outperform other aligners in simulation [46], to explore discrepancies between our results and those of Sackton et al. [37].

### Network framework analysis

Because the evolution of molecules within a network can be affected by multiple topological parameters, including gene position within the pathway, connectivity, protein length (L_pro_), codon bias (ENC), gene expression level (mRNA abundance), the number of regulatory miRNAs (*N*_*miR*_) that target the gene, and the length of its 3′-UTR (*L*_*3′UTR*_), we conducted a bivariate correlation analysis between these variables and the selective constraints parameters (*dS*, *dN*, and *ω*) applying Spearman’s rank correlation coefficients (*ρ*).

All the immune genes identified were directly involved in the Toll and Imd innate immune response signaling pathways, transducing signals from signal receptors (i.e., PGRP-SA, PGRP-LC) to transcription factors (*Dif*, *dl*, *Rel*). We assigned a position to each gene according to its function within the pathway. Paralogous genes were assigned the same position. For instance, the nine Toll-related genes (*Tl, 18w, Mstprox, Toll-4, Tehao, Toll-6, Toll-7, Tollo*, *Toll-9*) were assigned the same position. Imd proteins accept signals from Gram-negative bacteria, whereas Toll receptors receive signals from Gram-positive bacteria and fungi. Both Toll and Imd receptors are located on cell membranes, so we assigned the *Imd* gene the same position as Toll. With signals from the activated Toll receptors, three molecules (MyD88, Tube, Pelle) are recruited to form a heterotrimeric complex, and they are closely connected with each other through two distinct death domains, thus their positions were considered the same as well. During microbe recognition, SPZ is cleaved by the activated enzyme SPE and then transduces signals to membrane surface receptors. Based on their close relationship, we attributed the same position to *SPE* and *SPZ*.

We also analyzed the Toll and Imd pathways separately; we observed similar results (Additional file 1: Table S7, Additional file 1: Table S8), which are not discussed further. The Toll and Imd pathways are both homologous to the mammalian TLR signaling pathway, which plays a vital role in animal innate immunity [32], genes in the Toll or Imd pathway are closely associated with each other (e.g., Traf6 in the Toll pathway regulates IκB kinase /NF-κB kinase, and Tak1 in the IMD pathway takes part in the formation of IκB kinase /NF-κB kinase). Additionally, expression profile analyses showed that both the Toll and IMD pathways can be up-regulated upon infection by the same pathogen [67,68]. Therefore, we preferred to analyze the two pathways as a unit.

### Multivariate analysis

To better characterize the relationships among different topological parameters (gene position within the pathway, gene expression level, protein length, ENC, PPI, the number of regulatory miRNAs, the length of 3′-UTR), we conducted partial analysis and path analysis. Through path analysis, the regression coefficients can be decomposed into direct and indirect correlations under a user-defined causal model. We therefore used path analysis to identify the main contributor constraining protein evolution within the network framework. For path analysis, *dN* and *ω* were considered to be endogenous variables while the other variables were considered exogenous. To correct for the fact that the causal model in path analysis is user defined, the analysis was repeated with codon usage bias treated as an endogenous variable in addition to *dN* and *ω.*

All these analyses were conducted applying PASW statistical software. Codon usage bias values were calculated with DnaSP 5.10.01 [69]. Connectivity data (PPIs) of *D. melanogaster* were obtained from the BioGRID database [70]. Because the expression data of immune related genes upon infection was not available for all the *Drosophila* species, we assumed that the relative expression levels of immune-related genes were conserved across species. Expression data of *D. melanogaster* after bacterial (Exp1) or fungal (Exp2) infection were obtained from De Gregorio et al. [67]. The number of regulatory miRNAs targeting the immune genes were predicted with Miranda 3.3 [44] using default parameters. To test the robustness of our predicted miRNA targets, we improved the Miranda score to 150.0, 160.0 and repeated the prediction. The resulting numbers of regulatory miRNAs were defined as N_150_ and N_160_, respectively.

## Abbreviations

MiRNA: microRNA; 3”-UTR: 3‶-untranslated region; Dif: Dorsal-related immunity factor; FDR: False discovery rate; TLR: Toll-Like Receptor; PPI: Protein protein interaction; ENC: Effective number of codons; CDS: Protein coding DNA sequences.

## Competing interests

The authors declare that they have no competing interests.

## Author’s contributions

FM and MH designed the study and drafted the manuscript together. MH, SQ and XJS performed the research and analyzed the data. YFL, LMC and PJ analyzed the data. All authors have read and approved the final version of the manuscript.

## Supplementary Material

Additional file 1: Table S1 Summary statistics used in the multivariate analysis. **Table S2.** Phylogenetic analysis by maximum likelihood of the innate immune pathway genes (MUSCLE alignment). **Table S3.** Phylogenetic analysis by maximum likelihood of the innate immune pathway genes (PRANK alignment). **Table S4.** Bivariate correlations of different factors that may influence evolution of genes within a network (Toll and Imd pathways). **Table S5.** Bivariate correlations between connectivity and expression level of genes after infection. **Table S6.** Genes involved in the *D. melanogaster* innate immune pathway. **Table S7.** Bivariate correlations of different factors that may influence evolution of genes within a network (Toll pathway). **Table S8.** Bivariate correlations of different factors that may influence evolution of genes within a network (Imd pathway). Click here for file

Additional file 2 **Multiple sequence alignments of the Toll and IMD pathway orthologs across six *Drosophila* species.** Alignments of the protein-coding sequences were performed using PRANK. Stop codons were removed from the ends of the alignments. Dots indicate the same nucleotide and “–”represents a missing nucleotide relative to sequences in the first row. Click here for file
