# Supplementary material for: Evolutionary rate patterns of genes involved in the Drosophila Toll and Imd signaling pathway
Source: BMC Evol Biol. 2013 Nov 8;13:245. doi: 10.1186/1471-2148-13-245 (PMC3826850; doi:10.1186/1471-2148-13-245)
Supplement: Additional file 1: Table S1 — Summary statistics used in the multivariate analysis. Table S2. Phylogenetic analysis by maximum likelihood of the innate immune pathway genes (MUSCLE alignment). Table S3. Phylogenetic analysis by maximum likelihood of the innate immune pathway genes (PRANK alignment). Table S4. Bivariate correlations of different factors that may influence evolution of genes within a network (Toll and Imd pathways). Table S5. Bivariate correlations between connectivity and expression level of genes after infection. Table S6. Genes involved in the D. melanogaster innate immune pathway. Table S7. Bivariate correlations of different factors that may influence evolution of genes within a network (Toll pathway). Table S8. Bivariate correlations of different factors that may influence evolution of genes within a network (Imd pathway). [file 1471-2148-13-245-S1.pdf]

Table S1. Summary statistics used in the multivariate analysis

Gene	Position	Protein length	% analysed	*dN*	*dS*	*dN/dS*	PPI	ENC	Exp1	Exp2	N_{miR}	*N140*	*N150*	L3'UTR
PGRP-SA	1	203	0.665	0.2216	1.6626	0.1333	0	46.15	421	89	10	5	1	76
PGRP-SD	1	186	0.5108	0.466	1.727	0.2698	0	55.99	1154	163	5	1	1	135
GNBP1	1	492	0.998	0.217	1.9624	0.1106	0	53.28	48	40	4	1	0	100
ModSP	1	628	0.6959	0.2955	2.8422	0.104	5	48.53	-	-	27	4	1	246
PGRP-LC	1	520	0.95	0.401	1.7194	0.2332	5	54.61	208	87	18	6	0	728
Spheroide	1	249	0.996	0.3213	2.1778	0.1475	13	38.59	240	333	41	13	1	438
Sphinx1	1	253	0.8972	1.0459	3.3894	0.3086	0	57.02	-	-	11	3	1	153
Spirit	1	393	0.1145	0.4476	1.8181	0.2462	0	46.17	73	18	20	7	3	239
grass	1	377	0.9867	0.1819	1.9113	0.0952	0	42.08	83	61	11	5	1	92
Nec	1	476	0.9097	0.4804	2.8137	0.1707	2	41.27	1103	576	6	3	0	108
PSH	1	394	0.9619	0.6926	5.0448	0.1373	0	48.15	48	45	21	7	1	162
SPE	2	400	0.99	0.2911	2.8445	0.1024	23	46.6	472	323	1	1	0	33
SPZ	2	326	0.8067	0.6479	1.5101	0.429	2	50.72	415	182	26	9	0	384
Tl	3	1117	0.5819	0.3497	2.2717	0.154	0	49.65	936	490	84	31	6	1256
18w	3	1385	0.5798	0.0504	1.7931	0.0281	1	39.72	143	195	58	22	5	890
MstProx	3	965	0.4093	0.9239	6.1825	0.1494	0	59.05	5	6	24	8	2	364
Toll-4	3	1125	0.4062	1.5577	16.224	0.096	0	58.59	3	3	0	0	0	1500
Tehao	3	795	0.9824	0.2087	2.5894	0.0806	0	43.36	2	1	96	38	8	69
Toll-6	3	1514	0.8355	0.0314	1.85	0.017	1	42.03	3	3	29	8	1	2548
Toll-7	3	1446	0.9882	0.0535	2.1003	0.0255	1	48.16	3	3	106	43	7	1728

Tollo	3	1346	0.9866	0.0529	1.5914	0.0332	2	45.18	79	76	145	56	11	2671
Toll-9	3	900	0.9567	0.2044	1.8444	0.1108	0	52.4	4	4	21	11	0	197
imd	3	273	0.9853	0.1157	1.5971	0.0725	19	47.66	52	35	44	18	1	376
BG4	3	239	0.9791	0.3606	1.5817	0.228	6	49.76	65	62	24	7	0	335
pirk	3	197	0.9898	0.2299	1.4972	0.1536	3	44.8	246	111	30	10	1	357
Ect4	4	1730	0.115	0.1322	1.3254	0.0998	5	54.1	-	-	76	20	4	1395
Myd88	4	537	0.9721	0.1189	1.6815	0.0707	1	43.27	118	123	9	2	1	1071
Tube	4	462	0.8983	0.3658	1.7049	0.2146	4	54.3	155	180	35	17	3	429
pll	4	501	0.7365	0.1453	2.6068	0.0557	0	47.76	170	92	21	10	2	200
effete	4	147	1	0	0.1002	0.0001	18	55.68	961	845	51	22	5	1081
Iap2	4	498	0.996	0.1215	1.9385	0.0627	12	48	455	313	18	5	1	462
ubc13	4	151	1	0.0027	1.629	0.0017	2	39.83	384	523	76	29	6	1189
Uev1A	4	145	1	0.0122	0.3303	0.037	5	53.62	1040	860	52	21	6	991
Traf6	5	475	0.1832	0.0526	1.5231	0.0346	24	44.05	194	166	72	27	6	1235
Tab2	5	831	0.9495	0.2003	1.3613	0.1471	35	51.21	222	182	31	10	3	391
Tak1	5	678	0.6652	0.0663	1.0771	0.0616	2	43.23	214	175	18	6	0	393
ik2	6	720	0.9861	0.0428	1.6883	0.0254	3	53.58	36	25	44	15	4	208
ird5	6	717	0.4603	0.382	2.0341	0.1878	6	57.76	122	110	19	7	1	233
key	6	389	0.9666	0.2875	2.0313	0.1415	5	56.91	334	180	1	0	0	41
cact	7	500	0.91	0.1305	1.0198	0.1279	30	49.46	3580	1398	16	8	2	748
Gprk2	7	714	0.9944	0.0394	0.8186	0.0481	1	44.05	70	71	94	27	7	1407
caspar	7	695	0.9698	0.1485	1.9462	0.0763	3	52.37	289	290	28	12	5	348
dnr1	7	696	0.921	0.1227	1.7675	0.0694	0	39.15	97	97	57	18	3	908

Dredd	7	494	0.3704	0.2602	2.1902	0.1188	6	49.73	102	96	13	7	0	772
Dif	8	987	0.5755	0.322	1.5299	0.2105	0	50.76	19	17	18	7	3	778
dl	8	999	0.6537	0.0804	1.2504	0.0643	51	50.64	362	189	105	38	11	1143
Rel	8	971	0.9722	0.2273	1.6036	0.1418	3	49.51	2270	454	4	2	0	41
akirin	8	201	1	0.0524	1.3787	0.038	25	40.38	937	996	65	23	4	1120
Deaf1	8	576	0.974	0.0718	1.3196	0.0544	6	45.93	72	71	71	29	6	1496
Stat92E	8	818	0.8998	0.142	1.8534	0.0766	8	48.67	573	615	64	20	3	1438

Exp1, Exp2 represent the average expression level of genes after infection by bacteria or fungi, respectively;
Expression level is missing for *ModSP*, *Sphinx1* and *Ect4*.
N_{miR}, N_{150}, N_{160} represent the number of regulatory miRNAs predicted by Miranda at a score of default (140.0), 150.0, 160.0, respectively.

Table S2. Phylogenetic analysis by maximum likelihood of the innate immune pathway genes (MUSCLE alignment)

Gene	l_{M1a}	l_{M2a}	l_{M7}	l_{M8}	l_{M8a}	$2(l_{M2a}–l_{M1a})$	$2(l_{M8}–l_{M7})$	$2(l_{M8a}–l_{M8})$
spz	-2849.26	-2847.03	-2850.41	-2847.01	-2849.22	4.46*	6.81*	4.42*
18 wheeler	-8857.30	-8857.30	-8854.54	-8854.54	-8854.95	0.00	0.00	0.82
MstProx	-1763.92	-1763.03	-1764.89	-1761.03	-1762.27	1.77	7.72*	2.48
Tehao	-6703.66	-6703.66	-6691.66	-6691.66	-6691.64	0.00	0.00	0.05
Tl	-6401.23	-6400.92	-6396.56	-6393.22	-6394.42	0.60	6.68*	2.41
Toll-4	-5992.69	-5992.69	-5989.86	-5986.42	-5986.37	0.00	6.87*	0.10
Toll-6	-8266.21	-8266.21	-8263.83	-8263.08	-8263.44	0.00	1.52	0.73
Toll-7	-10019.5	-10019.5	-10012.6	-10012.6	-10013.0	0.00	0.01	0.69
Toll-9	-7292.97	-7292.97	-7285.61	-7285.32	-7285.52	0.00	0.58	0.40
Tollo	-8797.32	-8797.32	-8791.15	-8791.15	-8791.14	0.00	0.00	0.01
tub	-4112.57	-4112.57	-4107.86	-4107.81	-4107.86	0.00	0.10	0.10
Myd88	-3904.19	-3904.19	-3896.60	-3896.60	-3896.56	0.00	0.00	0.07
pll	-3021.97	-3021.97	-3012.77	-3012.77	-3012.70	0.00	0.00	0.14
cact	-3228.32	-3221.50	-3228.55	-3220.76	-3228.08	13.63**	15.57**	14.65**
dl	-6346.18	-6346.18	-6303.30	-6303.30	-6303.33	0.00	0.00	0.06
Dif	-7001.17	-6997.01	-7007.45	-6994.25	-7000.14	8.32**	26.40**	11.79**
Relish	-8335.02	-8334.62	-8327.80	-8325.96	-8327.47	0.80	3.69	3.01
PGRP-SA	-1184.78	-1184.78	-1183.51	-1183.41	-1183.46	0.00	0.21	0.12
GNBP1	-4191.44	-4191.44	-4184.25	-4184.25	-4184.21	0.00	0.00	0.08
Gprk2	-4138.58	-4138.50	-4140.49	-4138.51	-4138.60	0.16	3.97	0.18
Traf6	-526.29	-526.29	-525.80	-525.80	-525.80	0.00	0.00	0.00
Ect4	-8727.76	-8663.82	-8717.10	-8650.65	-8716.04	127.88**	132.90**	130.79**
SPE	-3617.84	-3617.84	-3608.15	-3608.15	-3608.14	0.00	0.00	0.03
grass	-3144.87	-3144.87	-3145.03	-3144.06	-3144.08	0.00	1.94	0.04
PGRP-SD	-1028.15	-1028.15	-1027.64	-1027.64	-1027.64	0.00	0.00	0.00
Deaf1	-3836.41	-3836.41	-3833.55	-3833.55	-3833.13	0.00	0.00	0.83
ModSP	-3725.24	-3725.24	-3713.97	-3713.23	-3713.95	0.00	1.48	1.44
Sphinx1	-2785.26	-2782.95	-2788.23	-2780.40	-2781.83	4.62	15.65**	2.85
Spirit	-454.19	-453.16	-453.81	-452.25	-453.81	2.06	3.12	3.11
Spheroide	-2369.00	-2369.00	-2367.03	-2365.65	-2365.85	0.00	2.75	0.39
PSH	-2582.19	-2582.19	-2578.57	-2578.57	-2578.54	0.00	0.00	0.07
Nec	-4409.41	-4409.41	-4390.86	-4390.85	-4390.85	0.00	0.03	0.01
imd	-2063.39	-2063.39	-2064.10	-2063.27	-2063.27	0.00	1.65	0.00
PGRP-LC	-5114.02	-5114.02	-5110.83	-5110.72	-5110.73	0.00	0.22	0.00
BG4	-2281.58	-2281.31	-2281.90	-2281.23	-2281.58	0.54	1.34	0.68

Dredd	-1353.41	-1353.41	-1352.66	-1352.53	-1352.54	0.00	0.25	0.00
Tak1	-2969.27	-2969.27	-2966.79	-2966.79	-2966.74	0.00	0.00	0.10
ik2	-5090.64	-5090.64	-5084.91	-5084.91	-5084.77	0.00	0.00	0.27
ird5	-3334.76	-3334.76	-3332.92	-3331.90	-3332.08	0.00	2.04	0.37
key	-3496.07	-3496.07	-3488.22	-3487.57	-3488.20	0.00	1.30	1.27
Tab2	-6199.75	-6199.75	-6198.16	-6198.16	-6198.15	0.00	0.00	0.02
Iap2	-3820.32	-3820.32	-3823.06	-3820.36	-3820.35	0.00	5.41	0.00
ben	-872.63	-872.63	-873.28	-872.63	-872.63	0.00	1.30	0.00
eff	-644.81	-644.81	-644.81	-644.81	-644.81	0.00	0.00	0.00
Uev1A	-737.85	-737.85	-737.85	-737.85	-737.85	0.00	0.00	0.00
pirk	-1621.67	-1621.67	-1620.91	-1620.78	-1620.89	0.00	0.27	0.22
dnr1	-4785.24	-4782.32	-4790.58	-4780.46	-4784.80	5.84*	20.24**	8.68**
casp	-5270.99	-5270.99	-5268.26	-5267.58	-5267.61	0.00	1.38	0.07
akirin	-1341.44	-1341.44	-1340.65	-1340.65	-1340.65	0.00	0.00	0.01
Stat92E	-5579.77	-5579.77	-5573.92	-5573.92	-5573.94	0.00	0.00	0.03

Positive selection was calculated based on the alignments from MUSCLE.
ℓi, log-likelihood of the observed data under the evolutionary model *i*.
*, P < 0.05; **, statistically significant values at a FDR test of q = 0.05.

Table S3. Phylogenetic analysis by maximum likelihood of the innate immune pathway genes (PRANK alignment)

Gene	l_{M1a}	l_{M2a}	l_{M7}	l_{M8}	l_{M8a}	$2(l_{M2a}–l_{M1a})$	$2(l_{M8}–l_{M7})$	$2(l_{M8a}–l_{M8})$
spz	-2809.95	-2809.95	-2809.88	-2808.07	-2809.61	0.00	3.62	3.07
18 wheeler	-5137.42	-5137.42	-5137.23	-5137.08	-5137.11	0.00	0.30	0.06
MstProx	-1763.20	-1762.28	-1762.53	-1759.07	-1760.86	1.84	6.93**	3.58
Tehao	-6679.05	-6679.05	-6668.58	-6668.58	-6668.57	0.00	0.00	0.01
Tl	-5810.37	-5810.37	-5805.73	-5802.50	-5804.19	0.00	6.46**	3.39
Toll-4	-102.69	-102.69	-103.21	-103.21	-103.20	0.00	0.00	0.03
Toll-6	-7937.74	-7937.74	-7937.02	-7936.26	-7936.50	0.00	1.52	0.48
Toll-7	-9658.04	-9658.04	-9655.73	-9654.96	-9654.94	0.00	1.54	0.03
Toll-9	-7287.45	-7287.45	-7279.80	-7279.46	-7279.72	0.00	0.69	0.53
Tollo	-8654.28	-8654.28	-8647.62	-8647.62	-8647.57	0.00	0.00	0.11
tub	-3497.97	-3497.97	-3494.19	-3494.19	-3494.18	0.00	0.00	0.02
Myd88	-3828.89	-3828.89	-3824.28	-3824.08	-3824.06	0.00	0.41	0.04
pll	-3013.66	-3013.66	-3004.32	-3004.32	-3004.25	0.00	0.00	0.14
cact	-2990.93	-2990.93	-2990.29	-2990.29	-2990.28	0.00	0.00	0.02
dl	-4434.44	-4434.44	-4431.71	-4431.71	-4431.68	0.00	0.00	0.05
Dif	-5595.30	-5595.30	-5595.65	-5594.64	-5594.64	0.00	2.03	0.00
Relish	-8238.97	-8238.97	-8230.54	-8229.27	-8230.41	0.00	2.54	2.27
PGRP-SA	-1150.34	-1150.34	-1147.20	-1147.20	-1147.19	0.00	0.01	0.02
GNBP1	-4184.86	-4184.86	-4176.80	-4176.80	-4176.75	0.00	0.00	0.11
Gprk2	-4097.90	-4097.54	-4100.64	-4097.54	-4097.95	0.73	6.20	0.82
Traf6	-526.29	-526.29	-525.80	-525.80	-525.80	0.00	0.00	0.00
Ect4	-5307.24	-5307.24	-5305.02	-5305.02	-5305.19	0.00	0.00	0.34
SPE	-3615.79	-3615.79	-3606.27	-3606.27	-3606.26	0.00	0.00	0.02
grass	-2674.19	-2674.19	-2669.51	-2669.51	-2669.50	0.00	0.00	0.03
PGRP-SD	-1010.03	-1010.03	-1009.52	-1009.52	-1009.52	0.00	0.00	0.00
Deaf1	-3282.50	-3282.50	-3280.24	-3280.24	-3280.18	0.00	0.00	0.12
ModSP	-3442.07	-3442.07	-3431.17	-3430.33	-3431.14	0.00	1.68	1.63
Sphinx1	-2718.33	-2716.35	-2718.39	-2711.97	-2713.43	3.97*	12.83**	2.92
Spirit	-454.19	-453.16	-453.81	-452.25	-453.81	2.06	3.12	3.11
Spheroide	-2369.00	-2369.00	-2367.03	-2365.65	-2365.85	0.00	2.75	0.39
PSH	-4604.88	-4604.88	-4596.17	-4594.21	-4594.23	0.00	3.93*	0.04
Nec	-3944.47	-3943.02	-3927.51	-3924.85	-3927.50	2.90	5.32*	5.30*
imd	-2063.39	-2063.39	-2064.10	-2063.27	-2063.27	0.00	1.65	0.00
PGRP-LC	-3017.51	-3017.51	-3016.34	-3016.20	-3016.34	0.00	0.27	0.27
BG4	-2271.36	-2271.12	-2271.58	-2270.89	-2271.29	0.47	1.39	0.81
Dredd	-1712.14	-1712.14	-1711.96	-1711.92	-1711.92	0.00	0.08	0.01

Tak1	-2856.10	-2856.10	-2853.96	-2853.96	-2853.92	0.00	0.00	0.08
ik2	-4782.31	-4782.31	-4780.53	-4780.53	-4780.52	0.00	0.00	0.01
ird5	-3292.95	-3292.95	-3289.95	-3289.60	-3289.74	0.00	0.69	0.27
key	-3508.64	-3508.64	-3500.70	-3500.12	-3500.69	0.00	1.16	1.14
Tab2	-5989.97	-5989.97	-5988.37	-5988.37	-5988.35	0.00	0.00	0.05
Iap2	-3741.20	-3741.20	-3741.03	-3740.70	-3740.72	0.00	0.65	0.02
ben	-872.63	-872.63	-873.28	-872.63	-872.63	0.00	1.30	0.00
eff	-644.81	-644.81	-644.81	-644.81	-644.81	0.00	0.00	0.00
Uev1A	-737.85	-737.85	-737.85	-737.85	-737.85	0.00	0.00	0.00
pirk	-1638.50	-1638.50	-1637.81	-1637.54	-1637.71	0.00	0.54	0.33
dnr1	-3733.60	-3730.98	-3736.52	-3728.50	-3733.10	5.23*	16.03**	9.21**
casp	-5191.69	-5191.69	-5189.93	-5188.73	-5188.89	0.00	2.39	0.32
akirin	-1338.36	-1338.36	-1337.57	-1337.57	-1337.56	0.00	0.00	0.01
Stat92E	-5554.94	-5554.94	-5547.74	-5547.74	-5547.72	0.00	0.00	0.04

Positive selection was calculated based on the alignments from PRANK.
ℓi, log-likelihood of the observed data under the evolutionary model *i*.
*, P < 0.05; **, statistically significant values at a FDR test of q = 0.05.

Table S4. Bivariate Correlations of different factors that may influence evolution of genes within network (Toll and Imd pathways)

		position	length	%analysed	dN	dS	ω	PPI	ENC	Exp1	Exp2	N_{miR}	m150	m160	L3utr
position	rho		0.287*	0.014	-0.476**	-0.467**	-0.370**	0.425**	0.026	0.144	0.287	0.291*	0.357**	0.365**	0.391**
	P		0.063	0.924	0.004	0.004	0.020	0.009	0.924	0.394	0.066	0.063	0.020	0.020	0.016
	lower		0.040	-0.267	-0.649	-0.645	-0.592	0.135	-0.238	-0.162	0.010	0.007	0.076	0.114	0.122
	upper		0.509	0.253	-0.243	-0.237	-0.114	0.651	0.300	0.457	0.556	0.542	0.592	0.599	0.630
length	rho	0.287*		-0.375**	-0.103	0.138	-0.196	-0.165	0.066	-0.432**	-0.343*	0.248	0.219	0.237	0.365**
	P	0.063		0.037	0.520	0.408	0.258	0.337	0.651	0.029	0.055	0.195	0.216	0.195	0.037
	lower	0.040		-0.627	-0.401	-0.161	-0.489	-0.413	-0.226	-0.666	-0.601	-0.040	-0.080	-0.044	0.088
	upper	0.509		-0.075	0.230	0.399	0.125	0.116	0.383	-0.126	-0.038	0.527	0.498	0.507	0.619
%analysed	rho	0.014	-0.375**		-0.433**	-0.198	-0.382**	0.228	-0.264	0.108	0.202	0.176	0.189	0.111	-0.032
	P	0.924	0.037		0.019	0.297	0.034	0.297	0.234	0.516	0.297	0.306	0.297	0.516	0.826
	lower	-0.267	-0.627		-0.666	-0.454	-0.628	-0.057	-0.535	-0.181	-0.118	-0.144	-0.128	-0.194	-0.306
	upper	0.253	-0.075		-0.146	0.102	-0.100	0.513	0.050	0.418	0.504	0.467	0.465	0.375	0.239
dN	rho	-0.476**	-0.103	-0.433**		0.568**	0.906**	-0.311**	0.419**	-0.039	-0.186	-0.563**	-0.589**	-0.564**	-0.502**
	P	0.004	0.520	0.019		<0.001	<0.001	0.035	0.003	0.797	0.233	<0.001	<0.001	<0.001	<0.001
	lower	-0.649	-0.401	-0.666		0.326	0.798	-0.534	0.104	-0.344	-0.480	-0.702	-0.744	-0.728	-0.688
	upper	-0.243	0.230	-0.146		0.744	0.960	-0.035	0.665	0.273	0.123	-0.357	-0.381	-0.356	-0.253
dS	rho	-0.467**	0.138	-0.198	0.568**		0.265	-0.420**	0.098	-0.282	-0.287	-0.360**	-0.391**	-0.333**	-0.377**
	P	0.004	0.408	0.297	<0.001		0.070	0.021	0.497	0.070	0.070	0.023	0.021	0.033	0.021
	lower	-0.645	-0.161	-0.454	0.326		0.008	-0.631	-0.183	-0.540	-0.551	-0.593	-0.604	-0.590	-0.599
	upper	-0.237	0.399	0.102	0.744		0.498	-0.131	0.365	0.039	0.027	-0.111	-0.142	-0.071	-0.107
ω	rho	-0.370**	-0.196	-0.382**	0.906**	0.265		-0.173	0.406**	0.120	-0.045	-0.507**	-0.521**	-0.525**	-0.503**

	P	0.020	0.258	0.034	<0.001	0.070		0.306	0.006	0.484	0.766	<0.001	<0.001	<0.001	<0.001
	lower	-0.592	-0.489	-0.628	0.798	0.008		-0.410	0.108	-0.179	-0.346	-0.660	-0.691	-0.703	-0.661
	upper	-0.114	0.125	-0.100	0.960	0.498		0.094	0.641	0.388	0.237	-0.283	-0.285	-0.298	-0.259
PPI	rho	0.425**	-0.165	0.228	-0.311**	-0.420**	-0.173		0.012	0.450**	0.565**	0.212	0.206	0.076	0.225
	P	0.009	0.337	0.297	0.035	0.021	0.306		0.934	0.005	<0.001	0.212	0.212	0.699	0.212
	lower	0.135	-0.413	-0.057	-0.534	-0.631	-0.410		-0.262	0.197	0.329	-0.081	-0.090	-0.191	-0.079
	upper	0.651	0.116	0.513	-0.035	-0.131	0.094		0.292	0.671	0.737	0.464	0.452	0.339	0.495
ENC	rho	0.026	0.066	-0.264	0.419**	0.098	0.406**	0.012		-0.009	-0.106	-0.279	-0.268	-0.182	-0.163
	P	0.924	0.651	0.234	0.003	0.497	0.006	0.934		0.953	0.572	0.180	0.180	0.387	0.387
	lower	-0.238	-0.226	-0.535	0.104	-0.183	0.108	-0.262		-0.320	-0.402	-0.503	-0.500	-0.423	-0.404
	upper	0.300	0.383	0.050	0.665	0.365	0.641	0.292		0.296	0.207	-0.008	0.000	0.069	0.120
Exp1	rho	0.144	-0.432**	0.108	-0.039	-0.282	0.120	0.450**	-0.009		0.920**	-0.132	-0.095	-0.048	-0.088
	P	0.394	0.029	0.516	0.797	0.070	0.484	0.005	0.953		<0.001	0.693	0.693	0.747	0.693
	lower	-0.162	-0.666	-0.181	-0.344	-0.540	-0.179	0.197	-0.320		0.823	-0.435	-0.412	-0.349	-0.388
	upper	0.457	-0.126	0.418	0.273	0.039	0.388	0.671	0.296		0.969	0.201	0.236	0.252	0.260
Exp2	rho	0.287	-0.343*	0.202	-0.186	-0.287	-0.045	0.565**	-0.106	0.920**		0.047	0.085	0.079	0.086
	P	0.066	0.055	0.297	0.233	0.070	0.766	<0.001	0.572	<0.001		0.752	0.752	0.752	0.752
	lower	0.010	-0.601	-0.118	-0.480	-0.551	-0.346	0.329	-0.402	0.823		-0.251	-0.229	-0.224	-0.230
	upper	0.556	-0.038	0.504	0.123	0.027	0.237	0.737	0.207	0.969		0.355	0.381	0.368	0.422
N_{miR}	rho	0.291*	0.248	0.176	-0.563**	-0.360**	-0.507**	0.212	-0.279	-0.132	0.047		0.964**	0.838**	0.619**
	P	0.063	0.195	0.306	<0.001	0.023	<0.001	0.212	0.180	0.693	0.752		<0.001	<0.001	<0.001
	lower	0.007	-0.040	-0.144	-0.702	-0.593	-0.660	-0.081	-0.503	-0.435	-0.251		0.906	0.713	0.324
	upper	0.542	0.527	0.467	-0.357	-0.111	-0.283	0.464	-0.008	0.201	0.355		0.986	0.918	0.847

m150	rho	0.357**	0.219	0.189	-0.589**	-0.391**	-0.521**	0.206	-0.268	-0.095	0.085	0.964**		0.847**	0.603**
	P	0.020	0.216	0.297	<0.001	0.021	<0.001	0.212	0.180	0.693	0.752	<0.001		<0.001	<0.001
	lower	0.076	-0.080	-0.128	-0.744	-0.604	-0.691	-0.090	-0.500	-0.412	-0.229	0.906		0.712	0.318
	upper	0.592	0.498	0.465	-0.381	-0.142	-0.285	0.452	0.000	0.236	0.381	0.986		0.925	0.838
m160	rho	0.365**	0.237	0.111	-0.564**	-0.333**	-0.525**	0.076	-0.182	-0.048	0.079	0.838**	0.847**		0.539**
	P	0.020	0.195	0.516	<0.001	0.033	<0.001	0.699	0.387	0.747	0.752	<0.001	<0.001		<0.001
	lower	0.114	-0.044	-0.194	-0.728	-0.590	-0.703	-0.191	-0.423	-0.349	-0.224	0.713	0.712		0.267
	upper	0.599	0.507	0.375	-0.356	-0.071	-0.298	0.339	0.069	0.252	0.368	0.918	0.925		0.766
L3utr	rho	0.391**	0.365**	-0.032	-0.502**	-0.377**	-0.503**	0.225	-0.163	-0.088	0.086	0.619**	0.603**	0.539**	
	P	0.016	0.037	0.826	<0.001	0.021	<0.001	0.212	0.387	0.693	0.752	<0.001	<0.001	<0.001	
	lower	0.122	0.088	-0.306	-0.688	-0.599	-0.661	-0.079	-0.404	-0.388	-0.230	0.324	0.318	0.267	
	upper	0.630	0.619	0.239	-0.253	-0.107	-0.259	0.495	0.120	0.260	0.422	0.847	0.838	0.766	

This table is calculated based on all the genes involved in Toll and Imd pathways;
Lower and upper indicates the confidence intervals of the correlation;
*, $P < 0.05$ when bivariate correlations of different factors were calculated;
**, $P < 0.05$ after the FDR correction at q = 0.05.

Table S5. Bivariate Correlations between connectivity and expression level of genes after infection

		$Exp\text{-}_{12F}$	$Exp\text{-}_{24F}$	$Exp\text{-}_{48F}$	$Exp\text{-}_{96F}$	$Exp\text{-}_{1.5B}$	$Exp\text{-}_{3B}$	$Exp\text{-}_{6B}$	$Exp\text{-}_{12B}$	$Exp\text{-}_{24B}$	$Exp\text{-}_{48B}$	$Exp\text{-}_{B}$	$Exp\text{-}_{F}$
PPI	rho	0.564*	0.554*	0.542*	0.539*	0.452*	0.432*	0.473*	0.461*	0.436*	0.476*	0.450*	0.565*
	P	<0.001	<0.001	<0.001	<0.001	0.001	0.002	0.001	0.001	0.002	0.001	0.005	<0.001
	lower	0.309	0.304	0.299	0.307	0.192	0.163	0.202	0.177	0.147	0.229	0.197	0.329
	upper	0.741	0.725	0.735	0.723	0.670	0.665	0.691	0.681	0.673	0.683	0.671	0.737

Correlations are calculated between connectivity and expression level of genes after infection by different microbial;
$Exp\text{-}_{12F}$, $Exp\text{-}_{24F}$, $Exp\text{-}_{48F}$ and $Exp\text{-}_{96F}$ represent expression level of genes after fungal natural infection at each separate time point: 12h, 24h, 48h, 96h;
$Exp\text{-}_{1.5B}$, $Exp\text{-}_{3B}$, $Exp\text{-}_{6B}$, $Exp\text{-}_{12B}$, $Exp\text{-}_{24B}$ and $Exp\text{-}_{48B}$ represent expression level of genes after septic infection of a concentrated bacterial culture at each separate time point: 1.5h, 3h, 6h, 12h, 24h, 48h;
$Exp\text{-}_{B}$ represents the average expression level of genes after septic infection of a concentrated bacterial culture of six time points;
$Exp\text{-}_{F}$ represents the average expression level of genes after fungal natural infection of four time points;
Lower and upper indicates the confidence intervals of the correlation;
*, $P < 0.05$ after the FDR correction at q = 0.05;

Table S6. Genes involved in the *D. melanogaster* innate immune pathway

Gene	Accession number	FlyBase ID	Protein	Number of isoforms	Chosen isoform	Protein length	Chromosome	start-Position	end-Position
spz	CG6134	FBgn0003495	spatzle	8	A	326	3R	22,890,712	22,895,792
18 wheeler	CG8896	FBgn0004364	18 wheeler	1	A	1385	2R	15,999,016	16,004,437
MstProx	CG1149	FBgn0015770	MstProx	2	A	965	3R	3,191,661	3,195,027
Tehao	CG7121	FBgn0026760	Tehao	1	A	795	2L	13,435,622	13,439,333
Tl	CG5490	FBgn0262473	Toll	3	C	1117	3R	22,624,765	22,668,125
Toll-4	CG18241	FBgn0032095	Toll-4	1	A	1125	2L	9,084,107	9,089,440
Toll-6	CG7250	FBgn0036494	Toll-6	3	A	1514	3L	15,329,792	15,337,417
Toll-7	CG8595	FBgn0034476	Toll-7	1	A	1446	2R	15,714,410	15,720,473
Toll-9	CG5528	FBgn0036978	Toll-9	3	A	900	3L	20,354,943	20,359,824
Tollo	CG6890	FBgn0029114	Tollo	1	A	1346	3L	15,228,719	15,235,932
tub	CG10520	FBgn0003882	tube	1	C	462	3R	213,464	215,535
Myd88	CG2078	FBgn0033402	dMyd88	2	A	537	2R	5,190,328	5,196,227
pll	CG5974	FBgn0010441	pelle	3	A	501	3R	23,076,853	23,078,904
cact	CG5848	FBgn0000250	cactus	4	A	500	2L	16,313,029	16,326,101
dl	CG6667	FBgn0260632	dorsal	5	C	999	3L	17,436,830	17,450,360
Dif	CG6794	FBgn0011274	Dif	4	C	987	2L	17,413,248	17,439,923
Relish	CG11992	FBgn0014018	Relish	5	C	971	3R	4,869,900	4,873,699
PGRP-SA	CG11709	FBgn0030310	PGRP-SA	1	A	203	X	11,455,562	11,456,813
GNBP1	CG6895	FBgn0040323	GNBP1	1	A	492	3L	18,668,900	18,670,976
Gprk2	CG17998	FBgn0261988	Gprk2	2	A	714	3R	27,230,967	27,283,595

Traf6	CG10961	FBgn0026318	Traf6	1	A	475	X	8,048,632	8,051,399
Ect4	CG43119	FBgn0262579	Ect4	8	I	1730	3L	8,056,974	8,101,936
SPE	CG16705	FBgn0039102	SPE	1	A	400	3R	19,511,977	19,513,869
grass	CG5896	FBgn0039494	grass	2	B	377	3R	22,983,668	22,985,503
PGRP-SD	CG7496	FBgn0035806	PGRP-SD	1	A	186	3L	7,644,280	7,645,000
Deaf1	CG8567	FBgn0013799	Deaf1	5	A	576	3L	19,811,274	19,823,786
ModSP	CG31217	FBgn0051217	modSP	1	A	628	3R	12,478,168	12,481,384
Sphinx1	CG32383	FBgn0052383	sphinx1	1	B	253	3L	7,431,521	7,432,505
Spirit	CG2056	FBgn0030051	spirit	4	B	393	X	8,465,132	8,467,626
Spheroide	CG9675	FBgn0030774	spheroide	1	A	249	X	16,594,479	16,595,978
PSH	CG6367	FBgn0030926	persephone	1	A	394	X	18,378,515	18,380,954
Nec	CG1857	FBgn0002930	necrotic	1	A	476	2R	3,043,982	3,045,746
imd	CG5576	FBgn0013983	imd	1	A	273	2R	14,297,296	14,299,024
PGRP-LC	CG4432	FBgn0035976	PGRP-LC	7	B	520	3L	9,331,910	9,341,436
BG4	CG12297	FBgn0038928	dFADD	1	A	239	3R	17,860,177	17,861,339
Dredd	CG7486	FBgn0020381	Dredd	3	E	494	X	527,553	529,760
Tak1	CG18492	FBgn0026323	dTAK1	1	A	678	X	20,386,932	20,395,953
ik2	CG2615	FBgn0086657	IKK Îµ	2	B	720	2L	20,676,234	20,679,308
ird5	CG4201	FBgn0024222	DmIkkβ	1	B	717	3R	11,872,069	11,875,143
key	CG16910	FBgn0041205	DmIKKγ	2	B	389	2R	20,673,037	20,674,932
Tab2	CG7417	FBgn0086358	Tab2	3	A	831	2R	15,180,034	15,192,013
Iap2	CG8293	FBgn0015247	Iap2	2	A	498	2R	11,819,675	11,822,330
Ubc13	CG18319	FBgn0000173	ubc13	1	A	151	X	13,890,387	13,893,841

eff	CG7425	FBgn0011217	effete	3	A	147	3R	10,558,136	10,567,041
Uev1A	CG10640	FBgn0035601	Uev1A	1	A	145	3L	5,355,984	5,359,325
pirk	CG15678	FBgn0034647	pirk	1	A	197	2R	17,548,472	17,549,749
dnr1	CG12489	FBgn0260866	dnr1	2	B	696	2R	18,450,657	18,480,473
casp	CG8400	FBgn0034068	caspar	2	A	695	2R	11,912,133	11,915,666
akirin	CG8580	FBgn0082598	akirin	6	A	201	3L	7,362,943	7,366,811
Stat92E	CG4257	FBgn0016917	STAT	11	K	818	3R	16,361,045	16,378,033

Table S7. Bivariate Correlations of different factors that may influence evolution of genes within network (Toll pathway)

		position	length	%analysed	dN	dS	ω	PPI	ENC	Exp1	Exp2	N_{miR}	m150	m160	L3utr
position	rho		0.362**	0.033	-0.490**	-0.585**	-0.336*	0.524**	0.092	0.118	0.242	0.292	0.363**	0.374**	0.385**
	P		0.044	0.842	0.007	0.001	0.060	0.004	0.626	0.583	0.201	0.103	0.044	0.044	0.044
	lower		0.011	-0.296	-0.674	-0.747	-0.578	0.216	-0.216	-0.233	-0.090	-0.040	0.050	0.093	0.083
	upper		0.642	0.362	-0.247	-0.359	-0.038	0.762	0.365	0.471	0.557	0.548	0.609	0.618	0.645
length	rho	0.362**		-0.206	-0.335*	-0.070	-0.408**	-0.005	0.119	-0.439**	-0.290	0.413**	0.398**	0.366**	0.536**
	P	0.044		0.276	0.064	0.735	0.029	0.976	0.565	0.029	0.129	0.029	0.029	0.044	0.005
	lower	0.011		-0.538	-0.638	-0.375	-0.692	-0.328	-0.247	-0.689	-0.576	0.083	0.061	0.059	0.206
	upper	0.642		0.130	0.014	0.220	-0.071	0.317	0.456	-0.118	0.072	0.677	0.656	0.631	0.740
%analysed	rho	0.033	-0.206		-0.331*	-0.025	-0.311	0.174	-0.357*	-0.040	0.064	0.073	0.100	-0.015	-0.141
	P	0.842	0.276		0.198	0.928	0.198	0.794	0.198	0.928	0.928	0.928	0.928	0.928	0.860
	lower	-0.296	-0.538		-0.595	-0.343	-0.570	-0.157	-0.625	-0.361	-0.299	-0.271	-0.233	-0.336	-0.484
	upper	0.362	0.130		-0.003	0.325	0.025	0.495	-0.032	0.310	0.408	0.422	0.426	0.339	0.186
dN	rho	-0.490**	-0.335*	-0.331*		0.587**	0.874**	-0.363**	0.505**	0.110	-0.013	-0.520**	-0.536**	-0.501**	-0.456**
	P	0.007	0.064	0.198		<0.001	<0.001	0.029	0.002	0.581	0.940	0.002	0.001	0.002	0.005
	lower	-0.674	-0.638	-0.595		0.307	0.718	-0.575	0.184	-0.245	-0.368	-0.689	-0.715	-0.713	-0.680
	upper	-0.247	0.014	-0.003		0.771	0.951	-0.084	0.753	0.473	0.346	-0.274	-0.294	-0.212	-0.165
dS	rho	-0.585**	-0.070	-0.025	0.587**		0.238	-0.439**	0.175	-0.262	-0.263	-0.308	-0.355*	-0.344*	-0.354*
	P	0.001	0.735	0.928	<0.001		0.163	0.046	0.286	0.158	0.158	0.102	0.073	0.073	0.073
	lower	-0.747	-0.375	-0.343	0.307		-0.067	-0.686	-0.169	-0.570	-0.568	-0.599	-0.625	-0.642	-0.616
	upper	-0.359	0.220	0.325	0.771		0.504	-0.103	0.478	0.109	0.092	0.037	-0.027	0.007	-0.064
ω	rho	-0.336*	-0.408**	-0.311	0.874**	0.238		-0.203	0.485**	0.318	0.189	-0.463**	-0.455**	-0.429**	-0.468**

	P	0.060	0.029	0.198	<0.001	0.163		0.246	0.007	0.078	0.271	0.007	0.007	0.010	0.007
	lower	-0.578	-0.692	-0.570	0.718	-0.067		-0.463	0.200	0.014	-0.135	-0.652	-0.675	-0.661	-0.678
	upper	-0.038	-0.071	0.025	0.951	0.504		0.099	0.731	0.572	0.481	-0.210	-0.167	-0.125	-0.202
PPI	rho	0.524**	-0.005	0.174	-0.363**	-0.439**	-0.203		-0.112	0.489**	0.646**	0.268	0.252	0.172	0.247
	P	0.004	0.976	0.794	0.029	0.046	0.246		0.496	0.009	<0.001	0.181	0.181	0.345	0.181
	lower	0.216	-0.328	-0.157	-0.575	-0.686	-0.463		-0.401	0.176	0.406	-0.069	-0.066	-0.142	-0.069
	upper	0.762	0.317	0.495	-0.084	-0.103	0.099		0.198	0.705	0.804	0.548	0.529	0.450	0.543
ENC	rho	0.092	0.119	-0.357*	0.505**	0.175	0.485**	-0.112		-0.092	-0.189	-0.253	-0.267	-0.181	-0.161
	P	0.626	0.565	0.198	0.002	0.286	0.007	0.496		0.595	0.393	0.359	0.359	0.393	0.393
	lower	-0.216	-0.247	-0.625	0.184	-0.169	0.200	-0.401		-0.397	-0.487	-0.534	-0.538	-0.481	-0.452
	upper	0.365	0.456	-0.032	0.753	0.478	0.731	0.198		0.277	0.160	0.049	0.034	0.133	0.171
Exp1	rho	0.118	-0.439**	-0.040	0.110	-0.262	0.318	0.489**	-0.092		0.921**	-0.209	-0.166	-0.187	-0.209
	P	0.583	0.029	0.928	0.581	0.158	0.078	0.009	0.595		<0.001	0.334	0.334	0.334	0.334
	lower	-0.233	-0.689	-0.361	-0.245	-0.570	0.014	0.176	-0.397		0.777	-0.556	-0.518	-0.518	-0.553
	upper	0.471	-0.118	0.310	0.473	0.109	0.572	0.705	0.277		0.977	0.148	0.190	0.151	0.174
Exp2	rho	0.242	-0.290	0.064	-0.013	-0.263	0.189	0.646**	-0.189	0.921**		-0.037	0.009	-0.075	-0.033
	P	0.201	0.129	0.928	0.940	0.158	0.271	<0.001	0.393	<0.001		0.960	0.960	0.960	0.960
	lower	-0.090	-0.576	-0.299	-0.368	-0.568	-0.135	0.406	-0.487	0.777		-0.391	-0.334	-0.433	-0.397
	upper	0.557	0.072	0.408	0.346	0.092	0.481	0.804	0.160	0.977		0.334	0.352	0.290	0.344
N_{miR}	rho	0.292	0.413**	0.073	-0.520**	-0.308	-0.463**	0.268	-0.253	-0.209	-0.037		0.964**	0.861**	0.625**
	P	0.103	0.029	0.928	0.002	0.102	0.007	0.181	0.359	0.334	0.960		<0.001	<0.001	<0.001
	lower	-0.040	0.083	-0.271	-0.689	-0.599	-0.652	-0.069	-0.534	-0.556	-0.391		0.907	0.704	0.301
	upper	0.548	0.677	0.422	-0.274	0.037	-0.210	0.548	0.049	0.148	0.334		0.988	0.947	0.895

m150	rho	0.363**	0.398**	0.100	-0.536**	-0.355*	-0.455**	0.252	-0.267	-0.166	0.009	0.964**		0.860**	0.597**
	P	0.044	0.029	0.928	0.001	0.073	0.007	0.181	0.359	0.334	0.960	<0.001		<0.001	<0.001
	lower	0.050	0.061	-0.233	-0.715	-0.625	-0.675	-0.066	-0.538	-0.518	-0.334	0.907		0.678	0.269
	upper	0.609	0.656	0.426	-0.294	-0.027	-0.167	0.529	0.034	0.190	0.352	0.988		0.948	0.863
m160	rho	0.374**	0.366**	-0.015	-0.501**	-0.344*	-0.429**	0.172	-0.181	-0.187	-0.075	0.861**	0.860**		0.562**
	P	0.044	0.044	0.928	0.002	0.073	0.010	0.345	0.393	0.334	0.960	<0.001	<0.001		<0.001
	lower	0.093	0.059	-0.336	-0.713	-0.642	-0.661	-0.142	-0.481	-0.518	-0.433	0.704	0.678		0.247
	upper	0.618	0.631	0.339	-0.212	0.007	-0.125	0.450	0.133	0.151	0.290	0.947	0.948		0.817
L3utr	rho	0.385**	0.536**	-0.141	-0.456**	-0.354*	-0.468**	0.247	-0.161	-0.209	-0.033	0.625**	0.597**	0.562**	
	P	0.044	0.005	0.860	0.005	0.073	0.007	0.181	0.393	0.334	0.960	<0.001	<0.001	<0.001	
	lower	0.083	0.206	-0.484	-0.680	-0.616	-0.678	-0.069	-0.452	-0.553	-0.397	0.301	0.269	0.247	
	upper	0.645	0.740	0.186	-0.165	-0.064	-0.202	0.543	0.171	0.174	0.344	0.895	0.863	0.817	

This table is calculated based on all the genes involved in Toll pathway;
Lower and upper indicates the confidence intervals of the correlation;
*, $P < 0.05$ when bivariate correlations of different factors were calculated;
**, $P < 0.05$ after the FDR correction at q = 0.05.

Table S8. Bivariate Correlations of different factors that may influence evolution of genes within network (Imd pathway)

		position	length	%analysed	dN	dS	ω	PPI	ENC	Exp1	Exp2	N_{miR}	m150	m160	L3utr
position	rho		0.610**	-0.343	-0.012	0.023	-0.041	-0.010	-0.134	0.082	0.122	0.121	0.194	0.279	0.295
	P		0.016	0.574	0.963	0.963	0.963	0.963	0.918	0.963	0.918	0.918	0.914	0.574	0.574
	lower		0.278	-0.637	-0.431	-0.355	-0.480	-0.427	-0.488	-0.382	-0.345	-0.291	-0.234	-0.133	-0.128
	upper		0.836	0.049	0.463	0.354	0.444	0.364	0.249	0.473	0.517	0.508	0.575	0.619	0.642
length	rho	0.610**		-0.565**	0.304	0.162	0.318	-0.110	0.125	-0.244	-0.263	-0.061	-0.133	0.027	-0.079
	P	0.016		0.039	0.559	0.800	0.559	0.800	0.800	0.576	0.576	0.843	0.800	0.897	0.843
	lower	0.278		-0.829	-0.137	-0.263	-0.131	-0.519	-0.293	-0.617	-0.620	-0.466	-0.526	-0.403	-0.499
	upper	0.836		-0.154	0.672	0.542	0.683	0.343	0.525	0.209	0.216	0.374	0.318	0.472	0.340
% analysed	rho	-0.343	-0.565**		-0.519*	-0.297	-0.445*	-0.100	-0.120	0.267	0.252	0.266	0.247	0.243	0.034
	P	0.574	0.039		0.086	0.333	0.143	0.699	0.695	0.333	0.333	0.333	0.333	0.333	0.874
	lower	-0.637	-0.829		-0.789	-0.674	-0.758	-0.522	-0.555	-0.151	-0.195	-0.186	-0.190	-0.183	-0.413
	upper	0.049	-0.154		-0.037	0.134	0.049	0.296	0.354	0.618	0.613	0.665	0.634	0.621	0.416
dN	rho	-0.012	0.304	-0.519*		0.565**	0.963**	-0.055	0.388	-0.223	-0.330	-0.673**	-0.709**	-0.709**	-0.540**
	P	0.963	0.559	0.086		0.007	<0.001	0.796	0.079	0.315	0.134	0.001	<0.001	<0.001	0.009
	lower	-0.431	-0.137	-0.789		0.257	0.890	-0.448	-0.089	-0.595	-0.669	-0.809	-0.870	-0.850	-0.762
	upper	0.463	0.672	-0.037		0.798	0.983	0.354	0.778	0.214	0.085	-0.425	-0.446	-0.456	-0.201
dS	rho	0.023	0.162	-0.297	0.565**		0.374	-0.187	0.184	-0.202	-0.221	-0.404*	-0.464*	-0.468*	-0.417*
	P	0.963	0.800	0.333	0.007		0.118	0.379	0.379	0.379	0.379	0.101	0.088	0.088	0.101
	lower	-0.355	-0.263	-0.674	0.257		0.042	-0.521	-0.270	-0.571	-0.578	-0.707	-0.721	-0.748	-0.688
	upper	0.354	0.542	0.134	0.798		0.660	0.247	0.594	0.237	0.216	-0.004	-0.113	-0.125	-0.059
ω	rho	-0.041	0.318	-0.445*	0.963**	0.374		-0.043	0.342	-0.182	-0.295	-0.628**	-0.667**	-0.675**	-0.514**

	P	0.963	0.559	0.143	<0.001	0.118		0.839	0.150	0.440	0.203	0.002	0.001	0.001	0.017
	lower	-0.480	-0.131	-0.758	0.890	0.042		-0.408	-0.102	-0.570	-0.654	-0.773	-0.838	-0.816	-0.764
	upper	0.444	0.683	0.049	0.983	0.660		0.355	0.728	0.251	0.148	-0.389	-0.385	-0.405	-0.140
PPI	rho	-0.010	-0.110	-0.100	-0.055	-0.187	-0.043		0.160	0.336	0.371	0.132	0.206	0.096	0.151
	P	0.963	0.800	0.699	0.796	0.379	0.839		0.617	0.351	0.351	0.617	0.617	0.647	0.617
	lower	-0.427	-0.519	-0.522	-0.448	-0.521	-0.408		-0.331	-0.076	-0.024	-0.339	-0.211	-0.296	-0.217
	upper	0.364	0.343	0.296	0.354	0.247	0.355		0.534	0.654	0.681	0.552	0.563	0.498	0.510
ENC	rho	-0.134	0.125	-0.120	0.388	0.184	0.342	0.160		0.053	-0.030	-0.416*	-0.365	-0.180	-0.461*
	P	0.918	0.800	0.695	0.079	0.379	0.150	0.617		0.885	0.885	0.115	0.146	0.584	0.115
	lower	-0.488	-0.293	-0.555	-0.089	-0.270	-0.102	-0.331		-0.327	-0.418	-0.710	-0.704	-0.554	-0.709
	upper	0.249	0.525	0.354	0.778	0.594	0.728	0.534		0.399	0.349	-0.057	0.049	0.231	-0.100
Exp1	rho	0.082	-0.244	0.267	-0.223	-0.202	-0.182	0.336	0.053		0.966**	-0.068	0.005	0.065	0.092
	P	0.963	0.576	0.333	0.315	0.379	0.440	0.351	0.885		<0.001	0.948	0.981	0.948	0.948
	lower	-0.382	-0.617	-0.151	-0.595	-0.571	-0.570	-0.076	-0.327		0.884	-0.469	-0.389	-0.306	-0.322
	upper	0.473	0.209	0.618	0.214	0.237	0.251	0.654	0.399		0.993	0.326	0.400	0.450	0.519
Exp2	rho	0.122	-0.263	0.252	-0.330	-0.221	-0.295	0.371	-0.030	0.966**		0.059	0.133	0.186	0.194
	P	0.918	0.576	0.333	0.134	0.379	0.203	0.351	0.885	<0.001		0.779	0.703	0.703	0.703
	lower	-0.345	-0.620	-0.195	-0.669	-0.578	-0.654	-0.024	-0.418	0.884		-0.331	-0.257	-0.179	-0.226
	upper	0.517	0.216	0.613	0.085	0.216	0.148	0.681	0.349	0.993		0.425	0.472	0.526	0.556
N_{miR}	rho	0.121	-0.061	0.266	-0.673**	-0.404*	-0.628**	0.132	-0.416*	-0.068	0.059		0.951**	0.855**	0.727**
	P	0.918	0.843	0.333	0.001	0.101	0.002	0.617	0.115	0.948	0.779		<0.001	<0.001	<0.001
	lower	-0.291	-0.466	-0.186	-0.809	-0.707	-0.773	-0.339	-0.710	-0.469	-0.331		0.855	0.689	0.361
	upper	0.508	0.374	0.665	-0.425	-0.004	-0.389	0.552	-0.057	0.326	0.425		0.982	0.939	0.879

m150	rho	0.194	-0.133	0.247	-0.709**	-0.464*	-0.667**	0.206	-0.365	0.005	0.133	0.951**		0.897**	0.762**
	P	0.914	0.800	0.333	<0.001	0.088	0.001	0.617	0.146	0.981	0.703	<0.001		<0.001	<0.001
	lower	-0.234	-0.526	-0.190	-0.870	-0.721	-0.838	-0.211	-0.704	-0.389	-0.257	0.855		0.763	0.454
	upper	0.575	0.318	0.634	-0.446	-0.113	-0.385	0.563	0.049	0.400	0.472	0.982		0.945	0.897
m160	rho	0.279	0.027	0.243	-0.709**	-0.468*	-0.675**	0.096	-0.180	0.065	0.186	0.855**	0.897**		0.684**
	P	0.574	0.897	0.333	<0.001	0.088	0.001	0.647	0.584	0.948	0.703	<0.001	<0.001		<0.001
	lower	-0.133	-0.403	-0.183	-0.850	-0.748	-0.816	-0.296	-0.554	-0.306	-0.179	0.689	0.763		0.373
	upper	0.619	0.472	0.621	-0.456	-0.125	-0.405	0.498	0.231	0.450	0.526	0.939	0.945		0.871
L3utr	rho	0.295	-0.079	0.034	-0.540**	-0.417*	-0.514**	0.151	-0.461*	0.092	0.194	0.727**	0.762**	0.684**	
	P	0.574	0.843	0.874	0.009	0.101	0.017	0.617	0.115	0.948	0.703	<0.001	<0.001	<0.001	
	lower	-0.128	-0.499	-0.413	-0.762	-0.688	-0.764	-0.217	-0.709	-0.322	-0.226	0.361	0.454	0.373	
	upper	0.642	0.340	0.416	-0.201	-0.059	-0.140	0.510	-0.100	0.519	0.556	0.879	0.897	0.871	

This table is calculated based on all the genes involved in Imd pathway;
Lower and upper indicates the confidence intervals of the correlation;
*, $P < 0.05$ when bivariate correlations of different factors were calculated;
**, $P < 0.05$ after the FDR correction at q = 0.05.
