# Supplementary material for: Evolutionary rate patterns of genes involved in the Drosophila Toll and Imd signaling pathway
Source: BMC Evol Biol. 2013 Nov 8;13:245. doi: 10.1186/1471-2148-13-245 (PMC3826850; doi:10.1186/1471-2148-13-245)
Supplement: Additional file 2 — Multiple sequence alignments of the Toll and IMD pathway orthologs across six Drosophila species. Alignments of the protein-coding sequences were performed using PRANK. Stop codons were removed from the ends of the alignments. Dots indicate the same nucleotide and “–”represents a missing nucleotide relative to sequences in the first row. [file 1471-2148-13-245-S2.pdf]

Multiple alignments of new annotated genes:

1.FADD

```
D. ananassae    ATGTCCAAGGTAAATGGCTGGAATGGGTGTGGAGCGGGAACGCACGGAAATGGGAGCAGTGCCTCCTTACCACCGACT
D. melanogaster ------.T..CGT..........C..A..C...AT...CGT...G.TG..C..A....AC.G.G..A..GGTTTAT.C
D. sechellia    ------.T..CGT..........C..A..C...AT...CGT...G.TG..C..A....AC.G.G..A..GGCTTAT.C
D. simulans     ------.T..CGT..........C..A..C...AT...CGT...G.TG..C..A....AC.G.G..A..GGCTTAT.C
D. erecta       ------.T..C.T..........C..A..C...AT...CGT...GATG..C....T..AC.G....A..GGCTTAT.C
D. yakuba       ------.T..CGT..........C..A..C...AT..ACGTT..G.TG..C.......AC.G....A..GGTTTAT.C

D. ananassae    CCTAAATACTTGCGCAGCACAGAACTCCGGCGCGTTGAGGACAACGACATTTACCGGCTGTCCAAAATCCTTGACGAA
D. melanogaster T.G..G..T.CT....A...G..G..G..A..............T..............TG.......A..A..T..G
D. sechellia    T.G..G..T.CC....A...G..G..G..A.....C........T..............TG.......A..A.....G
D. simulans     T.G..G..T.CC....A...G..G..G..A.....C........T..............TG.......A..A.....G
D. erecta       T.A..G....CC...........G..G........C...........T..G........TG..........A..T..G
D. yakuba       T.A..G....CC........G..G..G..A.....C.......................TG..........A......

D. ananassae    AACGCCTGTTGGCGCAAGCTCATGTCGATAATACCGAAGGGCCTGGATGTACAGACTTCAAGCGCTGCCGGCGGTTTA
D. melanogaster ...T.A..C........AT.G........C.....C......A.......G...G.C.GC....GA.....AT.C..G
D. sechellia    ...T.A..C........A..G........C.....C......A.......G...G.C.GC....GA.....AT.C...
D. simulans     ...T.A..C........A..G........C.....C......A.......G...G.C.GC....GA.....AT.CC..
D. erecta       ...T....C.....T.....G.....C..T.....C......A.......G..AG.C.GC....GA.....AA.C...
D. yakuba       ...T.T..C...........A.....T..C.....C......A.......G...G.C.GT....GA.....AAAC...

D. ananassae    AACTTTCCAAATGCCATAAAGAAAGGATTCAAGTACACAACACAGGACGCACTGCAAATTGACGAGGCGGCTAACAGA
D. melanogaster ..T.....GGCG.AA..C..A..G.....T........TG.G.......TGT.C..G...........T..A...C..
D. sechellia    ..T.....GGCG.AA..C..A..G.....T........TG.G.......TGT.C..G...........T..G...C..
D. simulans     ..T.....GGCG.AA..C..A..G.....T........TG.G.......TGT.C..G...........T..G...C..
D. erecta       ..T.....TGCG.AA..C..A........T..A.....TG.G......ATGT.C..G...........T..A...C..
D. yakuba       ..T......GCG.AA..CC.A........T..A..TT.TG.G...A...TTAGC..G...........T..A.G.C..

D. ananassae    CTAGCACCCGACCAGAGCAAGTCCCAGATGATGATCGATGAGTGGAAGACGTCTGGAAAATTGCACGAGCGGCCAACA
D. melanogaster ...C.G..G.....A........G..............C...........C.....C..GC.CA..........C..G
D. sechellia    ...C.G..G.....A.......................C...........C.....C..GC.CA..........C..G
D. simulans     ...C.G..G.....A.......................C...........C.....C..GC.CA..........C..G
D. erecta       ..GC.G..G..............G..A...........C...........C..C..C..GC.CA..........T...
D. yakuba       ..GT.G..A..............G..A...........C........A..C.....C..GC.TA..........C..G

D. ananassae    GTGGGCGTATTGCTCCAGCTCCTGGTTCAGGCGGAATTGTTCAGTGCAGCCGACTTCGTTGCGCTGGACTTCCTTAAC
D. melanogaster ..T..G...........A..T.....G.....A..GC.C........G..A.....T..G..A..A........A..T
D. sechellia    ..T..G..G........A..T.....G.....A..GC.C........G..A.....T..G..A..A........A...
D. simulans     ..T..G------------------------------------------------------------------------
D. erecta       .....T..G........A..T.....G.....A..GC.C........G..A.....T..G..C..T........G..T
D. yakuba       .....G..G........A..T.....G.....A..GC.C........G..A.....T..G..A..A........A..T

D. ananassae    GAGCCCAAGCCAGATCGCCCCAGCGACGGACCAGCAGCACCTATAAGT------------------------------
D. melanogaster ...T...CC..T.CC..G..TGTT.....T..C.GT..G.TC.....C------------------------------
D. sechellia    ...T...CC..T.C...G...GTT.....T..C.GT..G.TC.....C------------------------------
D. simulans     ------------------------------------------------GGTGTTGCTCCAACTTCTGGTGCAAGCAGA
D. erecta       ...T...CC..T.C...G..GGTT.....T..T.GT..G..C..T..C------------------------------
D. yakuba       ...T...TC..T.T...G..G.TT.....T..C.....G..C..C..C------------------------------

D. ananassae    ---------------------------------------------CTGGACTTAACGGAGCTTCTTGACGAAACCATG
D. melanogaster ---------------------------------------------..T---------.....G..G..A..GGAA...
D. sechellia    ---------------------------------------------..T---------.....G..G..A..GGAA...
D. simulans     GCTCTTCAGTGCGGCAGACTTTGTGGCACTAGACTTCCTAAACGC..T---------.....G..G..A..GGAA...
D. erecta       ---------------------------------------------..----------.....G..G..A..GGAA...
D. yakuba       ---------------------------------------------..----------.....G.CG..T..TGA....

D. ananassae    GATGTTGACGGCGACGGCCTCAAT------------CCGAGTCCTTCAAATGTG------GCTGCCCAGGGAAGTATT
D. melanogaster ..A..G...AA...G..G..G.GCCTCAAATACCAGT.A..CA..G.C.C.C.TGGGGCA.AC..T..A..T..CG..
D. sechellia    ..A..G...AA...G..G..G.GCCTTAAATACCAG..A..CA..G.C.C.C.TGGGGCG..C..T..A..T..CG..
D. simulans     ..A..G...AA...G..G..G.GCCTTAAATACCAG..A..CA..G.C.C.C.TGGGGCG..C..T..A..T..CG..
D. erecta       ..A..G...AA...G..AG.G.GCCTCAAATACCAGT.A..CA..G.CGC.C.TGGGGGA.GC..T..A..T..CG..
D. yakuba       ..A..G...AA...T..G..G..CCTCAGATACCAGTTA..CA..G.CTC...TGGGGGA.GC..T..A..T..CG..

D. ananassae    GGCCTTAATTTGGACAACTTTGACAAGCAGATTATGCCGCGGAACAAAAGCCTGCCCCAACCGTCTGAGACTGTTCCT
D. melanogaster .....G..CC.............A..AG.C...G..AG....G....G..TG.......G.....A.GA.ACACC...
D. sechellia    .....G..CC.............A..AG.C...G..AG....G....G..TG....G..G.....A.GA.ACACC...
D. simulans     .....C..CC.............A..AG.C...G..AG..A.G....G..TG....G..G.....A.GA.ACACC...
D. erecta       ..A..G..CC......C..A...A..AG.C..GG.AAG.A..G....G..TG.......G.......G.GAC..C...
D. yakuba       .....G..CC......CT.....A..AG.C..GG..AG.A..G....G..TG.A.....GT....A.G..TC.CC.GC

D. ananassae    CCCATAGCGCCGCCCCGTACCTCTCGTCTGCATAGGGACGCAACCAGCTCA---------AACAATGCCACCGTATCG
D. melanogaster ...........A..T..A------..A.A...A...AGTA.T..T.A...CAACTTTGCA.CA------------.T.
D. sechellia    ...........A..T..A------..A.A..CA...AGTA.T..T.A...CAACTTTGCC.CA------------.T.
D. simulans     ...........A..T..A------..A.A..CA...AGT..T..T.A...CAACTTTGCC.CA------------.T.
D. erecta       ........T..A..T..A------..A.A..CA....GTA.T..A...A.C---------.GT------------.T.
D. yakuba       ...T...T...A..T..A------..A.A..CAC..A.TAAT..T.....C---------.G.------------.TC

D. ananassae    ACT---GCGACCGGGTCTACGTCGGCGACCACGCCCAATATACCTAATCTAACAATTCTCAACCGCACCGAACAATTA
D. melanogaster ..A---.GC.....AA........A......TC..G...G.G..C.....G...........T.C..G.......A.T
D. sechellia    ..A---.GC......ATC......A......TC..G...G.G..C.....G...........T.C..G.......A.T
D. simulans     ..A---.GC......A.C......A......TC..G...G.G..C.....G...........T.C..G.......A.T
D. erecta       .T.---.G.C.A.TAA.CGT.C.....T...TC..G...G.G..C..C..G...........T.C..G.....C.A.T
D. yakuba       ..CGCA.GC...A.AA.C.T...........TC..A...G.G..C..CT.G........A..T.C..G.......A.T

D. ananassae    GCGGAGCAGGAGAATCAGCCTCGACCGCAAAACATCCCAAATTTGTCCATACTCATAGCAAGCTCAACATCTTCAATG
D. melanogaster CA.....CA.T.CTG.....A......ATG........CG..C....T........TT.G.A...TGGTGA..TGCG.
D. sechellia    CAA....CA.T.CTG.....A.......TG........CG..C....T.....T..T..G.A...TGGTGA..TGGG.
D. simulans     CAA....CA.T.CTG.....A.......TG........CG..C....T........T..G.A...TGGTGA..TGGG.
D. erecta       CA......A.TCCTG...T.A.......TG..T......G..C....T........T........TGGTGA....GG.
D. yakuba       CA......A.TTCTG.....A.....A.CG..T......G..C....T.....TG.T....A...GGGTGA....GG.

D. ananassae    ACAACGCAATCCACTTCTTTGGGGCAGCCAAACAGTCAACAGGATGGCTCTGCGGACACACCAAACATACCTAAAATT
```

```
D. melanogaster G....TGTG..GGACAAC------------CCA..CA.CAGAAC.A....AA....TC.G..T.....T..GCG...C
D. sechellia    G....TGTG..GGACAAC------------CCA..CA.CAGAAC.A....AA....TC.G........T..GCG...C
D. simulans     G....TGTG..GGACAAC------------CCA..CA.CAGAAC.A....AA....TC.G........T..GCG...C
D. erecta       GA...ATC...GGACAAC------------.CA..CA.CA.A.CCA....AAG...T..GA.......T..GCG...C
D. yakuba       G....TTTG..TGGCAAC------------.CA..CA.CA.ATCCAA...AA.A..TT.GA..........GCG...C

D. ananassae    ACGCTTTTGATTGAGAACTCTGCCCAGATAAGCCAG---------------------------CCAACGACTGAAGCA
D. melanogaster ..T.....A.....T..T....GAG.TG...A.TCTCGACCAAATCACGCTCCAGCGAAAGCTT.....G..ACCA..
D. sechellia    ..T.....A.....T..T....GAG.TG.....TCTCGACCAAATCACGCTCCAGCGAAAGCT......G..ACCA..
D. simulans     ..T.....A........T....GAG.TG.....TCTCGACCAAATCACGCTCCAGCGAAAGCT......G..ACCA..
D. erecta       ..T.....A.....T..T---------------TCTCGACCAAATCACGCCGCAGCGAAAGCT........GACC---
D. yakuba       ..T.....A.....T..T.....AG.TT.....TCTCAATCAAATCACGATGCAGCGAAAGCT......G..ACCA..

D. ananassae    GCGGAAACGAGCTTAAACAATTTGCCCATGATAAGCGCATTAAATCTGAACACTAGCAACGGAGAGTCCCCGCCCCCT
D. melanogaster T.AACGG......C......C.....A...........C..G...A.A.GT.AAG...G.A.G...A..TT..GT...
D. sechellia    T.AACGGT.T...CG.....C.....A...........C..G..CA.A.GT.AAGA..G.A.G...A.TTT..GT...
D. simulans     T.AACGGT.T...CG.....C.....A...........C..G..CA.A.GT.AAGA..G.A.G...A.TTT..GT...
D. erecta       ------------CC......C.....A...........C..G...A.A.GT.A.GA..G...G...A.TTTA.GT...
D. yakuba       T.AATTG...CA.C......C.....A...........C..G...A.A..T.A.GA..G...G...A.TTT..GT...

D. ananassae    AACAGT---GACAGTGACAGCAGCCTGAGTAACGACGAAGATGAA--------------------GGAGATGCTGAC
D. melanogaster G.G..CAGAAG...CTC.......T....C..A..T..T..C..TGATAACGATGGTGAGGAGGAT.....G.AA..G
D. sechellia    G.G..CAGAAG...CTC..T....T....C..A..T..T..C..TGATAACGATGGTGAGGAGGAT.....G.AA..G
D. simulans     G.G..CAGAAG...CTC.......T....C..A..T..T..C..TGATAACGATGGTGAGGAGGAT.....G.AA..G
D. erecta       G.G..CAGAAG....TC.......T....C..A..T..C..C..TGATATCGATGGTGAGGAGGAT...---------
D. yakuba       G.G..CAGAAG....TT.......T.A..C..A..T..T..C..TGATATCGATGGGGATGAGGAT.T...G.AA..A

D. ananassae    GATCCGGATGCATCGCTGCGAAACCTGAGCAACTCGGAGCAACAGAACTCTAACAACGATTCCAGCCTGACAACGGTG
D. melanogaster T.C..A.....C.TCT...C...T.....T.....A.....G....G...A.....T..C................T
D. sechellia    T.C..A.....C.TCT...C.........T.....A.....G........A.....T..C................T
D. simulans     T.C..A.....C.TCT...C.........T.....A.....G........A.....T..C................T
D. erecta       ------.....C.TC...A........T.T.....A.....G........A.....T..C..T...T.........T
D. yakuba       T.C..A.....C.TC....C.......T.T....T......G........A.....T...................T

D. ananassae    ACTGGCACCAGCGGTGATAACAGCTTCGAAATGACCAACGACTCAAGTTCGACCTCGAACGACGATTATGGCGGAAAT
D. melanogaster ...........T..............T..GC.A...........C..C..C.....A...........C.CTT.C..C
D. sechellia    ..........................T..GC.............C..C..C.....A...........C.CTT.C..C
D. simulans     ..........................T..GC.............C..C..C.....A...........C.CTT.C..C
D. erecta       ..........................T..GC.............C..C..C.....A...........C.CTT.C..C
D. yakuba       ..........................T..GC.............C..C..C.....A...........C.CTT.T..C

D. ananassae    ATTCCAAACCTGAGTGAACTGCAGCCG
D. melanogaster ......G..T.......G.......AA
D. sechellia    ......G..T.......G.......AA
D. simulans     ......G..T.......G.......AA
D. erecta       ......G..T.......G.......AA
D. yakuba       ......G..T.......G.......AA
```

2.Dif

```
D. melanogaster ATGTTTGAGGAGGCTTTCGGCGATATACAGGAAATTATCAATGCCAGCATGGAGTTGAATGGTGGCGCCACCGGCGGA
D. sechellia    ...........C.................................................G................
D. simulans     ...........C..................................................................
D. yakuba       .................T......T..G..............GTGAG---------.............TT..A.A.
D. ananassae    ---------------------------------------------------------------------.TG.AG.AG
D. erecta       .................T...........................................................A...

D. melanogaster GGCAGTGTGGCAGGAGCTGTTGGCGGAGGCGGTGCTGCACATCACATATTATCGCAGTCGACTTCCCTGCCGGTAATG
D. sechellia    ....CG...........................G............................................
D. simulans     ....CG...........................G............................................
D. yakuba       A...A.........C..A......A...........T.....AG...GA......C....TT................
D. ananassae     A..GA..GC..CACG.GG.GC...CCTCCGCCGT.C.G...G.CA.....CC.......C..A...............
D. erecta       .......C.........A...........................ATA.............................

D. melanogaster CCGTCGCACATTCCGCTCCACCTGCAGAATCAGAATATGAATCAG---------AATCTGCCCGAGCCAAGT---GCA
D. sechellia    .......................A...................A---------..........T.......---..T
D. simulans     .......................A...................A---------.....................---..T
D. yakuba       ..CGTA---..G..T.AAATGTC.------------------.TTACTCCACGA..C....T...A..G.A.---.A.
D. ananassae     T........C.G..A.....AT.C------------------...AGCCCGGCGCCG......A....G..CACCAA.
D. erecta       ..............A........A------------------..---------.....................---...

D. melanogaster AGAAGTGGTCCCCACCTGCGTATCGTGGAGGAGCCGACAAGCAATATAATCCGCTTTCGCTACAAATGCGAGGGTCGC
D. sechellia    .................C......................A........................G...........
D. simulans     ....................A.................G...........................G...........
D. yakuba       ---G...A.....A....C.........C.......GG.....G....T..T.........G.............
D. ananassae     ---.....A...........A.................G.A..................G.....G........C...
D. erecta       ---.....A.....T....C.........C.......G.....................................

D. melanogaster ACCGCCGGTTCGATTCCGGGCATGAACTCCAGCTCGGAAACGGGCAAGACCTTTCCCACCATCGAAGTGTGCAACTAC
D. sechellia    ..T...........................................................................
D. simulans     ................................T.............................................
D. yakuba       ........C.............AA.......AA..AA.T---T.................G........CTG......
D. ananassae     .....A..A......................A.A....C.A...........C.....G....G...........
D. erecta       ........C................G.....T.....T...........T..C..........G...........

D. melanogaster GATGGACCCGTCATCATCGTGGTCTCCTGTGTGACGAGCGACGAGCCCTTCCGCCAGCATCCCCACTGGCTGGTCAGC
D. sechellia    ..C.................................................A.....G..............
D. simulans     ..C.................................................A.....G..............
D. yakuba       C.C...AAG....C....A.A..A...............A..AT.....AT..T..A...........T........
D. ananassae     ..C..T.....TG..G.G..A..G.....C.....C........A....A.........C..................
D. erecta       ..C.............................................AT.........................

D. melanogaster AAGGAGGAGGCGGATGCCTGCAAGTCGGGCATCTACCAAAAGAAATTGCCGCCAGAGGAGCGGCGGCTGGTGCTCCAA
D. sechellia    .....................T........................................................
D. simulans     .....................T.....................G...............A......T..........
D. yakuba       ...........T........T.........G.......G....T.....A...........................
```

```
D. ananassae    ..............C......CG...C....C....TCC...CGCC.C..T..C.......................G
D. erecta       ......................................G..............G........................

D. melanogaster AAAGTGGGCATACAGTGCGCCAAGAAGCTGGAGATGCGCGACTCGCTGGTGGAGAGGGAAAGGAGAAACATCGATCCC
D. sechellia    ...........................................................G.A...G............
D. simulans     .............................................T.............G.A...G............
D. yakuba       ............A......T.......TC...T...TT...G.......C..T....C.G.....GGGA..T......
D. ananassae     ..G........C.............................T..C...C...........C...TC..TG.G.....T
D. erecta       ...........................................................G...C.G............

D. melanogaster TTCAATGCCAAATTCGATCACAAGGACCAGATCGACAAGATCAATCGGTATGAGTTGCGCCTCTGCTACCAGGCCTTC
D. sechellia    ...........G..T...............................................................
D. simulans     ..............................................................................
D. yakuba       ..T...................T......TG.......................C............TT.........
D. ananassae    ..TGGA..T...................................C.....C...C....T.............G....
D. erecta       ..............................G.......................C.......................

D. melanogaster ATCACAGTGGGCAACTCAAAAGTGCCCCTGGATCCCATCGTATCCTCACCGATTTACGGCAAGAGCAGCGAACTGACC
D. sechellia    .................G....................A........C..............................
D. simulans     .................G.......................G.....C..............C........G......
D. yakuba       .....T....AT..GAAG...........CA.G..............G..T...C...........TA...G......
D. ananassae     .........AA...AAGCCG............C...G.T..G...A.C.......T...........A...G......
D. erecta       .................G............A.G..............C...................A...G......

D. melanogaster ATCACCCGGCTGTGCAGTTGCGCGGCCACTGCGAACGGCGGCGACGAGATCATCATGCTGTGCGAGAAGATTGCCAAG
D. sechellia    ............................G............GA...................................
D. simulans     ............................G............GA...................................
D. yakuba       ....T..................A.................G.....C.................T..C...A.....
D. ananassae    ...T..AA.A.C....CC...T.C...C.G..TTC...T...TCT....................C.G.G.C......
D. erecta       .......................................................................C......

D. melanogaster GACGACATCGAGGTGCGATTCTATGAGACGGACAAGGATGGACGGGAGACGTGGTTCGCCAACGCAGAGTTCCAACCC
D. sechellia    .......................................................A.........C............
D. simulans     .......................................................A.........C............
D. yakuba       ................AT...............G.......G............A.......A..C..T...A..T..
D. ananassae    ...............GT......C..A.......GCC....C.......TT...AGTCAG..T..G......A.G...
D. erecta       .................G.....C.................G.......................C............

D. melanogaster ACGGACGTGTTCAAACAGATGGCCATAGCTTTTAAGACGCCGCGCTACAGGAACACCGAGATCACACAAAGTGTTAAT
D. sechellia    .............................A..C......................................C......
D. simulans     .............................A..C.............................................
D. yakuba       .............GG........T..........................................A........T..
D. ananassae    .....T..C.....G...........TA.G..C...GT...C.......A...TC.A.........GC..A.......
D. erecta       ..............G.................C......................................C......

D. melanogaster GTGGAGCTGAAACTAGTGAGACCCTCGGATGGAGCGACGAGTGCCCCACTGCCGTTCGAGTACTACCCGAATCCAGAA
D. sechellia    ..............................................................................
D. simulans     ..............................................................................
D. yakuba       ...A.T.....G..T..............C............T.........T...........T............G
D. ananassae    ..............T..AC...........AAG.....C.....G..C....A...............C..AA....C
D. erecta       ...........G.................C.......................A........................

D. melanogaster CTCCTAACCAAACACAATCGCCGTGTTGCCCAAAAAACAGTTGAAAGCCTAAAGCGCAGTCTCATGAGCACCAAT---
D. sechellia    .....T...........C.........................................................---
D. simulans     ...............C.C.........................................................---
D. yakuba       .....T....TG.....C.....AA.....................A.........T......GAA.C....G..TTC
D. ananassae    A...GC.T.C.G....C...TAAAC.G.GTTCTC....GA..........T.......C...A....A...AG.A---
D. erecta       ...........G.....C.....G.................C..............................G..---

D. melanogaster ---------TTACATCCATCCAAGCAGGTTAAAACCTCCAGCCAGTATACGATATTTTCCAAGCCACAAATTGCGACC
D. sechellia    ---------.............................T..................C...C..........A.....
D. simulans     ---------................................................C...C................
D. yakuba       TATTCCAATA.CT............T......GC..............---------C...CT..........T....
D. ananassae    ---------...T..............T.C.G...G.....T.CAAT.T..G.GG.CC..CC...T..GCCA..ACAG
D. erecta       ---------..CT............T......G.A................G.GG..C...CT......G........

D. melanogaster ACAACACCGCAGACACAGGTGTCGCCTGGTATGCCTTTGATGTTTCCCGGCGGAAGTCCCAAT---TTTGTGCAGGAT
D. sechellia    ...G.......A..G...T.............A..............G..T............---............
D. simulans     ...G.......A..G...T............................G..T............---............
D. yakuba       ...G.......A...G.........................C.....GA..A.T........A---....A.....C.
D. ananassae    GGCCAG..A...G.T...........CAACC..G.GA..C.......T..AA.G......C.GCAG.A......A..C
D. erecta       ...G.......AG..................G.........C...........T.........---..C.........

D. melanogaster ATCAAGATGGAGAATGGATTCATGGATGTGGATAGCCAGAGTAGCCAGTGTCCATCAGTGGAACGCAATTTCGCTTCG
D. sechellia    .....................................G..................G..............T..A...
D. simulans     .....................................G..................G..............T..A...
D. yakuba       ........T...........TCC....T.C..C......---------............A.TTC.......C.A...
D. ananassae    ......C.....CCG.CCG.AC.....A....C..T.G.....AT.CC..C..CC...G.ACCTTT..C.ATC.....
D. erecta       ....................T.....CA....C....................G....................A...

D. melanogaster CCTCGATCCAACTGCAGTACAGTGGATAGTATTCCGCCGATGCAAATGGGGCAAAATCAAACTCAT------CTTTAT
D. sechellia    ..A..........................................C.C..............G...CTTTAT......
D. simulans     .............................................C.C..............G...------......
D. yakuba       ...ACG......AA.---------A....C....T............C.T...GC.G.C..A....------T.C...
D. ananassae    ....AT..T.C......C........C........A.....T..G..C..C..G...AC..AC...------------
D. erecta       G..A.G...........C...........C..A..............C.....G..G....A....------..A...

D. melanogaster TTGCCAGATGCCACAAACTTCACATTTAATGGGAATTTCGCATCGCCTTCGTCCAATTGCAGTACAGTGGATAGCATT
D. sechellia    .................T...G..........................CA............................
D. simulans     .....................G..........................CAA...........................
D. yakuba       ------------------------------..............A...CA..T...CGA..A.CA.A.CA..------
D. ananassae    ------------------------------------------------------------------------------
D. erecta       ...........T.........C..........................CGT.....C.....C...............

D. melanogaster CCGCCGTTTCAAATCGGACAGAGGAATAACCATATGTATCTGCCGGAGAATTCAAACTTTCCGGTAAACGGCTGTAGT
D. sechellia    .......................A.........T................C..............T............
```

D. simulans   .................................T................C..............T............
D. yakuba   ------.A..........A....C.......C.C.A...........A...C.T.GT...ATA.C.....A.G.....
D. ananassae   ---------------------------------T.A.....C..A....CC..G...C.CT.CA.G.CTTC...C...
D. erecta   .......................C.........C.C............GG...............T.....G......

D. melanogaster CCTACCCATTTCAGTGGTGGCTCTATGACACCAATCAAT---------AATAACAACAATGTACTAATTAATAACAAT
D. sechellia   .....................A.C.....C.........---------...............T.......C......
D. simulans   .....................A.C.....T.........---------..............................
D. yakuba   A.....A.G..............C.....T........C------------G.T.....CAC....G...........
D. ananassae   .....GA.C..T.A......A..C.....T..C.....CCAGAATAAC........T...A.GT.G..G........C
D. erecta   ..C....................C.....T........C---------...........CA....GG...........

D. melanogaster AAC---------------AATGACTTTTAAGTCAGAAAATGAGTGCCATAAGCATACCGCCA----------------
D. sechellia   ..T---------------...........G............C...................----------------
D. simulans   ..T---------------..----------------
D. yakuba   ...---------------......A.C.......GA...T.AG...........T.......----------------
D. ananassae   .TTAACTATTTGGAGTTG...A.TG..GGCGTCAT...G....A......G..TCCCA..G.GCCGAGCAACCAACAC
D. erecta   ...---------------........C........A..................TC......----------------

D. melanogaster CAAGGAAATTTTGGCATTAAACAGGTATATCAACAGACTCAG---CAGTTCCTGCCGCAATTGCAGCCAGAGTCCATT
D. sechellia   .....TG......................C........C...---.................C...............
D. simulans   ....C.G...................G..C............---.................C...............
D. yakuba   ......T.......T..-------------G.........T.---...A...A...T.....A.CA------------
D. ananassae   ....T.T.CCAACAGCAG------------..G.....C...TAT...ACGGAA..AA.TGCAT.T------------
D. erecta   ..............T..C........G..C.......G....---...........T.....C..C..G.........

D. melanogaster CCATATCTGGCCCAATCCCATCCAGAACAGTCGCAGTACCAA---------CAGCAA------------------CAG
D. sechellia   ..G.T.....................................---------......---------------------
D. simulans   ..G.T.....................................---------......---------------------
D. yakuba   ------------------G.A..CATT..AAATGT.G....CACCCACGATG.A---------------------AC.
D. ananassae   ------------------.TG..G.CT...AAT......TCCATCCAGGCGG....GCACCAGTTCACCCAGCCCACT
D. erecta   ......T.............G....................C---------......CATCAGCTGCAGCAGCCA...

D. melanogaster CAGCCACAGGAGCAGCAACCTCCAGCAGATGAACCCACCCAATCGTTTAGTGATCTAATTTCC------AGCAGCATA
D. sechellia   ---------C.............G.............T.........A...............---------...G.G
D. simulans   ---------C.............G.............T.........C...............---------...G.G
D. yakuba   ...GTCA.CC....AA.T.AG.T.---------.AG..A.C......C............A.T------.........
D. ananassae   ....TG...C.A...ACCGAGGGC---------.A.GT...G..T..C..C.....G...CTGAACGGC.CACCG..G
D. erecta   .........C........G...TG.............T...G.....C............AGT------G........

D. melanogaster GGCATGGCGCCCATTGACACCAGCGAACTTATCCAGGACATTGAGGCAGAACTCAATAGCTTGGGAATT---------
D. sechellia   .CG.CCAT...........A....................................C............---------
D. simulans   .CG.CCAT...........A.........C..........................C............---------
D. yakuba   .A......A............GA.........A.GC.G........A...T.....CTT.......C..---------
D. ananassae   .A.T...GCT....C...TT.------------------.....T.AGACC..T.GCG..C....GC.GGCAGGCGAC
D. erecta   ..............C.......A..............G.........T..G.....C.....A......---------

D. melanogaster ---CAGCCATTCAAA
D. sechellia   ---.........G..
D. simulans   ---............
D. yakuba   ---..AAA.CCA---
D. ananassae   GGAGCTGTAGGTTTTGGGGGAACCCCCCAAATGAACAATAATTTTGCCAAT.A.---
D. erecta   ---...A.......G

3.Dl

D. ananassae   ATGTTTCCCAACCAGAACAACGTAGCCGCGTCTGGGCCAGGCCCGGCGGCTGATGGCCAACAGAGCCTCAACTACAAC
D. melanogaster ........G...........T.G......TC......AG..T..A..A.T............................
D. sechellia   ........G.G...........G......TC......AG..T..A.................................
D. simulans   ........G.G...........G..G...TC......AG..T..A...........G.....................
D. yakuba   ...........T..........G......TC......AGCTT..A........C........................
D. erecta   .....C..............T.G......TC......AG.TT..A.....C...........................

D. ananassae   GGACTGCCGGCC---CAGCAGCAGCAACAGCAGCAGCAGCAACAGCAACAGCAGCAACTGTCACAGTCGTCGAAGAAT
D. melanogaster ..C.....C...---...........G---------------------------...T..G.......CA....A...
D. sechellia   ..G.....C...---...........G---------------------------......G.......CA....A...
D. simulans   ..G.....T...---...........G---------------------------...T..G.......CA....A...
D. yakuba   ........C...CAG...........G---------------------------..............CA....A...
D. erecta   ........C...---...........G---------------------------..............CA....A...

D. ananassae   GTGCGGAAGAAACCGTACGTGAAGATCACGGAGCAGCCGGCGGGAAAGGCCCTTCGCTTCCGTTACGAGTGCGAGGGT
D. melanogaster .....A.....G..C.....A........C..A..A.....A........G..AA.G..T..C..............A
D. sechellia   .....A........C.....T........C.....A..............G..G..G..T..C..............C
D. simulans   .....A........C.....T........C.....A..............G..G..G..T..C..............A
D. yakuba   ..............C.....A........T.....A..............T..G..G.....C.....A........A
D. erecta   ....................A.............................G..G..G.....C..............C

D. ananassae   CGATCGGCGGGTTCTATTCCCGGAGTGAACTCCACGCCGGAGAACAAGACGTATCCGACTATTGAGATAGTGGGCTAC
D. melanogaster ..C........A..C.....G..C........T.................C........A..C..A..T.........
D. sechellia   ..C........A..C.....G..C..A.....T.................C........C..C.....T.........
D. simulans   ..C........A........G..C........T.................C........C..C.....T.........
D. yakuba   ..C........A..C.....G..C........T.................C........A..C.....T.....T...
D. erecta   ..C........C..C.....G..C...................................C..C.....T.....T...

D. ananassae   AAAGGACGCGCCGTGGTCGTGGTCTCGTGTGTGACCAAAGATGTCCCCCATCGTCCCCATCCGCACAACTTGGTTGGC
D. melanogaster ..G...........A..T..T.....C..C..C..A..G...ACG..AT.......T.....C.....T..A......
D. sechellia   ..G..............T..C.....C..C..T..A..G...ACG..AT.............C.....T..A......
D. simulans   ..G..............T..C.....C..C..C..A..G...ACG..AT.............C.....T..A......
D. yakuba   ..G..............T..C.....C.....C.....G..CACG..AT.............C.....T..A......
D. erecta   ..G..............T..C.....C..C..C.....G..CACG..AT.............C.....T..A......

D. ananassae   AAGGAGGGTTGCAAGAAGGGTGTCTGCACTCTGGAAATCAACAGTGAAACCATGAGGGCCGTTTTCAGCAATCTGGGC
D. melanogaster ........C...........C.....T..A.....G...........G..A...C.A..G..G.....T..CT....T
D. sechellia   ....................C.....T..A.....G...........G..A...C.A.....G.....T..CT....T
D. simulans   ....................C.....T..A.....G...........G..A...C.A.....G.....T..CT....T
D. yakuba   ........C...........C........A..A..G..T..T.....G..A...C.A..A..G..T......T....T

```
D. erecta       ........C...........C........A.....G.....T.....G..A...C.A..A..G.....T...T....T

D. ananassae    ATCCAGTGCGTCAAAAAGAAGGACATCGAGGCGGCACTCAAGGCGCGTGAGGAGATCCGCGTGGATCCATTCAAGACT
D. melanogaster ........T.................T........G...........C...........T........G..T......
D. sechellia    ........T.................T........G...........C...........T........G..T......
D. simulans     ........T.................T........G...........C...........T........G..T......
D. yakuba       ........T.................T........G..G........C...........T........G..T......
D. erecta       ........T.................T........G...........C....................G.........

D. ananassae    GGCTTCTCGCACCGTTTCCAGCCCTCCAGCATCGACCTGAACTCGGTTCGGTTGTGCTTTCAAGTATTCATGGAGAGC
D. melanogaster .....T.....T..............G.....A..T.....T.....G..A...........................
D. sechellia    .....T.....T..............G.....A..T.....T.....G..A...........................
D. simulans     .....T.....T..............G.....A..T.....T.....G..A..A........................
D. yakuba       .....T.....T..............G.....T..T.....T.....G..A..........................T
D. erecta       .....T.....T..............G.....T..T.....T.....G..A...........................

D. ananassae    GAGCAGAAGGGTCGCTTCACCTCGCCCCTGCCGCCGGTGGTTTCGGAGCCCATCTTCGACAAGAAGGCCATGTCCGAT
D. melanogaster ..............A...........A...........A....................T.................C
D. sechellia    ..............A...........A................................T..................
D. simulans     ..............A...........A...........A....................T..................
D. yakuba       ..............A...........TT.A..............C.....T........T.................C
D. erecta       ..............A...........A.................C..............T.................C

D. ananassae    CTGGTCATCTGTCGGCTGTGCAGCTGCTCGGCCACCGTCCTGGGCAACACCCAGATCATCCTGCTCTGCGAAAAGGTG
D. melanogaster ...........C........T.................TT.C.............................G......
D. sechellia    ...........C...........................T.C.....T.......................G......
D. simulans     ........T..C.......................T...T.C.....T.......................G......
D. yakuba       ..T.....A..C...T.A.................T.....T.....T.......................G......
D. erecta       ..T........C...T.........................C.....T.......................G......

D. ananassae    GCCAAGGAGGACATCTCCGTGCGCTTCTTCGAGGAGAAGAACGGGCAGAGTGTGTGGGAGGCCTTCGGCGATTTCCAG
D. melanogaster .......................A.....T...........T..C........T...........T..T..C......
D. sechellia    .................T.....A.................T..T.....C..T...........T..T..C......
D. simulans     .................T.....A.................T..C.....C..T...........T..T..C......
D. yakuba       ..............TG.....A.A.....T...........T..C........T...........T..T..C..T...
D. erecta       .......................A.................T..C........T..........AT..T..C..T...

D. ananassae    CACACGGATGTCCACAAGCAGACTGCCATTACCTTTAAGACGCCGCGCTATCACACCCTGGACATCACAGAGCCCGCC
D. melanogaster .....................................................T........................
D. sechellia    .....................................................T........................
D. simulans     .....................................................T........A...............
D. yakuba       .....C...............................................T........G...............
D. erecta       .....C............................................C..T..............C.........

D. ananassae    AAGGTCTTCATACAGCTGCGACGTCCCTCGGACGGAGTCACCAGCGAGGCGCTGGCATTCGAGTACGTGCCATTGGAT
D. melanogaster .....T..T..T..A.................T.....T...........C...C.C...............A....C
D. sechellia    .....T..T..C..A.................T.....T...........C...C.C...............A....C
D. simulans     .....T..T..C..A.................T.....T...........C...C.C...............A....C
D. yakuba       ........T..C..A.................T.....T...........A...C.C..............GA....C
D. erecta       ...........C..A.................T.....T...........C...C.C...............A....C

D. ananassae    TCAGATCCAGCGCACTTGAAGCGGAAACGTCAGAAGACTGGCGGTGATCCCATGCACCTGCTGCTCCAGCAGCAGCAG
D. melanogaster ...................G..........................................................
D. sechellia    ...................G....................................A......T..............
D. simulans     ...................G..........................................................
D. yakuba       ...................G..........................................................
D. erecta       ...................G..........................................................

D. ananassae    AAACAGCAGTTGCAACAGAGCGACCATCCGGATGGCAGACAAACTAACATGAATTGCTGGAATGCTCAAAGTATACCT
D. melanogaster ........A...---....AT.....C.A..................................A.A....AC.....G
D. sechellia    ........A...---....AT.....C.A..................................A.A....AC.....G
D. simulans     ........A...---....AT.....C.A..................................A.A....AC.....G
D. yakuba       ............---....AT.....C.A.....C............................A.A....AC.....G
D. erecta       ..G.....A...---....AT.....C.A....................................C....AC.....G

D. ananassae    CCCATTAAAACCGAACCGCGAGATACCTCACCTCAACCCTTTGGTCTGGCGTACCGAGCCCCTCCAGAGCTAACGCCC
D. melanogaster ...........G.....AA..........G..A...........G..TT.T..T..G..G.....T.....C..A...
D. sechellia    ...........G.....AA..........G..A...........G..TT.T..T..G..G.....T.....C..C...
D. simulans     ...........G.....AA..........G..A...........G..TT.T..T..G..G.....T.....C..C...
D. yakuba       ...........G.....AA..........G..A..G........G..TA.C..T.....G.....T.....C..C...
D. erecta       ...........G.....AA..........G..A..G.....C..G..TT.T..T.....G.....C.....C..C...

D. ananassae    TCGCCGCAGCCCCTGTCGCCGTCGAGCAACTACAATCAGAACAGCACCCCCTCCCCCTACAACATGGGCTCTGCGGCC
D. melanogaster ...........G........A..............C..C........G.....G.............C...C....T.
D. sechellia    ...........G........A..............C..C........G.....G.............C...C....TG
D. simulans     ...........G........A..............C..C........G.....G.............C...C....TG
D. yakuba       .....C.....G........A..............C..C.....T..G.....G...........A.C...C....GG
D. erecta       ...........G........A..............C..C........G.....G............------....TG

D. ananassae    ACGCCCACCAACGGCCAGCAGCAGCAGCAGCAGCTGATGTCGCCCAACCACCCCCAGCAACAGCAGCAGCCGCAGCAG
D. melanogaster ...........T...---------........A.................T..A.....G..A------------...
D. sechellia    ...........T...---------........A....................A.....G..A------------...
D. simulans     ...........T...---------........A....................A.....G..A------------...
D. yakuba       ...........T...---------........A....................A.....G..A.......A...A..A
D. erecta       ...........T...---------........A....................G.....G..........A.......

D. ananassae    CCCCACCAGCAG------TACGGGGCCCCCGACCTGGGCAATAACTATAACCACTTTGCCCAGCAGGTGATGGCGCAG
D. melanogaster .AA..G.....A------..T..A..AA.T..T.....G.GC.....C..T.CG..............CC.T..C...
D. sechellia    .AA..G.....A------..T..A..AA.T..T.......GC.....C..T.CG..............CC.T..C...
D. simulans     .AA..G.....A------..T..A..AA.T..T.......GC.....C..T.CG..............CC.T..C...
D. yakuba       .AA..A.....A------.....A..AA.T..T.....G.GC.....C..T.CG........A.....CC.T..C...
D. erecta       .AG..G..A...CAGCAA..T.CA..A.....T.....G.GC.....C..T.CG..............CC.T..C...

D. ananassae    CAGCAACACCAACAGCAGCCGCAGCAGCAGCAGCAA---CAT---------------------------CAGCAGCAG
D. melanogaster .....G..G..T.......A......A.....C..G---...CAGCACCAGCAGCAACACCAACAGCAG.........
D. sechellia    .....G..G..T...------------------------...CAGCACCAGCAGCAACACCAACAGCAG.........
```

```
D. simulans      .....G..G..T......A...........T..G---...CAGCACCAGCAGCAACACCAACAGCAG.....---
D. yakuba        .....G..G..T.....C----------------------------------CAACACCAACAGCAG..A..A..A
D. erecta        ........G..G......A...............CAG...CAGCAGCACCAGCAACACCAGCAACAC..A.....A

D. ananassae     CAGCAACAG------------GCAATGCAATTCCACGGCAACCCCTTCGGAAATGCGGCAACTAGTCATTGGGAGTCC
D. melanogaster  .....G...CAACAGCAACAGT.CT......T..T.C...T.....T..C...C.T.GCGGC.A.AGC.....AAGT
D. sechellia     .........------------T.CT......T..T.C...T.....T..CC..C.T.GCGGC.A.AGC......AGT
D. simulans      .........------------T.CT......T..T.C...T..T..T..CC..C.T.GCGGC.A.AGC.....AAGT
D. yakuba        ..T......------------T.CT......T..T.C...T.....T..C...C.C.G.GGA.ACAG......AAGT
D. erecta        .....G...------------T.CT.........T.C...T.....T..C...C.C.G.GGC.ACAGC.....AAG.

D. ananassae     AAGTTC------------------GCGGGGGCAGCAGGAGGCACGGACATTAAGCCTGCTCCTGGCACCAGTCACAAC
D. melanogaster  ..A...TCGGCGGCAGCTGTTGCA..A.C.......ACT.CG..A.GAGCAGCA.....CAAC....AT..C------
D. sechellia     ..A...TCGGCGGCAGCTGTTGCA..A.C.......ACT.CG..A.GAGCAGCA.....CAAC....A...C------
D. simulans      ..A...TCGGCGGCAGCTGTTGCA..A.C.......ACT.CG..A.GAGCAGCA.....CAAC....A...C------
D. yakuba        ..A...TCGGCGACAGCTGTTGCA..A.C.......A.T.CG..A.GAGCAGCA...C.CAA.....A...C------
D. erecta        ......TCGGCGGCAGCTGTGGCA..A.C.......ACT.CG..A.GAGCAGCA.....CAAC....A...C------

D. ananassae     ATCAACAATCTCAGTAATCTCAACAATCCCTTCACCATGCACAACCTGCTGACATCCGGCGGAGGTCCTGGGGCTGGC
D. melanogaster  ---..T.......C.......................................G.............CAAC.C.
D. sechellia     ---..T.......C...........G......A......A..T..........G.............CAAC.C.
D. simulans      ---..T.......C...........G......A......A..T..........G.............CAAC.C.
D. yakuba        ---..T.......C.......................................A...........A.CAAC...
D. erecta        ---..T.......C.......................................G.............CAAC...

D. ananassae     AGCAATCCGCAGTGGAATCTGACCTCAAATCATCTGCACAACCAGCACACGCTCCACCAGCAACAGCAGTTGCAA---
D. melanogaster  .A.....T.........C......A...............T.....T.....T..T.....G......C.T..G---
D. sechellia     .A.....T.........C......C...............T.....T.....A..T.....G......C.T..GCAG
D. simulans      .A.....T.........C......C...............T.....T........T.....G......C.T..G---
D. yakuba        .A.....T...C.....C.....TA.T.............T..............T.....G......C.T..G---
D. erecta        .A.....T...C.....C......A.....................T.....T........G.....AC.T..G---

D. ananassae     ------------------CAGCAGCAGCAACATGTCCCGCCAATGCATCAGTATGATAACAGTGCCCCCGGCAACCCC
D. melanogaster  ------------------......---..G-----------CA...G.....C..C..T.C...G...---------
D. sechellia     CAGCAACAGCAGCAACAG.....A.....G-----------CAA..G.....C..C..T.C...G...---------
D. simulans      ------------------..........G-----------CA...G.....C..C..T.C...G...---------
D. yakuba        ------------------.............G------.A.CAA..G.....C..C....A...G...---------
D. erecta        ------------------..........G..G------.AGCA...G.....C..C....A...G...---------

D. ananassae     AGCAACAATAAT---AACAAC------------------AACAATAACCCC---------AACACGAGC---AACCAA
D. melanogaster  .C......C...------------------GCCAACCTT..T..C..TAATAACAATAAT..T..CGCTGGC..T...
D. sechellia     ........C..CAATCC....AACGGCAATGCCAACCTG..T..C..TAAT---------..T..CGCTGGC......
D. simulans      ........C..CAATCC....AACGGCAATGCCAACCTG..T..C..TAATAACAATAAT..T..CGCTGGC......
D. yakuba        ........C...---CC....AACGGCAATGCCAACCTT..T..C..TAAT---------..T..TGCTGGC......
D. erecta        .....T..C...---CC....AACGGCAATGCCAACCTT..T..C..TAAT---------...G.CGCTGGC......

D. ananassae     GCGGATAAT---GGTCCCACGATCAGCAATCTGCTGAGCTTCGATAGCGAACAACTGGTGCGCATCAACTCGGAGGAT
D. melanogaster  .........AAT..G..A...C............T............GG..GT......A...A.....A......
D. sechellia     .........AAT..G..A..............T..T............GG..GT......A...A.....A......
D. simulans      .........AAT..G..A................C............GG..GT......A...A.....A......
D. yakuba        .........AAT..G..A...C............C............GG...T....C.A...A.....A......
D. erecta        .........AAT..G..A...C............C............GG..GT......A...A.....A......

D. ananassae     CAGCAGATACTGCGCCTCAATTCGGAAGATCTGCAGATATCCAACCTATCCATATCTACG
D. melanogaster  ........................................G.....G.......C...
D. sechellia     ...................C...................G..T..G.......C...
D. simulans      ...................C...................G.....G.......C...
D. yakuba        ........G..........C...................A.....T.......C...
D. erecta        ...................C...................A..T..C.......C...
```

4.Dredd

```
D. ananassae     TATTTTTGTTCTTCCCCCGCAATTAACGAGATGGCCGGATCGAAACTTCTGAACCAAATAGACAGCATCGACACCAAC
D. simulans      -----------------------------........T.....C..GT...TT..TC.G....C......TCAG...
D. sechellia     -----------------------------........T.....C..GT...TT..TC.G....C...A..TCAG...
D. melanogaster  -----------------------------...........A..C..GT...TT..TC......C......TCAG...
D. yakuba        -----------------------------.............T..G....T...TC.G..G.C......T.AA...
D. erecta        -----------------------------......A.......T..G....T...CC.G....C...AC..CAAG..

D. ananassae     GATCTTCTCTTTGTGGAAAGGGATCTCAACTTTCCCCAAAAGATCAGTCTTTGTTTCCTCCTGTATGGCGATGACCAC
D. simulans      .....GA...AC.C....C.T..CA.G.....CG........G..G.C.....C..T..G..T........C......
D. sechellia     .....GA...AC.CA...C.T..CA.G.....CG........G..G.C.....C..T..G..T........C......
D. melanogaster  .....GA...AC......C.T..CA.G......G........G.GG.C..C..C..T..G..T........C......
D. yakuba        .....G....A..C....C.C..CA.G......GG.......G.GG.C........T..G..T..C..G..CA.....
D. erecta        .....GA...A..C....C....CA.G......G........G.GG.C..C..C..T..G.....C..A..CA.....

D. ananassae     TCGAGTGCCACGTACATTCTCCAGAAGCTGCTGGCTTTGGCTCCGGTAGCTTGGCAGCCGAGTGATCTTCTGCTGCAA
D. simulans      ...GA......C........G.....A..AT...TCA....A.GATC..AC.TATC..AA........A..CA.AA.G
D. sechellia     ...GA......C........G.....A..AT...TCA....A.GATC..AC.TATC..AA........A..CA.AA.G
D. melanogaster  ...GA......C........G.....A..TT....CA..A.A.GATC..AC.TC.C..AA........A..CA.AA.G
D. yakuba        ...GA......C........G.....A...T....CA....G.GATCG.AC.TT.C..AA..C.....A.....AA.G
D. erecta        ...GA...............G.....A...T..A.CA..A.A.GATCG.AC.TC.C..AA..C.....A..CA.AA.G

D. ananassae     TATTCAAAGTCCGGTCCAGACACCTGGCGGAGGCATCTCGTGGAGGCGCTTTGCATTATCGGTGCCCGGCAAGTGATC
D. simulans      .TCG.C......C.G.....A..T...A.A..A.............C..G.....C..T..G.....CA.G...C..
D. sechellia     .TCG.C......C.G.....A..T...A.A..A.............C..G.....C..T..G.....CA.G...C..
D. melanogaster  .TCG.C......C.G.....A......A.A..A.............C..G........T..G.....CA.G...C..
D. yakuba        ..CG.C..T...C.G.....A..A...A.A..A...T.........T..G.....C..T..G..G..AA.G...C..
D. erecta        ..CG.C..A...C.A.....A......A.A..A..........A..T..G.....CG.T..A.....AA.G...C..

D. ananassae     CGCCGTTTGGGCCTCCGCTGGTCGGAGCTCCGTCTGCACTATCTGCCCCACATCGGTGGCCTGACCCTGCACATACAC
D. simulans      ...A.AC....TT..T.....CA......T..AA.......CT.A..G..T....C...GA.C..G.....TG.T..T
D. sechellia     ...A.AC....TT..T.....CA......T..AA.......CT....G..T....C...GA.C..G.....TG.T..T
D. melanogaster  ..GA.AC....TT..T.....CA......G..AA.......CT....G..T....C...GA.C..G.....TG.C..T
D. yakuba        ...A.AC.....T..T.T...CA......T..AA.......CT....G..T....CC..GA.CG.G.....TG....T
D. erecta        ..TAAAC....TT..T.T...CA......AAAAA.T.....CT....G..TG...C...GA.C..G.....TG.G..T
```

```
D. ananassae    CCGCTGCTGAAGAGTCTCTACACGATTTGCGAGCAAATGACGGTGGCCCAGAGCGGTCGGCTAGTCCTGGACGTCGGC
D. simulans     ..T...T..............G...G..T...G.GT..T..T....G.....T.....CT.GT....A..T..T...
D. sechellia    ..T...T..............G...G..T...G.GT..T..T....G.....T.....CT.GT....A..T..T...
D. melanogaster ..T...T.......C.......G...G..T...G.GT..T..T...TG.....T..C..CT.GC....A..T..TC..
D. yakuba       ..C...T.......C.......G...G..T...G.GC..T..T....G.....T.....C..G...T....T..T...
D. erecta       ..A...T.......C..T....G...G..T...G.GT..T.......G.....T.....CT.G.....A..T..G...

D. ananassae    GAGAAGGTGGCGCGACAGCAGGAGGGCGCTGGCGATCCTATGCACTTCAATGATCCCGCCTACCTGGAAATATATCTG
D. simulans     ..A.........A.C.....------..A..A..C..AC.T.G....T.C........G........G..C.T.T..
D. sechellia    ..A.........A.C.....------..A..A..C..AC.T.G....T.C........G........G..C.T.T..
D. melanogaster ..A.......A.A.C.....------..A..A.....AC.T.G....T.C........G........G..C.T.T..
D. yakuba       ..T...........C.....------..A..A.....CC.T.G....T.C......A.G........G..C.T.T..
D. erecta       ..A...........C.....------..A..A.....AC.T.G....T.C......ATG........G..C.T.T..

D. ananassae    TTGGACTGGCTGACGCGGAAAGCGATCAAGCTGGGCGACATCAATACCAACGGCAGCGATGTCCAGCTGCTGATCGAG
D. simulans     C....T........CAAA.G.AGC..A..A-----------------------------------------------
D. sechellia    C....T........CAAA.GGAGC..A...T....G.....A...G.AGCA......A....TG.C......G.....
D. melanogaster C.............CA.A.GGAGC..A...T.A..G.....A...G..GCA...........T.........G...GC
D. yakuba       C.............CA.A.G.TGC..AC..T..........A...G...CG.......................T...
D. erecta       C.............CA.A.G.TGC..AC.......G.....A...GT..TA......A....T...........T...

D. ananassae    CACCTGAAGTTCAATGATCTGCAGGAGCAGGCCAAACTCCTGATCGACACGATCAACAGCAATGCCCGGGATGAGCCG
D. simulans     ------------------------------------------------------------------------------
D. sechellia    ..TT.A........C........T.C...A.....GT.GA.C.CA.....C....TT..T..C..T.C.---.....A
D. melanogaster ..TT......C...C.G........C...A..A..TT.G..C.AA.....C....TT..T..C..T.C.---.....A
D. yakuba       ..TT..........C...T..G...CA........GT.G..C..A..G..T...CTT.....C..T.C.---.....A
D. erecta       ..TT.......T..C...T.AA.............GT.G..C..A.....A....TT..T..C..T.CA---.....A

D. ananassae    GCAACCTCAAGGACCGCCAGCCCACCACGGACG------------ACCGTGAAGCAGGAAACCCTGACGGACAGTCAG
D. simulans     ------------------------------------------------------------------------------
D. sechellia    .ATG.GG.TG.C..T..TGCG---------.T.------------G.AA.C.....A..G.T.GAAT......AC...
D. melanogaster .ATG.GG.TG.C..T..TGCG---------.T.------------G.A..C.....A..G.TTGAAT....T.AC...
D. yakuba       .ATG.GG.TG...T...GGCG---------.T.GCTACGACGGCT..GA.C.....A..G...GACT......AC.G.
D. erecta       .ATTTGG.TGA..TT.ATGCG---------.T.------------..AA.C..A..A..G...GAAT......AC...

D. ananassae    CGGAAT------------------------------TCCGCATCCGCATCCACCTTCGTGCCACGAAATGGCGTTCAG
D. simulans     ------------------------------------------------------------------------------
D. sechellia    .A.TCG------------------------------AAG..TC.T..TATA.AACGAA.A------GG..CTC..A..
D. melanogaster .A.TCG-------------------------------.A.TGT...A..------CAAA.A------G...CTC..A..
D. yakuba       .A.TCGTCCAGCTGCTCCACAGCCGCAGCGGCCTCGAA.....TT..TATGCG.CGAA.C------....CGC..AG.
D. erecta       .A.TCGTCCAGCTGTTCCACAGCCGCAGCGCCTTCGAAA.....T..T.TGTG.CGAA.A------....CTC..AG.

D. ananassae    CTGAGTCGGGACAACGCCGGCATCTGTCTAATCATTAATCAAAGGAAATTCCACCGTAATGTTGACGATAATCTTAAG
D. simulans     ------------------------------------------------------------------------------
D. sechellia    T...CC.....G.....A..A...GCCT.G..T..C..C..GCA.G.G..T.....G......AG.A.GG..A.....
D. melanogaster T...CC.....G.....A..A...GCCT.G..T..C..C..GCA...G..T.....G......AG.AGGG..AA..T.
D. yakuba       T...CC.....G.....A..A...GCCT.G.....C..C..GCA.G.G..T.....G...------------------
D. erecta       T...C......G.....A..A...GCCT.......C..C..GCA.G.G..T.....G...------------------

D. ananassae    AAATACCTATCTCCCAAGCCACTAGCCCAACGCCTGGGCACGGATGTGGACGAGCAGTCGCTGAGAAAGGTCTTCTCC
D. simulans     ------------------------------------------------------------------------------
D. sechellia    ...CTT..G..G...G.C.....GCATAG....GAT..............TA..G.ACGA..A.TCG....G..T...
D. melanogaster ....TT..G..G...G.C.....GCGTAG...GGAT..............TA.AG.ACGA..A.TCG....G..T...
D. yakuba       ....TT..G..A....GA..C..GCAGA.....AAT..............TA..A.ACGA..C.A.G....G..T...
D. erecta       ....TT..G..A........T..GAAAAT....A.T..............TA..A.ACGA..A.CCG....A..T...

D. ananassae    GCGATGGGCTATAAGGTGGAGTCGCACCACAACATCGATCACATGGAAATGGTCCATCTAATGCGCAGTGCCACCGAG
D. simulans     ------------------------------------------------------------------------------
D. sechellia    T.......A..C..T......G..T..G.....G.G.....TC...GC..CA.AG.G.GC..T........GTG....
D. melanogaster T.......A..C..T......G..T..G.....G.G..........GC..CA.AG.G.GC..T........GTG...T
D. yakuba       CT......A..C..T......A.CT..G....TG.G..C...C....C..C..G..G.AC..T..G.....GTG...C
D. erecta       T.......A..C..T......G.AT..G....TG.G..C...C....C..C..G..G.GC..T..G.....GTG...T

D. ananassae    CGATCCCTGCTCAACGATTCGATTGTGGTGTGTATCCTAAGTCACGGCTTCGAGGGAGCTGTCTACGGAGCGAATAGC
D. simulans     ------------------------------------------------------------------------------
D. sechellia    A....G..C..GCG......CC.A.....C.TC.....G..C.............AG..C...............C...
D. melanogaster A....G..CG.GCG......CC.A.....C.TC.....G..C.............AG..C........C.T.C..C...
D. yakuba       .....G..C..GCG......CC.A.....C..C.....G..C.............AG..C........G.....C...
D. erecta       A....G..T..GCGG.....CC.A.....C..C.....G..C.............AG..C........G.....C...

D. ananassae    ATCGCCCTGAGTATCCCAGAGATCGAGAATGTCCTGTGCAGCGAAGCGAACGTGTACGACAAGCACAAGCTGCTGATC
D. simulans     ------------------------------------------------------------------------------
D. sechellia    ..........AG...T.G..T.........CA.G..C......T.C.G..C.C...C.A....A.C....T....A...
D. melanogaster ......A...AG...A.G..T......G.CT.G..C......T.C.AC.C.C.....T....A.CG...T....A...
D. yakuba       ..........AG...A.G..C.........C..G..C..T...T.C.AA.CGC.....A......C....T.....G.G
D. erecta       ..........AG...A.G..C.........C..G..C......T.C.AA.CGC.....A......C.........AG.G

D. ananassae    ATACAGGCGTGCCAGGAC------AACAACCGGAGGCAGCAAGGAATG---------CCGTTCAAGTTAGATGCCACC
D. simulans     ------------------------------------------------------------------------------
D. sechellia    .....A..C.........GAAGCCTGC.C.GAAA.A.G.....AATGA.----------.C..T..TC....C.TG...
D. melanogaster .....A..C.........GAAGCTTGT.C.TAAA.A.A...C.AATGA.----------.TC..T.G.A....C.TG...
D. yakuba       ........C..T.....GAAGCCTG..C.GGACGA.G.......CCA.CGAGGATTG..C......A.C..C.TG...
D. erecta       ........C..T.....GAAGCCTG..C.GAAAGA.G.....AACCA.CTTGGATTG..C..T...A.C..C.TG...

D. ananassae    ACGAGTGAGCCGGGCCAGCATATGCATATGGTGCGAGTGATGCCA---GTATCCGGATTCCCAGCCCTGCGACACACC
D. simulans     ------------------------------------------------------------------------------
D. sechellia    ..CC.GTC............C..CA.C...C....G.CC...T.CACG..GAAT....ATG.T..T.....C......
D. melanogaster ..CGTGTC.....A......C..CG.C...C....G.CC...T.CACG...AAT....ATG.T..T.....C......
D. yakuba       ..CCAGTC.........A..C..CA.C........G.CC...T.CACG..GAAT.....T..C..T............
D. erecta       ..CCAGTC....A....A..C..CA.CT.......G.CC...T.TACG..GAGT.....T..T..T............

D. ananassae    CATACGGGCAGCTGGTTCATCCAGTGCCTGTGCGAGGCTCTAGTTCAACATTCCGACNNNNNNNNNNNNNNNNNNNNN
D. simulans     ------------------------------------------------------------------------------
D. sechellia    ..A.AA...............GG.A..........T..CA.C.A.TCC......TC.AGTGAACACATAGCCGATATC
D. melanogaster ..A..A..T........T...GG.A..........T..CA.C.A..GC.G.....C.AGTGAACACATCGCCGATATC
```

```
D. yakuba       ..C.T...................A..........T..CA.C.AATGC.....G.C.AGTGAACACATCGCCGACATC
D. erecta       ....T...................A.......T..T..CA...AATGC.....T.C.AGTGAACACATCGCCGATATC

D. ananassae    CTGACCATCGTCACCCATGAAGTGGCGAAGAAACGTGGCGACAACAACGAATCCATGCTCCTTAGCTCCAACATATGC
D. simulans     ------------------------------------------------------------------------------
D. sechellia    .....G..T.....AA.C..G...T.T..A..G..A...AGT...G....G......G.G.CG.ATGTT..A.GCACA
D. melanogaster .....G..T......A.C......T.T..A..G..A...AGT..TG....G......G.G.CG.ATGTT..A.GCACA
D. yakuba       .....G..T......A.C......T.CG.A..G..A...C.G...G....G......G.G.CA.A.GTG..G.GCATA
D. erecta       ....TG..T......A.C......T.CG.A..G..A...C.A...G....G......G.G.CA.ATGTTC.A.GCATA

D. ananassae    TTGGTGCAGAACTTCTACCTGCCGCCAAGGATCTCACCATCG
D. simulans     ------------------------.ACCATCAT---------
D. sechellia    ..TCGC...C..G.G...T.C..A..TC.CCAG---------
D. melanogaster ..TCGC...C..G.G...T.T..A..TC.TC.G---------
D. yakuba       ..CCGC...C..G.....T.C..A..TC.TC.G---------
D. erecta       ..CCGC...C..G.....T.T..A..TC.TC.G---------
```

5.MyD88

```
D. ananassae    ATGCGCCCTCGATTTGTATGCCATCAGCAGCACTCGGTTGCC------------------------------CATTCC
D. melanogaster ......................................G...CATTCCCACTAC------------CAGCCC..C...
D. sechellia    ......................................G...CATTCCCACTAC------------CAGCCG..C...
D. simulans     ......................................G...CATTCCCACTAC------------CAGCCG..C...
D. erecta       ..........................A...........G...CATTCCCACTTTGAGCCCCACGTCCAGCCC..C...
D. yakuba       ......................................G...------------------------------..A...

D. ananassae    CATACCCATTCCTCCTCCGCTGTCCACTACCACAGTACCCATTCCCATCCTCGATCTCATCCCCATTACCAC------
D. melanogaster ..CTT...----------------..-------CA......CCG......C---AA..CG......C....TCACATT
D. sechellia    ..CTT...----------------..-------CA......CCG......C---AA..CG......C....TCACATT
D. simulans     ..CTT...----------------..-------CA......CCG......C---AA..CG......C....TCACATT
D. erecta       ..CTT...----------------...C.G...CA.CA...CCG......C---AA..CG.....CC....TCACATT
D. yakuba       .C.TT...----------------...CGTT..CA.TA...CCG......C---AA..CG......C....TCACATT

D. ananassae    ------GCCACTGACGTCAGTCATCGACGTTATCGCTCCTCCGCGAACATGGTGGCCGTGGATGGGATCGGCGGCGTC
D. melanogaster TACGGC...............T....G..........A.---..TGG........TG.CC..G..AG.TATG.A.TC.
D. sechellia    TACGGC...........C...T....G..........A.---..TGG........TG.CC..G..AG.TATG.A.TC.
D. simulans     TACGGC...............T....G..........A.---..TGG........TG.CC..G..AG.TATG.A.TC.
D. erecta       TACGGC...............T....G..........A.---..T.G........TG.CC..G..AG.TATG...TC.
D. yakuba       TACGGC...............T....G..........A.---..T.G........TG.CC..G..AG.TATG.A.A..

D. ananassae    GGCGGTATGGGGGGCTCCCTTGATTCCGGTACCGGAATGGGCCACTTCAACGAGACGCCGCTATCAGAACTAAGCGTA
D. melanogaster ..GTCGGGATC....A.G---.GAA.G------..CT....G..............C..AT....C.C...GG..A.C
D. sechellia    ..GTCGGGATC....A.G---.GAA.G------..CT....G..............C..AT....C.C...GG..A.C
D. simulans     ..GTCGGGATC....A.G---.GAA.G------..CT....G..............C..AT....C.C...GG..A.C
D. erecta       ..GTCGGGATC....A.G---.GCA.G..AT.G..CT....GA.............C...T....C.C...GG..A.C
D. yakuba       ..GTCGGGATC...A..G---.GCA.G..A..G..CT....G...........T..C..AT....C.C...GG.AA.C

D. ananassae    GAGACCCGCACCCAGCTATCCCGCATGCTCAATCGCAAGAAGGTCCTGCGCTCCGAGGAGGGCTACCAGCGGGACTGG
D. melanogaster .................G...........G..C...........G.....A...........................
D. sechellia    .................G...........G..C.................A...........................
D. simulans     .................G...........G..C...........G.....A...........................
D. erecta       ..........A......G...........G..C...........G.....T...........................
D. yakuba       .................G...........G..C...........T.....A...........................

D. ananassae    CGCGGCATCTCGGAGCTGGCGAAGCAGAAGGGTTTCGTCGATGAGAATGCCAATAATCCCATGGATCTGGTGCTGATT
D. melanogaster ....................C...........A........C.....C.....C.......................C
D. sechellia    .................C..C...........A........C.....C.....C.....A.................C
D. simulans     ....................C...........A........C.....C.....C.......................C
D. erecta       ..G.................C...........A........C.....C.....C.......................C
D. yakuba       ....................C...........C........C.....C.....C.....A.................C

D. ananassae    AGTTGGAGTCAGAGGAGTCCACAGACCGCCAAAGTTGGGCACTTGGAGAACTTCCTCGGTATCATCGATCGCTGGGAC
D. melanogaster ..C.....C...C.......C...........G..G..C..TC....AC.......G..C..T........G......
D. sechellia    ..C.....C...C.......G........T..G..G..C...C....AC.......G..C...........G......
D. simulans     ..C.....C...C.......G..A.....T..G..G..C...C....AC.......G..C...........G......
D. erecta       ..C.....C...C.......C...........G..G..C..TC....AC..........C...........G......
D. yakuba       ..C....CA...C....C..C.....T.....G..G..C..TC.A..AC....T..A..C...........G......

D. ananassae    GTATGCGATGATATCCAGGAGAACCTAGCGAAGGACACCGAGCGGTATCACGTGAAACGAGAGCAGCGACAGGCGGCG
D. melanogaster ..C.....C..............T..G..C.........C....C.TCAT.A......AG............A.T..T
D. sechellia    ..C.....C..............T..G..C.........C....C.TCAT.A......AG............A.C..T
D. simulans     ..C.....C..............T..G..C.........C....C.TCAT.A......AG............A.C..T
D. erecta       ..C.....C..............T..G..C.........C.A..C.TCAT.A.A....AG............A.C..T
D. yakuba       ..G.....C..C...........T..G..C.........C....C..CAT.A......AG........G...A.C..T

D. ananassae    CTGGTGGACGCCTGTCCGCCGCCCCCCTCCGACTGCCTGGAGACCAACAAC------AACTACAGC------------
D. melanogaster ..C.....G..G...............AG.......T.C............------.........AGC------AGC
D. sechellia    ........G..G...............AG.......T.C............AACAAC.........AGC------AGC
D. simulans     ........G..G........A......AG.......T.C............------.........AGC------AGC
D. erecta       ..A.....G..G...............AG.........C............------.........AGC------AGC
D. yakuba       ........G..G...........T...AG.....T.T.C............------.........AGCAGCAACAGC

D. ananassae    AAC---AGCATTACAGTGGGCCAGAGCGTACAGATCCTGAGCGACGAGGACCAGCGGTGTGTCCAAATGGGGAAGCCC
D. melanogaster ...---.A...C...........A..T..G........C...............A.A.....A..G.....CC.A..G
D. sechellia    ...---.A...C..G........A..T..G........C...............A.A.....G..G.....CC....G
D. simulans     ...---.A...C..G........A..T..G........C...............A.A.....G..G.....CC....G
D. erecta       .G.AAA.A...C..G........A..T..G........C...............A.A.....G..G.....CC.A..G
D. yakuba       ...---.A...C..G.....T..A..T..G........C........A......A.A.....G..G.....CC.A..G

D. ananassae    CTGCCCCGGTACAATGCCTGTGTCCTGTACGCCGAGGCGGACATCGACCATGCCACAGAGATAATGAATAATCTAGAA
D. melanogaster ......A.A.....C..A.....T........G.....A........T........C.....C..............G
D. sechellia    ......A.A.....C........T........G.....A........T........C.....C..............G
D. simulans     ......A.A.....C........T........G.....A........T........C.....C..............G
D. erecta       ......A.A.....C........T........G.....A........T........C.....C..............G
D. yakuba       ......A.A.....C........TT.......G.....A........T.....T..C.....T.....C........G
```

```
D. ananassae    TCCGAGAGATACAATCTCAGGCTCTTCCTGCGACATCGCGATATGCTGATGGGCGTCCCCTTCGAGCACGTCCAGCTC
D. melanogaster ..T...C................A........C..C.....C.....A........T..G........T........A
D. sechellia    ..T...C................T........C..C.....C.....A........T..G........T........G
D. simulans     ..T...C................T........C..C.....C.....A........T..G........T........G
D. erecta       ..T...C................T........C..C.....C.....A........T..G........T.....A..G
D. yakuba       ..T...C.G..............T........C..C.....C.....A........T..G........T.....A..G

D. ananassae    TCCCACTTCATGGCCACCCGCTGCAACCACCTGATCGTTGTGCTCACGGAGGAGTTTCTCCGGAGTCCGGAGAACACG
D. melanogaster .....T....................T...........C........A...........T..................
D. sechellia    ..........................T...........C........T...........G..................
D. simulans     ..........................T...........C........T...........G..................
D. erecta       ..............T...........T...........C........A...........T..................
D. yakuba       ..............T...........T...T....T..CT.......A...........T.....C............

D. ananassae    TACCTCGTGAACTTCACCCAGAAGATACAGATTGAAAATCACACTCGCAAAATCATACCGATCCTGTACAACCCAGGC
D. melanogaster ....................A.....C........G..............G...........T........AA.G.A.
D. sechellia    ................................C..G......T.......G...........T..C.....GA.G.A.
D. simulans     ....................A...........C..G..............G...........T........GA.G.A.
D. erecta       .............................A..C..G..............G...........T........GA.G.A.
D. yakuba       .............................A..C..G..............G...........T........GATGAA.

D. ananassae    ATGCACATACCACAGACCCTGGGCATCTACACACACATCAAGTATGCCGGTGACTCCAAGCTTTTCAACTTCTGGGAT
D. melanogaster ...........C..............T.....G...........C.....G.........T.G..............C
D. sechellia    ...........C..............T.....G...........C.....G.........T.G..............C
D. simulans     ...........C..............T.....G...........C.....G.........T.G..............C
D. erecta       ...........C..............T.....G...........C.....G.........T.G..T...........C
D. yakuba       ...........C..............T...........G..T..C....CG..T......T.G..............C

D. ananassae    AAACTGGCCCGATCCCTGCATGATCTGGATGCGTTTTCGATTTATTCAACGCGTCAGGTGCAAACACCATCGCCC---
D. melanogaster ..GT....T..T.....A..C...........C........C..C..C.....C........G..T..C.....GGTG
D. sechellia    ..GT....G.....G..A..C...........C........C..C..C.....C........G..T..C.....GGTG
D. simulans     ..GT....G..T........C...........C........C..C..C.....C........G..T..C.....GGTG
D. erecta       ..GT....G..T..G..A..C...........C.A......C..C..C.....C....................G---
D. yakuba       ..GT....G..T..G..AT.C...G..A....C........C..C..CC....C..............C.....G---

D. ananassae    TTGGAGGAAACAGCTCCCCAGGGAGTCCCCACACCCAGCATTCGGATACAGATCAACGACAAAGACATCACCGACATG
D. melanogaster ---......T.G.......G.---..GA....T..............T..............G..TG....T..T...
D. sechellia    ---......T.G..G....G.---..GA....T.............................G..TG....T..T...
D. simulans     ---..A...T.T..G....G.---..GA....T.............................G..TG....T..T...
D. erecta       ---........GT....A.GA---..GA....T...........C.................G..TG....T..T...
D. yakuba       ---......C.GT......G.---..GA....T.................A...........G..TG....T..T...

D. ananassae    CCCAACTATAACAATTGCAGTTCCGCCTCCGAGGCGGATACCACCATGGTATCCATTTCGGGAGACACCAGTTCCCCC
D. melanogaster ..A.....C....GC....AA---.TGC.G........G.....A..C..T..GG....C..T......G.C..A...
D. sechellia    ..A.....C....GC....AA---..GC.G........G.....G..C..T..GG....C..T......G.C......
D. simulans     ..A.....C....GC....AA---..GC.G........G.....G..C..T..GG....C..T......G.C......
D. erecta       ..A.....C....GC....AA---.TGC.G........G.....G..T..T..GG....C..T..T..TG.G......
D. yakuba       ..A.....C....GC....AA---.TGC.G..A.....G.....AG.T..T..G.....C..T..T...G.C..A...

D. ananassae    CTGCCGGAGCACAAGCCGAAGAAGAAGGACAAGTTCCTGCGAAAGTTCACCCTGAACTTTGGCAAGTCGCCAAAGAAT
D. melanogaster .......................A.....TCGC........C.GAA....G.AC.G......A...A..G.GCG.---
D. sechellia    .......................A......CGC........C.GAA....G.AC.G...C..A...A..G.GCG.---
D. simulans     .......................A.....TCGC........C.G.A....G.AC.GT.....A...A..G.GCG.---
D. erecta       ..............A........A.....TCGA........C..AA....A.AC.G...C..A...A..G.GCG.---
D. yakuba       ..............A........A.T.A.T.CC...---..C..AA....G.AC.T.A.C..A...A..G.GCGA---

D. ananassae    ACCAGTAGCACTTCAAGTGGCAAGCCCCTGCGGCACGCCCACTCCGTCAGCACGATCCATGTCACGGAAAGGGAGAGG
D. melanogaster ---..CGATGGGG.C..C......A.T.....T..T.................C..AA...........C.C......
D. sechellia    ---..CGATGGAG.C..C......A.......T..T.................C..AA..........GC.A......
D. simulans     ---...GATGGGG.C..C.....AA.A..A..T..T.................C..AA..........GC.C......
D. erecta       ---..CGATGGGG.C..C.C....A.......C..T..G..............C..AA.C.........C.C......
D. yakuba       ---.A.GATGGGG.C..C.C....A.......T..T..T..............C..AA.C....T...GC.C..A...

D. ananassae    ACACTAAGTGCCAGCAGTTCGAACATCTCAACGACATCGGAGAGCAGGAAG------------TGGCAGCCGAATATC
D. melanogaster .....C...........C..C........C..C..G..........A....AGCTTCATCAAG...........C...
D. sechellia    .....C...........C..C........C..C..G........T.A...AAGCTTCATCAAG...........C...
D. simulans     .....C...........C..C........C..C..G........T.A...AAGCTTCATCAAG...........C...
D. erecta       ..C..C...........C..C..T.....C..C..G..........A....AGTTTCATCAAG...........C..T
D. yakuba       ..CA.C...........C..C..T.....T..C..G..........A....AGTTTTATCAAG....G......C...

D. ananassae    CTCAAGAAGGTCCTGTTCTCGCGATCCAGCAGCAAGCTGCAGACTCCGGGT
D. melanogaster ..G.......C...A..............................G.....C
D. sechellia    ..........C............C....................G.....C
D. simulans     ..........C............C....................G.....C
D. erecta       ..........C...C.....A..C.......A............G......
D. yakuba       ..G......TC.........C..........A......A.....G...T.C
```

6.Pll

```
D. ananassae    ATGAGTGGAGCAGAAACAAGAGACGCCGTC------------AACCACGAAAATGTGAACAGGACTAGATCAAGATCA
D. melanogaster ........C.TCC.G..CGCC..A....AGGCGCAGGCCCAA.....A.CG....GC........C..G..GC.T..C
D. sechellia    ........C.TCC.G..CGCC..A..T.AGGCGCAGGCCCAA.....A.CG....GC........C..G..GC.C..C
D. simulans     ------------------------------------------------------------------------------
D. erecta       ........C---------GCC..GA...AGGCACAGGGCCAA.....A.CG....GC........C..G..GC.T..C
D. yakuba       .......AC.TTC.G..CGCC..A....AGGCACAGGCCCAA.....A.CG....GC........C..G..GC....C

D. ananassae    TATCTGGATGATTCGATTCCTATCCGGCTACTTCCGCTGTCAGTGCGTCATCAGCTATGTGCCCACCTGGATGCACTG
D. melanogaster C.CT....CA.CA.A..GG.C.....A..G..G...T..C.C.....AGCC...............T.......C..A
D. sechellia    C.CT....CA.CA.A..GG.C.....A..G..G...T..C.C.....AGCC...............T.......C...
D. simulans     ------------------------------------------------------------------------------
D. erecta       C.CT....CA.CA....GT.C.....A..G..G......C.C.....AGCC..A..........TT.....C..C...
D. yakuba       C.CT....CA.CA....GT.C.....A..G..G..A...A.C..A..CTCC..A..T.....T..TT...........

D. ananassae    GATGTGTGGCAAGTGATGGCTACAGCGGCGAAACTATACCCGTCCCAAGTGGATCAGATACACAGTGAGAAGCAGAGG
D. melanogaster ..C..A......CA.C....C..G..C.TA.....C..T..CGA...G.....G......AG...CC........C.T
```

```
D. sechellia     ..C........GCA.C....C..G..C.TT.....C..T..CGA...G.....G......AG...CC....A...C.C
D. simulans      ------------------------------------------------------------------C....A...C.C
D. erecta        ..C.........CAA.....C..G....TA.....C..T..AGAT........G......AG....C....A..AC.C
D. yakuba        ............CAA.....C..G..C.TA.....C..T...GA...G.....G......AT....C....A...C.C

D. ananassae     GGGCGCTCGGCGTCCAACGAGTTTCTCAATATCTGGGGTGGCCAGTACAATCACACGGTCCTAACTCTGTTTGCATTG
D. melanogaster  ..C.....A..C.....T.....C.....C..T.....C..T.................G.A...AT.......T...
D. sechellia     ..C.....A..C.....T...........C..T.....A.................A..G.A....T.......T...
D. simulans      ..C.....A..C.....T...........C..T.....A....................G.A....T.......T...
D. erecta        ..C.....A..C.....T.....C.....C..T.....C....................G.A....T.......T...
D. yakuba        ..C.....A..CG....T...........C..T.....C..................A.G.A....T.A.....T...

D. ananassae     TTTAAAAAGTTGAAACTCCACAATGCCATGCGGGTGATTAAGGACTACGTTAGCGAGGACCTACACAAATACATTCCA
D. melanogaster  ..C.....A.....G..T..T..C........TC....C..A..T..............T..G.....G.....A..G
D. sechellia     ..C.....A.....G..G..............TC....C..A.................T..G.....G.....A..C
D. simulans      ..C...........G..G..............TC....C..A.................T..G.....G.....A..C
D. erecta        ..C.....A..A..G........C........AC....C..A.................T..G.....G........C
D. yakuba        ..C.....A.....G..G.....C.........C....C..A.................T..G.....G........C

D. ananassae     CAAAGCGTGCCCACAATAAGCGAACTGCGTGCTCCTCCCGATTCCAGTGTCAGGATAAACAATGGCCCGCCCTATCCC
D. melanogaster  AGG...........C..C.....G.....C...G...............C..AAG.......C........G.T....
D. sechellia     AGG...........C..C.....G.....C...G...............C..AAG.GG.............G.T....
D. simulans      AGG...........C..C.....G.....C...G...............C..AAG.GG.............G.T....
D. erecta        AGG...........C..C..T..G.....C...G...............C..A.G.G..............A.T....
D. yakuba        AGG...........C..C..T..G.........G..................AAT.GG.............A.T....

D. ananassae     TCATCCTCAGGTGTAAGCAACTCCAATAATAACCAAAGCACAACGTCACCAGAAGAT------CCCAGTCTGGAGTCC
D. melanogaster  ..C.....G..C..C........A..C..C..T.GC.C..GC..AA.GG..ACG..GGAGATA.....C.........
D. sechellia     ..C.....G.....C........A..C..C..T.GC.C..GC..AA.GG..ACG..GGAGATT...............
D. simulans      ..C.....G..C..C........A..C..C..T.GC.C..GC..AA.GG..ACG..GGAGATT.....C.........
D. erecta        ..C.....G..C..C........A..C..C..T.GC.C..GC..AG.GG..ACG..GGAGATA.....C......A..
D. yakuba        ..C........C..C........A..C..C..TAGC.C..GC..AG.GG..ACG..AGCGATT..T..CT........

D. ananassae     TTGGGCAACATTCACATCAGCACTGTCCAAAAGGCAGCCGAGTCCCTTATGGAGATTGACTATAACGAGCTGGAAACT
D. melanogaster  C.......T..A.....T.....C..A..GCG.........A...T.GC.......C..T...GCG.....A....AC
D. sechellia     C.......T..A.....T.....G.....G.G.........A...T.GC.......C..T...GCG..........AC
D. simulans      C.......T..A.....T.....G.....G.G.........A...T.GC.......C..T...GCG..........AC
D. erecta        C..........C.....T.....G.....GCG.............T.GC.A.....C..T....CG..........AC
D. yakuba        C..........A.....T.....G.....GCG.............T.GC.......C..T...GCG..........A.

D. ananassae     GGCACCAACAAATGGTGTCCGGAAAATCGCCTGGGACAGGGTGGATTCGGTGAGGTGTATCGCGGACAGTGGAAGCAG
D. melanogaster  .C...GG..GGC...A.......T.....A...........C........A..C.....C.....CA.A........A
D. sechellia     .C...GG..GGC...A.C.....T.....A...........C.....T..C..C.....C.....CA.A........A
D. simulans      .C...GG..GGC...A.C.....T.....A...........C.....T..C..C.....C.....CA.A........A
D. erecta        .C...GG...GG...A.......G.......................T..A..T...........GA.A........A
D. yakuba        .C...GG..GG....A.....C.G.....T..............G..T..A..C.....C.....GAGA........A

D. ananassae     ATGGATGTTGCTATCAAGGTGATGAACTACCGCAGC---CATGTTGACAAGACCCAGGTAGAGCTGCAGCAGAGCTAC
D. melanogaster  C....C..G..C.......................TCCCA.CA.C...C...AAAT...G..................
D. sechellia     C....C..G..C..T....................TCCCA.CA.C...C...AAAT...G..................
D. simulans      C....C..G..C.......................TCCCA.CA.C...C...AAAT...G..................
D. erecta        C....C..G..C.......................TCCCA..A.C...C.A.AGA....G..................
D. yakuba        C....C..G..C........................CCCA.CA.....C...AAA....G..............TC..

D. ananassae     AACGAACTCAAGTATCTCAACTCAATCCGACACGACAATGTAGTGGCCCTTTACGGCTACAGCATCAATGGGGAAAAA
D. melanogaster  ...............T.A...AGC.....G.........A.CC.......C.....A...........A..T.GT..G
D. sechellia     ...............T.A...AGC.....G........CA.CC.......C.....A...........A..T.G...G
D. simulans      ...............T.A...AGC.....G........CA.CC.......C.....A...........A..T.G...G
D. erecta        ........A........A...AGC.....G........CA.CC.......C.....A...........A..T.G...G
D. yakuba        ........T.G......A...AGC.....G..T.....CA.C........C.....A......G....A....C...G

D. ananassae     CCATGCTTAGTATACCAACTGATGAGTGGTGGCTCTTTGGAAAATCGCTTGCGGGCTCACAAATCGGATGCGTCGCTT
D. melanogaster  ..G...C.C..C.....G.......AG..C.....CC....GGC...T..A..A..G..T..GG.AC.AAACC.A..A
D. sechellia     ..G..TC.T..C.....G..A....AG..C.....CC....GGC...TC.A..A.....T..GG.AC.AAACC.A..A
D. simulans      ..G..TC.T..C.....G..A....AG..C..T..CC....GGC...TC.A..A.....T..GG.AC.AAACC.A..A
D. erecta        ..G...C.C..T.....G.......AG..C..T..CC....GGC....C....A..C.....GG.CC.AAATC.A..A
D. yakuba        ......C.G..T.....G.......AG..C..T..CC....GGC...TC.AA.A..G.....GG.CC.AAACC.A.AA

D. ananassae     CCACCATTGACTTGGCGACAAAGGTTCAATATCTGTGATGGCACAGCCAGGGGCATCTACTTCATGCATACGGTCCGC
D. melanogaster  ...G..C.C..C....AG..GC....T.GC...A.CCTC.....G..T..A...........TC....C....CG..A
D. sechellia     ...G..C.C..C....AG..GC....T.GC...A..CT.........T..A............C....C....CG..A
D. simulans      ...G..C.C..C....AG..GC....T.GC...A.CCTC........T..A............C....C....CG..A
D. erecta        ...G.TC.C..C....AG..GC....T.GC...A.CCTC.....G..T..A....T......C....C....CG..A
D. yakuba        ...G.TC.C..C....AG..GC....T.GC...A..CTC.....T..A..A............T....C....CT...

D. ananassae     GGTACGCCGCTCATCCACGGAGACATTAAACCCGCGAACATACTTTTGGACCAGTGCCTGCAGCCCAAGATTGGTGAT
D. melanogaster  ..C..A.....G..T..T.....T.....G..G..C.....C..GC.......A..T........A..A..C..A..C
D. sechellia     .....T.....G.....T........C..G..G..C.....CT.GC.C..T..A..T....T...A..A..C..A..C
D. simulans      .....T.....G.....T........C..G..G..C.....CT.GC.C..T..A..T....T...A..A..C..A..C
D. erecta        ..C..T...........T.....T..C..G..G..C..T..CT.G..A.....A..T..AT...G...........C
D. yakuba        ..CG.T.....G.....T.....T..C..G..G..C.....CT.GC..........TA.......A..A..C..A..C

D. ananassae     TTTGGTCTGGCTCGAGAGGGCCCGAAGTCCATAAATGCCGTGATGCAGGTGAAGAAAGTGTTCGGCACTAGAATCTAC
D. melanogaster  ..C.......TG..C.....T..C......T.GG.C..T...G..G.A.....T.....T..........AG......
D. sechellia     .....G....TG..C.....T..C......T.GG.C..T...G..G.A.....C.....T........C.AG......
D. simulans      .....G....TG..C.....T..C......T.GG.C..T...G..G.A.....C.....T........C.AG......
D. erecta        ..C..G....TG..C.....T..C......T.GG.C..T...G..G.......C.....T..T.....C.AG......
D. yakuba        ..C..C....TG..C.....T..C......T..G.C..T...G..GCA.....C.....T.....T..C.AG.....T

D. ananassae     CTGCCGCCGGAGTTTCGCCACTCAAAAAACCTAAGCACAGGAGTGGATGTATACAGTTTCGGAATAGTTTTACTGGAG
D. melanogaster  .....A........C...A...TC.G.C.A..C.....G..C.....C..C.....C.....C..T..GC.GT.....
D. sechellia     .....A........C...A...TC.G.C.A..C.....G..C.....C..C.....C.....C..T..GC.GT.....
D. simulans      .....A........C...A...TC.G.C.A..C.....G..C.....C..C.....C.....C..T..GC.GT.....
D. erecta        .....A........C...A...TC...C.A..C.....G..T.....C..C.....C.AT..C..T..GC.GT.....
D. yakuba        .....A........C.T.A...TC...C.A..C.....G..CC....C..C.....C..T..C..T..GC.GT....A
```

```
D. ananassae    GTGTTTACGGGCCGCCAAGTGACGGATCGCTTGCCAGAGAATGATCTTCAGCAGGATCTTCTTCACTACGTTAAACAG
D. melanogaster .....C.....T..T..G............G....G..A..C..GACGA..A..A..T.G..GG..........G...
D. sechellia    .....C.....T..T..G...........TG....G..A..C..GACGA..A..A..T.G..GG.......C......
D. simulans     ..A..C.....T..T..G...........TG....G..A..C..GACGA..A..A..T.G..GG.......C......
D. erecta       .....C.....G.....G........C...G.......A..C..AACGA.AA.....T.G..G........C..G...
D. yakuba       .....C.....G.....G............G....G..A..C..AACGA.AA..A..T.G..GG.......C......

D. ananassae    CACTGGCGGCACAATCGCACAGAAGTTCTTGACAAGCACGTCCCAATGCCATTTGGTGCTGACCTGGACATGTGCGTG
D. melanogaster ..G........A..C..G.TG..GC.G..A..G......T.AG..GCA..GA.G..CAAG..G............A..
D. sechellia    ..G........A..C..G.AG..GC.G..G..G......T.GG..GCA..GA.G..CAAG..G............A..
D. simulans     ..G........ANNN---------------------------------------------------------------
D. erecta       ..G........G..C....TG..GC.G..G..A......C.TG..GCA..GA.A..CAA...GT...........A..
D. yakuba       ..T........A..C..G.AG...C.G..A..G......C.TG.TGCA..GA.G..CAAG..G............A..

D. ananassae    TGTGCCATTGAAACAGGACTAGATTGCACTGCCATAGAGCCGCAGAATCGGCCGTTAATGAGTACGGTTCTCAATCGC
D. melanogaster ..C.....C..GG.G..CT.GC.C..T......C.G..T......G....C..A.CC....ACG....G.....G..T
D. sechellia    ..C.....C..GG.G..CT.GC.C..T..A...C.G..T......G.C..T..A.CC...CACG....G.....G..T
D. simulans     ------------------------------------------------------------------------------
D. erecta       ..C.....C..GG.G..CT.GC.A..T......C.G..T......G....A..A.CC...GACG....G.....G..T
D. yakuba       ..C.....C..G..G..C..GC.C..T......C.G..T......G....C..A.C....GA.G....G.....G..T

D. ananassae    TTTGCACCACACCGCATA---AAACCCCAACCAATT
D. melanogaster ..C.A...GTTTGTT.CCGACT.G------------
D. sechellia    ..C.AG...TTTGTT.CC---G.C------------
D. simulans     ------------------------------------
D. erecta       ..C.AT...TTTGTAGCC---G.C------------
D. yakuba       ..C.A....TTTGAA.CC---G..------------
```

7.Rel

```
D. ananassae    ------ATGAATCAATACTATGACCTGGACAAGGGGAAGAATGTGCTGTTTATGAACGATGCCTCCAGCACAAGTGGA
D. melanogaster ATGAAC........G.....C...........T.....A......A................A........C.....C
D. sechellia    ------........G.....C.....A........T..A......A................A........C..C..C
D. simulans     ------..............C.....A........T..A......A................A........C.....C
D. yakuba       ------........G.....C..............T..A.......................A........C.....C
D. erecta       ------........G....TC..............T..A.......A...............A........C.....C

D. ananassae    TACAGCAGCGGCAACTCACCGACCTCAACCACCCAGTCCTTGTCGCCCGCCCACTCCCCGGCCACCATGGCCTTGCAA
D. melanogaster ..T......A.T.CA.....C.A...C....A..GA.....T..A..A............AAA.......AAC.....
D. sechellia    ..T........T.CA..T...CA...C....A...A.....T..A..A.............AA.......AAC.....
D. simulans     ..T........T.CA..T....A...C....A...A.....T..A..A.............AA.......AA......
D. yakuba       ..T.....T..T........C.A...C........A...........A.............AA.........C.....
D. erecta       ..T........T....C..C.A...C........A...........A.............AA.........C.....

D. ananassae    ACGGATTTCGCCAATCTCAACTTGCCAGCCGGGAACTCACCACATCAA---CCGATGCAAAGTTCACCATATCAG---
D. melanogaster .....C...........T..T.....C.G...C..T........C..GCCG..A...GC..AC........C...AAT
D. sechellia    .................T..T.....C.G...C..T..........GCTG..A...GCG.A.........C...CAT
D. simulans     .................T..T.....C.G...C..T........C..GCTG..A...GCG.A.........C...CAT
D. yakuba       ....................T.....C.G...C..T........C...CCG..A...GC..AC........C...CAT
D. erecta       ....................T.....C.G...C..T........C...CCG..A...GCG.AC........C...CAT

D. ananassae    ------CTGTTGAATGGCGGTTTG---ACTCTGGACGCGACTAAT------TTGTTAAATTCCTCT------------
D. melanogaster CAGCTGT..AAC..C.....AA.TTGCCAAT...GT..T..C...------..AA....C...A..GGCGTTAGTTTC
D. sechellia    CAGCTGT..AAC..C.....AA.TTCCCAAT....T..T..C...TTCAAT..AA.G..C...A..GGCGCTGGTGTC
D. simulans     CAGCTGT..AAC..C.....AA.TTGCCAAT....T..T..C...TTCAAT..AA.G..C...A..GGCGCTGGTGTC
D. yakuba       CAGCTGT..AAC..C.A...AA.TTGCCAAT....T..T..C...------..AA.C..C...G..GGCGTTGGTGTC
D. erecta       CAGCTGT..AAC..C.....AA.TTGCCAAT....T..T..C...------..AA.C..C...G..GGCGTTGGTGTC

D. ananassae    GGTGTCCCGAACGCCATGAATTTTGGCAATATGTACAACATGGATCACCAGTACCACATCTCGACATCGGCTGCGGCG
D. melanogaster ..C..TG.T..T.T..CC.G.........C......---...............TTTG.GC.CG.TC....CA.T.T.
D. sechellia    ..C..TG.T..T.TA.CC...........C.C....---...............TTTG.GC.CG.TC.A..CA.T.T.
D. simulans     ..C..TG.T..T.T..CC...........C.C....---...............TTTG.GC.CG.TC.A..CA.A.T.
D. yakuba       ..C..TG.T..T.T..CC.G...C..G..C.....T---...............TTTG.G..CG.TC..A.CA.T.T.
D. erecta       ..C..TG.T..T.T..CC.G.........C.....T---...............TTTG.G..CG.TC.AA.CC.T.TA

D. ananassae    TCGCAAGTTCAACAAAACTTTGGCTACCAGTCGGCGCTTGGCATGCTGCCACCTGGCGATTCAAAACACGTGCCCCAG
D. melanogaster C.A.CGTCC...---........A.....TCAAAT---....C..GCAT.TGAC......ATC...........G...
D. sechellia    C.A.CG.CC..G---........A.....TCAAAT---....C..GCAT.TGGC......ATC...........G...
D. simulans     C.A.CG.CC..G---........A.....TCAAAT---....C..GC.T.TGGC......ATC...........G...
D. yakuba       C.A.CG.CC..G---C....C..A.....TCAAAT---....C..GC.T.TGAC......ATC...........G...
D. erecta       C.A.CG.CC..G---C.......A.....TCAAAT---....C..GCAT.TGG.......ATC...........G...

D. ananassae    CTGAGAATCGTGGAACAGCCAGTGGAGAAGTTCCGCTTTCGGTACAAGAGTGAGATGCACGGCACCCACGGCTCCTTG
D. melanogaster ...C.G.....T..G..A..G.............................C........T..A..A..T..A..GC.A
D. sechellia    ...C.G.....T..G..A..G..............T..............C........T..A..A..T..A..GC.T
D. simulans     ...C.G.....T..G..A..G..............T.......................T..A..A..T..A..GC.T
D. yakuba       ...C.G........G..A..G..............T.....C........C........T..A..A..T..A..GC.T
D. erecta       ...C..........G..A..G..............T.....C..T.....C........T..A..A..T..A..GC.T

D. ananassae    AACGGTGCCAACTCGAAGAGGACCCCGAAAACATTTCCGGAAGTCAGTCTGTACAACTACGATGGTCCCGCCGTAATC
D. melanogaster ..T..C.....T......C....G..C.....C..C.....G..T.CA....G............A........C...
D. sechellia    ..T..C............C....G..C.....C..C.....G....CA....G............A........C...
D. simulans     ..T..C............C....G..C.....C..C.....G....CA....G............A........C...
D. yakuba       ..T..C............C....G.....G..C..C..........CA....CT...........A........C...
D. erecta       ..T..C............C....T..C.....C..C.....G....CA....G............AT.......C...

D. ananassae    CGATGCAGCCTATTCCAGACGAACCTGGACAGCCCCCACTCGCATCAGTTGGTGGTGCGCAAGGATGATCGGGATGTG
D. melanogaster ..G.....TT.G.....A..T...T..........A..T..C......C....C...........C............
D. sechellia    ..G.....TT.G.....A..C...T..........A..T..C......C....C...........C..G.........
D. simulans     ..G.....TT.G.....A..C...T..........A..T..C......C....C...........C..A.........
D. yakuba       ..G.....T..G.....A......T..........A..T..C......C....C...........C..G.........
D. erecta       ..G.....TT.G.....A..C...T..........A..T..C......C....C........................

D. ananassae    TGCGATCCGCACGATCTGCACGTGTCCAAGGAGCGCGGCTATGTGGCTCAGTTCATTAACATGGGAATTATTCACACC
D. melanogaster ...........T...T...............................G..A..T..C........C..C..A......
D. sechellia    ...........T...T...............................A.....T..C........T..C..A......
```

```
D. simulans      ..T........T...T...............................A.....T..C........C..C..A......
D. yakuba        ...........T...T.......C.......................C.....T..C........C..C..A......
D. erecta        ...........T...T...................T...........C.....T..C..T.....C..C..A......

D. ananassae     GCCAAGAAGTACATATTTGATGAATTGTACAAAAAGAAGAAGGATCGGCTGGTCTTCCAAATGAACCGACGCGAGTTG
D. melanogaster  .................C..G...C...G...G......C.......C........T..G........C.........
D. sechellia     .................C..G..GC...G...G......C.......C........T..G........T.........
D. simulans      .................C..G..GC...G...G......C.......C........T..G........T.........
D. yakuba        .................C..G..GC...G...G......C.......C........T..G.....T..C.........
D. erecta        ..........T....AAA..A..GC...T...G......C.......C........T..G.....T..C.........

D. ananassae     TCCACCAAGCAAGTGCAGGAGCTGCACCAGGAGACCGAGCGGGAGGCCAAGGACATGAATCTTAATCAGGTTCGCCTT
D. melanogaster  ...CA...A..GC.A.....A.....T........A.....T.................CT.G..C.....G..G..C
D. sechellia     ........A..GC.A.....A.....T........G.....T.................CT.G..C.....G..G..C
D. simulans      ........A..GC.A.....A.....T........G.....T.................CT.G..C.....G..G..C
D. yakuba        ........A..GA.C.....A.....T........A.....T.................CT.G..C.....G..G..C
D. erecta        ........A..GA.C...........T........G..A..T....G............CT.G..C.....G..G..C

D. ananassae     TGCTTTGAGGCCTTCAAGATCGAGGACAATGGCGCATGGGTGCCACTTGCGCATCCGGTGTACAGCAACGCGATCAAC
D. melanogaster  ..............T..A..T........C...........T........A.CG..T..A........T..A.....T
D. sechellia     ..............T..A..T........C...........T........A.CG..T...........T........T
D. simulans      ..............T..A..T........C...........T........A.CG..T...........T........T
D. yakuba        .....C.....A.....A..A........C...........T........A.CG..T...........T........T
D. erecta        .....C.....A.....A..A........C..............C.....A.CG..T...........T........T

D. ananassae     AACCGCAAGTCGGCCCAAACCGGAGAGCTGCGCATCGTTCGGTTGAGCAAACCAACCGGCGGCGTCATGGGCAACGAC
D. melanogaster  ..............A.....T........T........C..CC..........C..T.....G..G...........T
D. sechellia     ...........A..A.....T.................C..CC.......G..C..T.....G..G...........T
D. simulans      ...........A..A.....T.................C..CC.......G..C..T.....G..G...........T
D. yakuba        ...........A..A.....T.................C..TC..........T..T.....A..G...........T
D. erecta        ...........A..A.....T.................C..CC.......G..C..T.....A..G...........T

D. ananassae     GAGCTCATCCTGCTGGTGGAGAAGGTTAGCAAGAAGAACATTAAGGTGCGCTTCTTCGAGGAGGACGAAGACGGCGAG
D. melanogaster  .....T..T..A........A.....C.................A...A.G..............T..G.....G..A
D. sechellia     .....T..T..A..C.....A.....C..............C........G..T...........T..G.....G..A
D. simulans      .....T..T..A........A.....C..............C........G..T..............G.....G..A
D. yakuba        .....T..A...........A.....C.....A........C........G..............T.....T..A..A
D. erecta        .....T..A..A........A.....C..............C........G..............T..G.....A..A

D. ananassae     ACTGTGTGGGAGGCGTACGCCAAGTTTCGCGAGTCGGATGTCCATCACCAGTATGCGATTGTTTGCCAGACGCCTGCG
D. melanogaster  ..C...........A.....A.....C.....A........A..C.....A.....C.....G............C..
D. sechellia     ..C...........A.....A.....C.....A........A..C.....A.....C.....G...............
D. simulans      ..C...........A.....A.....C.....A........A..C.....A.....C.....G...............
D. yakuba        ..C...........A.....A.....C.....A........A..C.....A.....C.....G...............
D. erecta        ..C...........A.....A.....C.....A........A..C.....A.....C..C..G........A......

D. ananassae     TACAAAGACAAGGACGTGGATCGAGAGGTGGGCGTGTCCATTGAGCTAATACGCCCCTCCGACGATGAACGCTCCTAC
D. melanogaster  ........T...........C..C.....CAA.....A...C.....C..T..T..............G.....A.T.
D. sechellia     ........T...........C..C.....CAA.....A...C.....C..T.................G.....A.T.
D. simulans      ........T...........C..C.....CAA.....A...C.....C..T..T........A.....G.....A.T.
D. yakuba        ..............T........T.....CAA.....AT........C..T..T........T.....G.....A.T.
D. erecta        ........T.....T.....CT.C.....CAA.....AT........C..T..T..............G.....A.T.

D. ananassae     CCGCCCCTGCCCTTCCGCTACAAGCCGCGCGATGCGATCGCTTCGAGAAAGAGGCGACGCACCTGCTCCTCCGCC---
D. melanogaster  ...G.G....................A..GAGC.TA..T.TG.....G..AC.C.....A...G.G.....T...AAT
D. sechellia     ...G.G....................A..G..C.TA..T.TG.....G..AC.C.........G.GA....T...AAT
D. simulans      ...G.G....................A..G..C.TA..T.TG.....G..AC.C......C..G.GA....T...AAT
D. yakuba        ..TG.G....................A..AA.C.T...T.TG..C..G..AC.C..T..A...........G..AAAC
D. erecta        ...G.G....................A..GA.C.T...T.TG..C..G..AC.C..T..T...........G..GAAC

D. ananassae     ACCAGTAGTTCGGGCAACTCCAGTTCCAACAATTCGTTCGATTTCCCGAAGACCATTGCAATGGCTCAGCCA---AAT
D. melanogaster  .G...C........A.CAGAA..C..T..T..C...C.G...C.G..C..A..GC.G.GCT....G......CCA...
D. sechellia     .G...C........G.CAGT...C..T..T..C...C.G...C.G..C..A..GC.ATGCT....G...G..CCA...
D. simulans      .G..TC........A.CAGG...C..T..T..C...C.G...C.G..C..A..GC.G.GCT....G...G..CCA...
D. yakuba        .GT..C.....C..A.CTGG...C.....T..C..TC.G...C.G..C.GA..GC.G.GCT...........CCG...
D. erecta        .G...C........A.CTCT...C.....T..C...C.G...C.G..C..A..GC.G.GCT....G.....TGCA...

D. ananassae     GGAAAGCCCAACTATTCGCAGCACGACAAAACCATCAGTCAGGAGTTTGGACGGGACAATCATCTGGATGATCTGTTG
D. melanogaster  ..CTT...A...CT...A.....T..TC.G..T..A...G.......C.....C..A..G.....AA....AT.TA.T
D. sechellia     ..CTT...A...CT.........T..AC.G..T..A...........C.....C..A.TG.....A..G...T..A.T
D. simulans      ..CTT...A...CT.........T..AC.G..T..A...........C.....C..A.TG.....A..G...T..A.T
D. yakuba        ..CCT...A...CT.........T.GTC.G..G..A...........C.....C....TG......A.....T.AA..
D. erecta        ..CTT.ATA...CT.........T..TC.G..T..A...........C.....C..T.CG............T..A.C

D. ananassae     AACTCTCCGGACTACCGCAAACTCATCTACCAGAATTCAGCCGAGCTGGAGAAAATCTGTCAGTTGGGCGAGTTGGTC
D. melanogaster  GCT..CGAA..T.T.........T...G.G..C..C..TT.T..T..C.....G.....C.....A.A.AT.------
D. sechellia     C.T..CGAAA.T.T...G...T.T...GGGA.C..C..TT.T..TA.C.....GT.G..C.....A.A.AT.------
D. simulans      C.T..CGAAA.T.T.......T.T...GGGA.C..C..TT.T..TA.C.....GT.G..C.....A.A.AT.------
D. yakuba        G.T..CGAAA.T.T.........T...G.G.....C..TA.T..T..C.....G..T..CG.C..A...AT.------
D. erecta        GGT..CGAATCT.T.........T...G.GA.C..CA.TT.T..T..C.....G..G..C...A.A.A.AT.------

D. ananassae     GAGCTGCAGCATGGGGGCGAGCTTCAGAACGACGGCCATGGCCGCTCAATGTCGGACGTGCCT------TCGGCAAGA
D. melanogaster  ---------------..A...T.G...C....T..T...AA...G------G....A.....A------...CATC..
D. sechellia     ---------------..A.T.T.GG..CC...T........T..G------G....ATC.G.A------....GTC..
D. simulans      ---------------..A.T.T.GG..CC...T........T..G------G....ATC.G.ATTGGCA....GTC..
D. yakuba        ---------------..A...T.GGTA..G..T........A..G------G....A...G.A------....GTC..
D. erecta        ---------------..A.T.T.G........T........A..G------G....A...G.A------.....TC..

D. ananassae     AATTTAACCAATTACTACCTGAACGAAATCTTCAAGATCTTCGACGAGGATCGACACGCCAAATCGGAATCGGCTCGT
D. melanogaster  ...CGT..A.T.A.A.G.T..G.T..CT.A...G.A..T.A.A.AC....C..CATAT.GCC.ATAA..ATCT.G.AC
D. sechellia     ...C.T......A.A...T....T..CC.A.....A....A...AC....C..C.GAT.GCC.AT.A..AACT.G.AG
D. simulans      ...C.T......A.A...T....T..CC.A.....A....A...AC....C..C.GAT.GCC.AT.A..AACT.G.AG
D. yakuba        ...C.C....G.A.A...T.....ACC..A.....A........TC....C..C.GAA.GCCGA.....AGCT.A.AC
D. erecta        ...C.T......G.A...T.....A.C..A.....A........TC....C..C.GAA.GCCGAT..T.AACT.G.AC

D. ananassae     CGGCGGGTGGAGGAGCTGTTCACAGATCACGCCCTGAAGAAC---GAGAACAAT---GACACGTTGCTGCACGAGGTT
```

```
D. melanogaster .ACAAA......A..TG...T.TC..G..T..T.....T..------T.....AGA......C.C........A..G
D. sechellia    .AC.AA......A..T.A..T.AC.....T..T.A..T...------..T...---......C.C........A..G
D. simulans     .AC.AA......A..T.A..T.AC.....T..T.A..T...------..T...---......C.C.....G..A..G
D. yakuba       ..C.AA................G.....T...T....T...GAA---..T...---......C.CT.......A..G
D. erecta       .TC.AA......A..T....T.AG..G..T..T.....C...------..T...---......C.CT..........G

D. ananassae    ATTAGCTGCAAGAAGGACAACTTGAAGCTGGCCATTAAGACCATACAGGTTATGAACCATTTCAAGCTCAATGAGCTG
D. melanogaster ..C...CA...A........AC............CC....G........G......T.C.....T..G..A..TG..
D. sechellia    ..C...CAG.........C.C............CC....A........G......T.C.....A..G.GA......
D. simulans     ..C...CAG.........C.C............CC....G........G......T.C.....A..G.GA......
D. yakuba       ......CA...A........TC............C.....G........G......T.C.....C..A..G......
D. erecta       ..C...CA...A.........C......A......C.....G........G......T.C........A..G..C...

D. ananassae    GCCGACAACGCCTTGAATGCGGATGGAGACAGTGCCTTGCATGCGGCCTGCCAACAAGATCGGGCGCACTACATTCGA
D. melanogaster .T.A.T.G.A.T...........C..T..T......C......T...G.....G.....C..A............A...
D. sechellia    .T.A...G...T...........CC.T..T...C..C......T...G.....G.....C..A............A...
D. simulans     .T.A...G...T...........CC.T..T...C..C......T...G.....G.....C..A............A...
D. yakuba       .T.A...G...A..............T..T......C......T...G...........C..A..A...........C
D. erecta       .T.A..GGA.................T..T......C......T...G.....G.....C..A..............C

D. ananassae    CCACTTCTGGGACTGGGCTGCAGTCCCAATCAACAGAACCACGCTGGGAACACGCCACTGCACCTGGCCGTCAAAGAG
D. melanogaster ..TT.G.....TA....A....A...A....TGA....TA.T..C..A..T..C........TG..........G...
D. sechellia    ..TT.G.....TA..........A...A.....GA....TA.T..C..A..T..C........TG..........G...
D. simulans     ..TT.G.....TA..........A...A.....GA....TA.T..C..A.....C........TG..........G...
D. yakuba       ..TT.G.....CA..............A.....G..A..T..T..G..A..T..C........TT....A.....G...
D. erecta       ..TT.G.....TA.A............A.....GA.A..T.TA..G..A.....C..G.....TT....A.....G...

D. ananassae    GAGCACCTGAACTGCGTGGACAGCTTCCTGAACGGAGCTCCCATCAGAGCGGGAGAAATAGAGCTGGACTTGACGATA
D. melanogaster ......T.A.G.........A........T..T....TG..A.C.------------G..C.AT.....C.CT..C..
D. sechellia    ......T.............A........T..T....TG..A.C.------------G.TA.AT.....C.CT..C..
D. simulans     ......T.............A........T..T....TG..A.C.------------G..A.AT.....C.CT..C..
D. yakuba       ......T.............A........T..T....TG..A.C.------------G..C.AT.....C.CT..C.G
D. erecta       ......A....T........A........T..T...ATG..A.C.------------...C.AT.....C.CT..G.G

D. ananassae    AAGAACGATGATGGATTAACGCCCCTGCACATGGCCATCCGGCAAAACAGGTACGATGTGGCCAAGAAGCTGATCAGC
D. melanogaster .C......C.....TC.G.....TT.............T..A..G....A..................A.........
D. sechellia    .C......C.....TC.G.....TT.............T..A..G....A..................A.........
D. simulans     .C......C.....TC.......TT.............T..A..G....A..................A..A......
D. yakuba       ..A.....C.....TC.G.....TT.............A.....G....A..................A.........
D. erecta       .C......C..C..T..G.....TT.............A.....G....A..................AT........

D. ananassae    CACGACAGGAGTTCCATTAGCGTGGCGAACACAAAGGACGGAAATAACGCTCTGCATATGGCAGTCCTGGAGCAGAGC
D. melanogaster T.T..TC...CC..G...........C.......T...T..T.....T..A..C..C........T........A..T
D. sechellia    ..T..TC...CC..G...........C......TT...T..T.....T..A..C..C.....C..T........A..T
D. simulans     ..T..TC...CC..G...........C......TT...T..T.....T..A..C..C.....C..T........A..T
D. yakuba       ..T..TC....C..G...........C.......T...T..T.....T........C.....C..T...........T
D. erecta       ..T..TC....C..G...........C.......T...T..T.....T........C.....C..T........A..T

D. ananassae    ATAGAGTTGCTGGTGCTCATTTTGGATGCCCAGAACCAATCTCTCGCGGACATCCTGCAGGCAACAAATGCCGCTGGA
D. melanogaster G.G...C.AT...........C..........A..TG..AAC..TA.C...............CA......A..C..C
D. sechellia    G.G...C.AT...........C..........A..TG..AAC..TA.C...............CA......A..C..C
D. simulans     G.G...C.AT...........C.......T..A..TG..AAC..TA.C........A......CA......A..C..C
D. yakuba       G.G...C..T...........C..........A..T...AAC..TA.C..........T....CGT.....A..C..T
D. erecta       G.G...C..T...................T..A..T...AAC..TA.C..T............CG......A..C..T

D. ananassae    TATACACCCATACAGTTGGCCAGGTGCAAGGCCAACGAGCGAGTGGTGCAGCTCCTGGAGAAAGTGTATCCGGAGAAG
D. melanogaster C....T...T.GG.A......GAAC.......A..T..C..G.....C.....G...A....T...............
D. sechellia    C....T...T.GG.A......GAACT.........T.....G.....C.....G...AG...G......T........
D. simulans     C....T...C.GG.A......GAACT.........T.....G.....C.....G...AG...G......T........
D. yakuba       .........T..G.A.........CA.........T.....G.....AA....G........T...............
D. erecta       ..C......T..G.A.......A..A......G..TA.C..G...........G........T...............

D. ananassae    AGCGATGTGGCTATGACCTGGATACCGCGGAAGGTGAAAGAGGAGATTGACTCTTCCTCCGATGACAGCAGCGATGCT
D. melanogaster G.A..AC....C...........T..AT.T.....C..G........C..T..A..G..G..C..A..T.........
D. sechellia    G.A..AC....C...........T..AT.T.....C..G........C..T..A..G..G..C..A..T.........
D. simulans     G.A..AC....C...........T..AT.T.....C..G........C..T..A..G..G..C..A..T.........
D. yakuba       G.G..AC....C...........T..A..T.....C...........C..TA.G..G..G..C..A............
D. erecta       G.G..ACA...C...........T..A..T.....C...........C..T..A..G..G..C..A............

D. ananassae    GGGCAGCTAGAAATCAAGGCGGAGGAGATGTACATAAAAACGGAGGATGAGGACTCCGTTGAAATGGATTTGAGCAGT
D. melanogaster ..T.....G..G......T.C.........G....CG....AA....C.....T.....G..GT....CC.A..T...
D. sechellia    ..T.....G..G......T.C.........G....CC....A.....C.....G.....G..GT....CC.A..T...
D. simulans     ..T.....G..G......T.C.........G....CC....A.....C..C..G.....G..GT....CC.C..T...
D. yakuba       ..T.....G..G......T.C.....A...G....C.....A.....C.....G..G..G..GC....CC.A......
D. erecta       ..T.....G.........T.C...C.A...G.......G.T............T..GC.G..GT....CC.A......

D. ananassae    ---CGAGGAAGTCGGGAAACGGAATCAAGTCAAGACGCTGAAAGCGGCACTTCCCAATGGCACAAACTCCAACACCTG
D. melanogaster GGT.C.A....A.A.A..GAT.....C...AG....A.C....TG.A..AC---------A.....T.G..GTTG...
D. sechellia    GGT..TC....AT..A..GAA.....C...AG....A.C....TG.A..AC---------A.....T.G..G.TG...
D. simulans     GGT..TC....AT..A..GA......C...AG....A.C....CG.A..AC---------A.....T.G..G.TG...
D. yakuba       GGT..TC....AAT...GGT...T..C...A.....A.G....AG.AGCAGGA..CGCTAAT....T.G.GGTTG..C
D. erecta       AGT...AA.CAG---...GT...TC.G-----------------------GA.TTGCTAA.....A.GTGGTTT...

D. ananassae    CTCAAGAACCATTCTGTGTACGAGCAGCTAAGTGCTATGCTCGGTGAACCGGTGGGTCATGGTGCCGATGCCCAGGTG
D. melanogaster .....A...A.A.TCA.T.....C.G...CT..T.GC....GAA.C.G..TT....A.....GT.....C....A.AT
D. sechellia    ..T..A...A.A..CA.T.....C..A..CT..T.GC....GAA.C.G..TT....A..A..GT.....C....A.AT
D. simulans     ..T..A...A.A..CA.T.....C.....CT..T.GC....GAA.C.G..TT....A..A..GT.....C....A.AT
D. yakuba       ...TC....G.A.TCA.T.....C.....CT..T.GC....GAA.A.G..CT.A..AT.C...T.....C....A.A.
D. erecta       .....AG..G.A.TCA.T.....C.....CT..T.GC....GAA.A.GT.TT.A------...T.....C....AAA.

D. ananassae    CCCAAATGGAAGCTAATCGCCCGCCAATCCCATCTGGAGAAATTCGCCTTTCTGTGGGCCAGTGCTGATGAACTGTTG
D. melanogaster .GG.......T..A.C.A........GA.G......A..C.G.........A.A...CTGG.C..G..G..T......
D. sechellia    .GG.......TC.A.C.A........GA.G.....A..AC.G...A.A...A.....CTGG.C..G..G..T......
D. simulans     .GG.......TC.A.C.A........GA.G.....A...C.G...A.A...A.....CTGG.C..G..G..T......
D. yakuba       .............ACC.A........G.........C..C.G...A....CA.A...CTGG....G..C.GC......
D. erecta       ..G..........ACC.A...T....G..TG....CA..C.G...A.....T.A...CTGG....G..C..C......
```

```
D. ananassae    GATTATATTCTGGACAATGCCGCTACCGTGGACTTTGGAGCATTCGCCAGCGCCCTGCAAGTGATAGATCCCAAGGCC
D. melanogaster ...C.CG.GAAAAGA..A.GA..CT.T..A..G...TCCA....T..TC.T........G.CAG.G.....AC....T
D. sechellia    ...C.CG..AAAAGA..A.G...CT.T..A..G....CCA....T..TC.T........G.GAG.C......C....T
D. simulans     ...C.CG..AAAAGA..A.G...CT.T..A..G....CCA....T..TC.T........G.CAG.C......C....T
D. yakuba       ...C.CG...GA..A..A.G...C.GT.....G....C.A.C..T.TTC.T........G.CAG.C.....GC....T
D. erecta       A..C.CT..AAA..A..A.G...C.GT.C...G..C.CCA....T.TTG.T........GACAG.C......C....T

D. ananassae    TACGCCCTGCTG------------GACCGGGATTTACGT
D. melanogaster ........A...GTTAACCCAACT---------------
D. sechellia    .....AG.T...GTTAACCCAACT---------------
D. simulans     .....AG.T...GTTAACCCAACT---------------
D. yakuba       ............------------..T..A..C......
D. erecta       ..T..T..A...------------A.T..A..CA.....
```

8.Tab2

```
D. ananassae    ATGGCGGCCACACCACCAATGCCGCCCGGATCAAACTCTGACCCATTGAATGGCAGCCATCCCGAGGCGAAGGTCAAG
D. melanogaster ........T...........................A.CC.T.A..........---------------------..A
D. sechellia    ........T...........................A.CC..A..........---------------------..A
D. simulans     ........T...........................A.CC..A..........---------------------..A
D. yakuba       ........T...........................A.CC..A..........---------------------..A
D. erecta       ........T...........................A.CC..A..........---------------------..A

D. ananassae    GCAATCGGCCAGGGACACAACCACATAACCGAGGCAATCAATCAGCACTTTCAGCGAAAGAATCAAAAC---------
D. melanogaster A..G.....A....G---....C.C..G.T.....G....................G.....A..G..T------CAG
D. sechellia    A..G.....A....C---....C.C....T.....G..........................C..G..TCAGAACCAG
D. simulans     A..G.....A....C---....C.C....T.....G....................G.....C..G..------CAG
D. yakuba       AA.G.....A....C---..T.C.C....T.....G....................G.....C..G..------CAA
D. erecta       ...G.....A....C---....C.C....T.....G........A.......T...G.....C------------CAG

D. ananassae    AATCAGGAGCCA---CCAGCGCAGCAGCAGAACCAAAGCCATCCAGGTAGCCGCAGAGGCAGCAATGAGCACGGGCTG
D. melanogaster C.C...AGT..CAAC..CAGT.CAA.C.C...T.CC.A.AG...C.CC........C..G...T.......G---...
D. sechellia    C.C...AGT.ACAAC..CAGT.CAA.C.C...T.CC.A.AG...C.CC........C..G...T.......G---...
D. simulans     C.C...AGT.ACAAC..CAGT.CAACC.C...T.CT.A.AG...C.CC........C..G...T.......G---...
D. yakuba       ..C...AGT..CAAT..CAGT..TA.C.C...T.CC.ATAG...C.C.........C..G...T.......G---...
D. erecta       ......AGC..CAAC..CAGT..AA.T.C...T.CC.A.AG...C.CC........C..G...T.......G---...

D. ananassae    GAGCTGCTGGAGGACAGTGCA------------GAGCAGTCGGAGTCCAGTGCAGTCCAGGGTGGTGGCACGGCCAAG
D. melanogaster ...........TC..GCAA.GCATCCACAGCAAC.A...GAA.CCAG...----------------....A.......
D. sechellia    ......T....TC..GCAA.GCATCCACAGCAGC.A...GA..CCA....----------------....A.......
D. simulans     ...........TC..GCAA.GCATCCACAGCAGC.A...GA..CCA....----------------....A.......
D. yakuba       ...........TC..GCAA.GCATCCACAGCAG..A...GA..CCA....----------------....A.......
D. erecta       ...........TC..GCAA.GCATCCACAGCAG..A...GAA.CCA....----------------....A.......

D. ananassae    CCATCAAGCTGCCATTGCACCAACATTAGTATTATGCACCTGTTCCATGAGATGAAGCAGGAGTTCCCCACGATACCC
D. melanogaster ..GAGC---...................................T.................................
D. sechellia    ..TAGC---..T................................T...........A....................G
D. simulans     ..TAGC---..T................................T...........A.....................
D. yakuba       ..GAGC---..T................................T.................................
D. erecta       ..GAGC---..T................................T.................................

D. ananassae    GATGCCATAGTGACGCAATGCGTCAATGAGAATTGCCATCAGCGCGACAATTGCATCCAAATGCTGAGGAAGGAGTTC
D. melanogaster ...............................................A..C..T.....G...T...........C..
D. sechellia    ........T......................................A..C..T.....G...T...........C..
D. simulans     ..C.....T......................................A..C..T.....G...T...........C..
D. yakuba       ...............................................A..C..T.....G...T...........C..
D. erecta       ...............................................A..C..T.....G...T...........C.T

D. ananassae    GCCCTGCAGGCC------GTTCAAAGCTATCCCTCCAAGGTGCTGCAGCAGCAGCAGCAGCAGAACCGT------CGG
D. melanogaster ..G..T..TC.TATCCCA..G..G........GG.A..............A...........AC...ACCAGAATA..
D. sechellia    ..G..C..TC..ATCCCA........T.....GG.A..............A........A..AC...ACCAGAATA..
D. simulans     ..G..C..TC..ATCCCA........T.....GG.A..............A...........AC...ACCAGAATA..
D. yakuba       ..G..C..TC..ATCCCA........T.....GG.G......T.......A...........AC...ACCAGAACA..
D. erecta       ..G..C..TC..ATCCCA........T.....GG.G......T.......A...........AC...ACCAGAACA..

D. ananassae    CCCACGAAGCCCCCCACGCCACTGAAACCATTGCGGGCCGCTCCAGTCCAACCGGAAGTAGCCCACAGCAATGGGAAT
D. melanogaster .AGG.C.....T........G..C..G..G.CA...ATA..A...CCG..G.......CT.TG.TG........AGTG
D. sechellia    .AGG.C.....T........G..C..G..G.CA...ATA......CCG..G.......CT.TG.TG........AGTG
D. simulans     .AGG.C.....T........G..C..G..G.CA...ATA......CCG..G.......CT.TG.TG........AGTG
D. yakuba       .AGG.C.....T........G..C.....G.CA....A..A...CCA..T.......C..GG.TG.C......AGTG
D. erecta       .AGG.C.....T..T.....C..T..G..G.CA....A..A...CCA..........C..GG.TA........ATTG

D. ananassae    ACTACAAGT------------AGTCCC------------ACACCCCGACCTGTTAGACCCACCACGCTTAACCTACAG
D. melanogaster G....GCCGCCAGGTGTTGGT..C..ACATCCTGATACGCAG...A.G..G---C.......G.....C..T..G..A
D. sechellia    G....GCCGCCAGGTGTTGGT..C..ACATCCTGATACGCAG...A.G..A---C.......G.....C..T..G..A
D. simulans     G....GCCGCCAGGTGTTGGT..C..ACATCCTGATACGCAG...A.G..A---C.......G.....C..T..G..A
D. yakuba       G.C..GCCGCCAGGTGTTGTT..CT.ACATCCTGATACGCAG...A.G..A---C.......G........T..G..A
D. erecta       G....GCCGCCAGGTGTTGGT..CTTACAGCCTGATACGCAG.....G..A---C.......G........T..G..A

D. ananassae    CGTCCGGGCAATACGCAGCTGCAGATGAAGATCCAGCAGCGCCAGCAGCGGCAGCAGCGGGATCCCCCCCAGCAACAG
D. melanogaster ....A.TTT.GC..T.........CAA.....TAT...............A......A......A..AG.CAGCC...
D. sechellia    ....A.TTT.GC..T.........CAA.....TAT...............T.....TA......A..AGGCAGCC...
D. simulans     ....A.TTT.GC..T.........CAA.....TAT...............T......A......A..AGGCAGCC...
D. yakuba       ....A.TTT.GC............CAA.....T.T...............T......A.....TA..A.GCAGCC...
D. erecta       ....A.TTT.GC..T.........CAA.....T.T...............T......A......A..AGGCTGCC...

D. ananassae    CTGACGCCCACGTCCCTGAGCAAGCCGCTGCGACGAGCCCCGCCCCTGCCG------------CCCAAGCCGGGCAGC
D. melanogaster ...........T...T.............A..C..G..A.....A..T...CCGCCACCCAAA..G.....C......
D. sechellia    ...........T...T................C..G..A.....A..T...CCGCCACCGAAA..G.....C......
D. simulans     ...........T...T................C..G..A.....A..T...CCGCCACCGAAA..G.....C......
D. yakuba       ...........T...T................C..G..A.....A..T...CCGCCACCCAAG..G.....C......
D. erecta       ...........T...T..........A.....C..G..A.....A..T...CCGCCACCCAAA..G.....C..T...

D. ananassae    TTTTCCAACGATTCCTCGTGTCTGACCAGCCCGATGAGCTCCAGTGAATCGGAGCTCTCGCTCAACGCGGTCTCGCTA
D. melanogaster ..C....................C.....T..C...........C...........T...............C.CT..
```

```
D. sechellia    ..C..............A.....C.....T..C...........C..G........T...............C.CT..
D. simulans     ..C..............A.....C.....T..C...........C..G........T...............C.CT..
D. yakuba       ..C..............A.....C.....T..C...........C.....T..............T....C.C.CT..
D. erecta       ..C..A...........A..C..C.....T..C...........C..T........T........T......C.CT..

D. ananassae    TCGTCACCGACGTCG---GCAGCGCCAGCGCCAGCGCCAGCGGCATCGGCCAGAACAGCTGGAACAGCAGCGGGCGCG
D. melanogaster ........A......---..G..----..TA.T..TG.T.....G..A..A------------------------..A
D. sechellia    ........A..A...---..G..----..TA.T..TG.T.....G..A..A------------------------..A
D. simulans     ........A..A...---..G..----..TA.T..TG.T.....G..A..A------------------------..A
D. yakuba       ........A......TCG..G..----..TA.T.GTG.T.....G..A..A------------------------..A
D. erecta       ........A......---..G..----..TA.T.GTG.T.....G..A..A------------------------..A

D. ananassae    ATAGTGCAGCCACAACAACAGCCCTCTCCCGTTCGACATCGTTCAGTCATTACGCTCCAGCCAGAGCCGCCGTACGCG
D. melanogaster ..C.CATCAA.G........A.....G..G..G.................A.....A.....................
D. sechellia    ..C.CATCAA.C........A.....G..G..G.......................A.....................
D. simulans     ..C.CATCAA.G........A.....G..G..G.......................A.....................
D. yakuba       ..C.CATCAA.G........A.....G..G..G........C..............A.....................
D. erecta       ..C.CATCAA.G........A.....G..G..G........C......G.......A................T....

D. ananassae    CGCGATTTCCTAACGACCCAGCCGCCGCCATCG---------ACGCCGCCGTTTCCCAGCTCTCCCAGCTCAGCGGCT
D. melanogaster .....C..T.GC.GC.TTG.CTTT.....GA..ACCGCGTGT.......G.C.A...TCG---.....T..G..A..G
D. sechellia    .....C..T.GC.GC.TTG.CTTC.....GA..ACTACGGGT.........C.A...TCG---..G.....G..A..G
D. simulans     .....C..T.GC.GC.TTG.CTTC.....GA..ACTACGGGT.........C.A...TCG---..G.....G..A..G
D. yakuba       ........T.GC.GC.TTG.CTTC...G.GA..ACTACGTGT.........C.....TCG---........G..A..G
D. erecta       .....C..T.GC.GC.TTG.CTTT...G.GA..ACTACGTGT.....T...C.....TCG---........G..A..G

D. ananassae    TCTCCG------------GGTCGCAAGAGCTTCACCTCGCTGAACCTCACCCTCCGCCAGCCGGCAACGGGCGGCGGA
D. melanogaster ..A..AAGTGCGCCTGGT..A....................C..T........G.........A.G-------..TCG
D. sechellia    ..A..AAGTGCCCCCGGT..A....................C..T........G.........A.G-------..TCG
D. simulans     ..A..AAGTGCCCCCGGT..A....................C..T........G.........A.G-------.ATCG
D. yakuba       ..G..AAGTGCCCCTGGC..A....................C..T........G.....A...A.G-------..TCG
D. erecta       ..A..AAGTGCCCCTGGC..A....................C..T........G.........A.G-------..TC.

D. ananassae    GCACAGGCGGCGATCGACATTACCGCCGGACCAGCGCCCAGTGGCCAGGGCAGCGGGATCACCTACTCGAGTGTCAGC
D. melanogaster .....AT....C..T.....C........T..G...........G........T..............C..C..T..T
D. sechellia    ..T..AT....C..T.....C........T..G.....T.....G........T..............C..C..T..T
D. simulans     ..G..AT....C..T.....C........T..G...........G........T..............C..C..T..T
D. yakuba       ..G..AT....C..T.....C........T..G...........G........T..............C..C..T..T
D. erecta       ..G..AT....C..T.....C.....T..C..G...........G........T..............C..C..T..T

D. ananassae    TTTGATGCGAGGAAGGGCACGCACAAGAACTTCCAGCTGACGGTCACGGACGAGGGCAGTGTCTTTAGTGCGGGTTGT
D. melanogaster .....C..AC.TCGT..G.....G.....T...........T..G....................C........CA.C
D. sechellia    .....C..AC.CCGT..G.....G.....T...........T..G.................A..C........CA.C
D. simulans     .....C..AC.CCGT..G.....G.....T...........T..A....................C........CA.C
D. yakuba       .....C...C.CCGT..A.....G.....T...........T..G....................C........CA.C
D. erecta       .....C...C.TCGT..A.....G.....T...........T..G....................C.....A..CA.C

D. ananassae    ATACGACCGCGAGTTCCGTGCTCGGGC---TGTGATCCAGCTGCAGTTGCTCCTCCGACGAGTCACCACCAGCCCCAG
D. melanogaster G.T..C..AA.GACCTT..A.G.AACGCCC.....G..G.TGA..AG..T...G------GCAA....G.....T---
D. sechellia    G.T..C..AA..ACCTT..A...AACGCCC.....G..G.TG...AG..T...G------GCAA....G.....T---
D. simulans     G.T..C..AA..ACATT..A...AACGCCC.....G..G.TG...AG..T...G------GCAA....G.....T---
D. yakuba       G.T..T..AA.GAC.TTA.A.G.C.CGCCC.....G..G.TGT..AG..T...A------GCAA.T..A.....G---
D. erecta       G.T..C..AA.GAC.TTA.A.G.A.CGCCT.....G..G.TG...AG..T...A------GCAA.T..A.....T---

D. ananassae    CTGCCACTGTCGCTGCCTCCGCCGCCAGCGGTGGTCGCCGATGGTGGAGTGGGCCAGGAGCAGGGAGGAGCA---ATG
D. melanogaster ---------------........CG....A.....G..A......---------------.T...G.AC..TGAT...
D. sechellia    ---------------........AG....A.....G..A......---------------.T...G.AC..TGAT...
D. simulans     ---------------........AG....A.....G..A......---------------.G...G.AC..TGAT...
D. yakuba       ---------------........AG..T.A.....G..A......---------------.T...G.AC..CGAT...
D. erecta       ---------------..G.....AG....A.....G..G......---------------.T...G.AC..CGAT...

D. ananassae    ACGCCGGACGTGTTCCCATATCCCACGCAGGAGCAGAACCATATCGTGGCATCCAACTACAGTAACAACCATGCGGTG
D. melanogaster ...T.......T..........................T..C..T..............T..............C..C
D. sechellia    ...T.......T..T.......................T..C..T..............T..............C..C
D. simulans     ...T.......T..........................T..C..T..............T..............C..C
D. yakuba       ...T.......T...........A.................C..T..............T........T.....C..C
D. erecta       ...T....T..T.....C.....A.................C..T.....G........T..............C..C

D. ananassae    AACAACAATGGTGGCAATAGCGCCACCAGCAGTCCCGTCAGCAGTCCGACAGTGCCGCTCTACGCCGGAGTGCTGGAG
D. melanogaster .G.G.....AACAA..T.G.GAT..G..AT..CA.G.A...T.....C..CA....C..T....AA..T..CA.....
D. sechellia    .G.G.....AACAA..T.G.GAT.GG..A...CA.G.A...T.....C..CA....C..T....AA.....CA.....
D. simulans     .G.G.....AACAA..T.G.GAT..G..A...CA.G.A...T.....C..CA....C..T....AA.....CA.....
D. yakuba       .G.G....CAACAA..T.G.GAT..G..A...CA.G.A...T.....C..CA....C.......AA.....CA.....
D. erecta       .G.G.....AACAA..T.G.GAT..G..A...CA.G.A...T.....C..CA....C..T....AA.....CA.....

D. ananassae    ------------GAAGCTCATGCCACCACCATCGAGCGACAGAAGAAGCGGCGGGACAAGCTGGCGAATGCTCTGCGG
D. melanogaster GAATGCGATCGC.......G....G....T........T......C....T..C...........C.....A...A..
D. sechellia    GAATGCGATCGC............G.......T............C.......C...........C.........A..
D. simulans     GAATGCGATCGC............G.......T............C.......C...........C.....A...A..
D. yakuba       GAATGCGATCGA............G.A..T..T.....G..A...C.......C...........C.....G...A..
D. erecta       GAATGCGATCGC....T.......G....A..T.....G......C.......C...........C.....G......

D. ananassae    GACAACAGGAAGCGGCTGCTGGTCCTCGAGCAGGAGATCAACATCCTGACCGAGCCGGTTCCCCTGGGAGAATCTGAA
D. melanogaster .......A......C.....C.....G.......................T........G..GG....G.........
D. sechellia    .......A......A.....C.....G.......................T........G..GG....G.........
D. simulans     .......A............C.....G.......................T........G..GG....G.........
D. yakuba       .......A............C.....G.......................T........G..GG....G...A.....
D. erecta       .......A............C.....G.......................T........G..GG....G.........

D. ananassae    AGACTGGATAAAGATATCAAGAAATTGACTGAGGACTGTCAGCGGCTGCTAAACCTCATAAATGAACCGCAGGCGAAT
D. melanogaster ..........G..........C..C..............A.T........TG..TG.T....................
D. sechellia    ..........G..........C..C..............A.T........TG..TG.T....................
D. simulans     ..........G..........C..C..............A.T........TG..TG.T..........A.........
D. yakuba       ..........G..........C..C.............CA..........TG..TG.T..........A.........
D. erecta       ..........G..........C..C..............A....T.....TG..TG.T..........A.........
```

```
D. ananassae    ---------GGCTCGGGCCCGGCACCCAACTCC---ATGAACCGCCAGCATTCGGCGCCTGCCGGAAATGCTCCTCAA
D. melanogaster ---------...C.T..T.....GG....TC..---.C............CCT.TTA..A...A.C......T.A..G
D. sechellia    ---------...C.TA.T.....G.....TC..ACA.C............CCA.TTA..C...A.C........A..G
D. simulans     ---------...C.TA.T.....G.....TC..ACG.C............CCA.TTA..C...A.C........A..G
D. yakuba       CAGGACCAG...C..---------A.A.GTC.A---GC............CCT.CTA..C...A.C........C..G
D. erecta       ---------...C.T..G.....GG....TC.A---.C............C.T.TTA..C...A.C.T......C..G

D. ananassae    CAGCAT---CAAAATCAACCACAGCCATTCCCACGCCAGCGTCAAGGTGGTCGCTCCCAAGTACCGCCCTCTACACTG
D. melanogaster .....GCCG..GC.A..G..............G..T.........A...C....GT...G.CG......AG.T....A
D. sechellia    .....GCCG..GC....G..............G..T.............C....GT...G.CG......AG.T....A
D. simulans     .....GCCG..GC....G..............G..T.............C....GT...G.CG......AG.T....A
D. yakuba       .....GCCG..GC....G..............G..T.............C....GT...G.CG......AG.T.....
D. erecta       ------------C....G..............G..T...........G.C....GT...G.CG..A...AG.T.T...

D. ananassae    AATTTGCATTCAGTGCCGACGCCAAGCAACTTGCAGCCGAATCAGGACTTCTTGCATCAACACCGCAGTGCTCCCAGC
D. melanogaster CG.C....C..G......G.AG..CC..T..CC......GTG........TGG...G..C...A............C.
D. sechellia    CG.C....C..G......G.AG..CC..T..CC......GTG.........GG...G..C...A..........T.C.
D. simulans     CG.C....C..G......G.AG..CC..T..CC......GTG.........GG...G..C...A............C.
D. yakuba       CG.C....C..G......G.AG..CC..T..CC.....TGTG........TGG...G..C...A............C.
D. erecta       CG.C....C..G......G.AG..CCT.T..CC......GCG.........GG...G..C...A............C.

D. ananassae    TCCGCCTGCCTGACGCCACAG---------CAGCAGCAGCAGTTT---------AATGAGCCACCACCCACGTACGCT
D. melanogaster ..G.....TT.A.....C...ATGCAGAGT..A.....A.T...ACAGCTGCAGC.G.....G.....G........C
D. sechellia    ..G........A..C..C...ATGCAGAGT..A.....A.T...ACAGCTGCAGC.G.....G.....G........C
D. simulans     ..G........A.....C...ATGCAGAGT..A.....A.T...ACAGCTGCAGC.G..A..G.....G........C
D. yakuba       ...........A.....C...ATGCAGAGT..A.....AAT...CCAGCTGCAGC.G.....G.....G........C
D. erecta       ..G......T.A.....C...ATGCAGAGT..A.....AAT...CCAGCTGCAGC.G.....G.....G..A..T..C

D. ananassae    CAATACCATCAGTTTCAACAGTACCTGCAGCAGCAGCGCCAGCAGCAACTCCAACAGTTGCAGCTACAGCAGCAACAG
D. melanogaster ..G...T.......C..G..A..T..............A........GT.A......---...A.G............
D. sechellia    ..G...T.......C..G..A..T..............A........GT.A......---...A.G........G...
D. simulans     ..G...T.......C..G..A..T..............A........GT.A..G...---...A.G........G...
D. yakuba       ..G...T.......C..G.....T..............A........GT.G......---...A.G............
D. erecta       ..G...TG......C..G..A.TT..........G...A........GT.A..G...---...A.G....G...G...

D. ananassae    CAGCAATTA---CAGCAACAACAGTTATTG------------CAACAGCAACAGCAACAGCAGCGCCCTGGCAACGAG
D. melanogaster ....T.CA.---..C..GATG...CAGCAA------------..G....CG....C...A..----------------
D. sechellia    ....T.CA.---.....GATG...CAGCAA------------..C....CG....C......----------------
D. simulans     ....T.CA.---.....GATG...CAGCAA------------..G....CG....C......----------------
D. yakuba       .....GCAGTTG..A..G..G......CAACAGCAGATGCAA..G....TG....C...A..----------------
D. erecta       .....GCAG---.....G..G......CAA------------..G....CG....C...A..----------------

D. ananassae    GAGGAGGACTACCTGACCGATTCCGATGTTGACGAGGACGAAGAGCCACTGGACATGTGGGCCTGCAACTTGTGTACA
D. melanogaster .....A..A.T....T....C........G..T.....G..G...A.G......TC.............A........
D. sechellia    .....A..A.T....T....C........G..T.....G..G...A.G......TC.............A........
D. simulans     .....A..A.T....T....C........G..T.....G..G...A.G......TC.............A........
D. yakuba       .....A..A.T..A.T....C.....C..G..T.....G..G...A.G..C...TC.............A........
D. erecta       .....A..A.T..A.T....C........G..T.....G..G...A.G..C...TC.............A........

D. ananassae    TTCCGCAACCATCCGCAACTAAATATTTGCGAGGCCTGTGAGAACGTTAGGATCCAGCCGGGTATGATACGTATTATC
D. melanogaster ..............A..G...........T.........................................C...G.T
D. sechellia    ..............A..G...........T.........................................C...G.T
D. simulans     ..............A..G...........T.........................................C...G.T
D. yakuba       .............................T.........................................C...G.T
D. erecta       ..............A..............T.........................................C...G.T

D. ananassae    CCCAATGCAAGCGGTGGAGGGTCAACT---GCTGCTGCTGGGTCTGCTGGCACGCCTAATGGCAGTCTCGAACAGCAG
D. melanogaster ...------..T..A..C...G.CG..GCT........C------------.....ACCC.....CA.T..G.....A
D. sechellia    ...------.....AA.C...G.CG..GCT........G------------.....CCCC.....C..T..G.....A
D. simulans     ...------.....AA.C...G.CG..GCT........C------------.....CCCC.....C..T..G.....A
D. yakuba       ...------.....A..C.....CG..---........C------------.....CCCC....CCA.T..G......
D. erecta       ...------.....A..C...G.CG..---..A.....C------------..T..CCCA..G.CC..T..G.....A

D. ananassae    CAGCAGCCC---CAACAGCCGTACGCCCTGCATACG
D. melanogaster ........GTCC..G...........T........A
D. sechellia    ........GCCC..G...........T........A
D. simulans     ........GCCC..G...........T........A
D. yakuba       ........GCCC..G...........T........A
D. erecta       ........GCCC..G...........T........A
```

9.Tak1

```
D. ananassae    ATGGCCACCGCATCGCTGGACGCTCTGCAGGCAACCTACGTTGACTTTAACGAGATAAGGCTCGAAGAGAAAGTCGGT
D. melanogaster ........A..............A.........G....T..G.....C.GT.......CA..AAG............C
D. sechellia    ........A...........A..A.....C...G....T..G.....C.GT...C...CA..AAG............C
D. yakuba       .......................A.....A...G....T..G.....C.GT...C...CA..AAG............C
D. erecta       .......................A.........G.............C.GT...C...CA..AAG............C
D. simulans     ........A...........A..A.....C...G....T..G.....C.GT...C...CA..AAG............C

D. ananassae    CATGGGTCCTACGGAGTCGTCTGCAAGGCCATCTGGAGGGACAAGCTGGTGGCCGTCAAGGAGTTCTTCGCCAGCGCC
D. melanogaster .................G............G.T...C.C...........T...........................
D. sechellia    .................G............G.T...C.C...........T...........................
D. yakuba       .................G............G.....C.C....G..................................
D. erecta       .................G............G.....C.C..T....................................
D. simulans     .................G............G.T...C.C.......................................

D. ananassae    GAGCAGAAGGACATCGAAAAGGAGGTGAAGCAGCTGTCGCGGGTCAAGCACGTGAACATCATCGCCCTGCACGGTATC
D. melanogaster .................G...............T.......C..G......CC............T........G..A
D. sechellia    .................G.......................C..G......CC............T........C..A
D. yakuba       .................G...........A...........C..A......CC.........A..T........A..A
D. erecta       .................G.......................C..G.....TCC............T........C..A
D. simulans     .................G.......................C..G......CC.....................C..A

D. ananassae    TCCTCGTTCCAGCAGTCCACCTATTTGATTATGGAGTACGCCGAAGGCGGATCACTGCACAATTTCCTGCACGGGAAG
D. melanogaster .......A.......G.......CC....A.......T.........T.....G........C.....T.....C...
D. sechellia    .......A.......G.......CC....A.......T.........T.....G........C.....T.....C...
```

```
D. yakuba       .......A.......G........CC....C.................T.....C.........C..T..T.....C...
D. erecta       .......A.......G........CC....A.................T.....C.........C.....T.....C...
D. simulans     .......A.......G........CC....A.......T.........T.....G.........C.....T.....C...

D. ananassae    GTGAAGCCGGCATACTCGCTGGCGCATGCCATGAGCTGGGCGCGTCAGTGTGCCGAGGGACTGGCCTATTTGCATGCC
D. melanogaster ..............T..T.....C..C..................C..A.....A.....T.....A............
D. sechellia    ..............T..T..A..C..C..................C..A.....A.....T..T..A............
D. yakuba       .....A.....C..T..C.....C..C..................C........G.....C.....A............
D. erecta       .....A.....C..T..C.....C..C..................C........G.....T.....A..C.........
D. simulans     ..............T..T..A..C..C..................C........A.....T.....A............

D. ananassae    ATGACACCGAAACCCCTGATCCATCGCGATGTCAAGCCACTGAACCTTCTGCTCACCAATAAGGGACGGAACCTGAAG
D. melanogaster .....G..A.....A..A..A........C..G.....G........G..CT.G.....C........C..T......
D. sechellia    .....G..A...........A........C..G.....G........G..CT.G.....C........C..T......
D. yakuba       .....G.....G..G..............C..G.....G........G..C..G.....C.....G..C..T......
D. erecta       .....G.......................C..G.....G........G..CT.G.....C.....G..CG.T......
D. simulans     .....G..A...........A........C..G.....G........G..C..G.....C........C..T......

D. ananassae    ATCTGTGACTTTGGCACCGTCGCCGACAAGTCCACCATGATGACCAACAATCGTGGAAGTGCTGCCTGGATGGCACCA
D. melanogaster ..A..C.....C.....G..G..G........G...................C..C.....C..T........G..C
D. sechellia    ..A..C.....C.....G..G..G........G..............T....C..C.....C..T........G..C
D. yakuba       .....C.....C.....G..G..G........G...................C..C.....C..T........G..C
D. erecta       .....C.....C.....G..G..G........G...........G.......C..C.....C..T........G..C
D. simulans     ..A..C.....C.....G..G..G........G...................C..C.....C..T........G..C

D. ananassae    GAGGTCTTTGAAGGCTCGAAGTACACGGAGAAGTGCGACATTTTCAGCTGGGCGATTGTACTTTGGGAGGTTCTGTCC
D. melanogaster ........C........C.....T...........T........T........C.....T..A...............
D. sechellia    ........C........C.....T...........T.................C.....G..A........G......
D. yakuba       ........C........C.....T........A..T.................C.....G..G........G......
D. erecta       ........C..G.....C..............A..T.................C.....G..A........G......
D. simulans     ........C........C.....T...........T.................C.....G..A........G......

D. ananassae    CGGAAGCAGCCCTTCAAGGGCATCGACAATGCCTACACCATTCAGTGGAAAATCTACAAGGGCGAACGGCCTCCCTTG
D. melanogaster A.............T..A.......................C........G...........T.....C..G..GC..
D. sechellia    A.............T..A.......................C........G...........T.....C..G..GC..
D. yakuba       A.......A.....T..........................C........G...........T.....C..A..GC..
D. erecta       A.......A.....T..........................C........G...........T.....C..G..GC..
D. simulans     A.............T..A.......................C........G...........T.....C..G..GC..

D. ananassae    CTGACCACCTGCCCCAAGCGCATCGAGAACCTGATGACAGCCTGTTGGAAGACAGCGCCCGAGGACCGGCCTTCTATG
D. melanogaster ........T...................G..........C.....C.....A..G.T.........T..C..G..G...
D. sechellia    ............................G..........C.....C.....A..G.T.........T..C..G..G...
D. yakuba       .....A.............G......G..........C.....C.....A..G.T.........T..C..G..G...
D. erecta       .....A.....................G..........C.....C.....A..G.T.........T..C..C..G...
D. simulans     ............................G..........C.....C.....A..G.T.........T..C..G..G...

D. ananassae    CAGTACATCGTGGGCGTCATGCACGAGATCGTGAAGGACTATACGGGGGCGGAGAAGCCCTTGGAGTACACGTTTGTT
D. melanogaster ........A........T.............C....................C...G..C....A............
D. sechellia    ........A......................C....................C...G..C....A............
D. yakuba       ........A......................C....................C...G..C....A.....A......
D. erecta       ........A......................C....................C...G..C..........T......
D. simulans     ........A......................C....................C...G..C....A............

D. ananassae    AATCAACAGATTGTCACCAAAGAGAGCGACGGCACGGTGGCCGCCCAGCCGGACAGCCTCAGTTCGCTGGAGGAGGAC
D. melanogaster .............................................T..A.....T.............A...------
D. sechellia    .............................................T..A.....T.....T.......A...------
D. yakuba       .............................................T..A.....T.............A...------
D. erecta       .....G.......................................T..A.....T.............A...------
D. simulans     .............................................T..A.....T.............A...------

D. ananassae    GACGAACTGAGTCTGTCCACCACACAGTTAACACCCACGTCGGCGGCTAACGCCAATGTGAACGCGAAAGCAATAGCA
D. melanogaster .GG........C.CC..GT................G..AA.......C...................T........T..
D. sechellia    .GG........C.CC..GT................G..AA.......C...................T........T..
D. yakuba       .GG........C.CC..GT................G..AA.......C..............T....T........A..
D. erecta       .GG........C.CC..GT................G..AA.......C...................T........A..
D. simulans     .GG........C.CC..GT................G..AA.......C...................T........T..

D. ananassae    GGCATCGGAACTGCAACAACGACGACAACTAGCTCAATGACCGAAAATACCTCATCAACAACATCATCGGACGCCACG
D. melanogaster AAA------------------..A..G..............................---...........AT....
D. sechellia    AAA------------------..A..G..............................---...........AT....
D. yakuba       .AA------------------..A..G..............................---...........AT....
D. erecta       .AA------------------..A.GC..............................---...........AT....
D. simulans     AAA------------------..A..G..............................---...........AT....

D. ananassae    CCGACCAATTCGGGCCACCTTGACAATAATCCACCGCTATTCCAAATGAGCAGCAATCGCTGGGACGCCATTCCCGAG
D. melanogaster .....G..C........A..G...........G---......T.T...GT..C................G.........
D. sechellia    .....G..C..A.....A..G...........G---A.......C...GT...................G.........
D. yakuba       .....G..C........A..............G---........C...GT...................G.........
D. erecta       .....G..C........A..............G---........C...GT...................G.........
D. simulans     .....G..C........AT.G...........G---A.......C...GT...................G.........

D. ananassae    GAGGAGAGCAACGAGAGCCGGAACGATAGCTTCAATCTCACCTCCTCGGCTGAGGCCACCCAGCGGCTGGAGACGATC
D. melanogaster ....................................C........G.............T.....C..C..A......
D. sechellia    ..................T.................C........G....TG........T.....C..C.........
D. yakuba       .....T.............T................C........G.....G........T.....C..C.........
D. erecta       .....T..............................C........T.....G........T.....C..C.........
D. simulans     ..................T.................C........G....TG..............C..C.........

D. ananassae    CGGAACGGTATGATCCGTATGGCACACACACCGCTGCCGGAACTCTCACTCGACGTGATGGAGAATGGCTTCGATTTG
D. melanogaster ........C.......TG.....CTG..AG..CA..GA.C.G...A.C.........GA..C.........T...C..
D. sechellia    ........C.......TG.....CTG..AG..CA..GA.C.G...A.C.........GA..C...C.....T...C..
D. yakuba       ........C.......TG.....CTG..AG..CA..GA.C.G...A.C.........GA..C.............C..
D. erecta       ........G.......TG.....CTG..AG..CA..GA.C.G...A.C.........GA..C.........T...C..
D. simulans     ........C.......TG.....CTG..AG..CA..GA.C.G...A.C.........GA..C...C.....T...C..

D. ananassae    AGTCGCAGCGAAAGCAGCAGCAGCAGTACGCAC--------------GCTAAGAGCGATGGACGCGAAAGACTCACG
```

```
D. melanogaster ....C.....................C...A..---------------..A...........C......C........
D. sechellia    ....A.....................C...A..---------------..A...........C......C........
D. yakuba       ..........................C...A..---------------..A...........C......C........
D. erecta       ..........................C...A..---------------..A..A........C......C........
D. simulans     ....A.....................C...A..GGTGAGTTTGCCATC...---------------------------

D. ananassae    GTGACGGATACCAAGCCGGTCATCATGACCACTACGGATCTGACCAACAACAACAAC---------------------
D. melanogaster ........C...........G..G..---...C.........T...........GG.------------------GGC
D. sechellia    ........C...........G..G..---...C.....................GG.------------------GGC
D. yakuba       ........C..G........G..G..---...C.....C...G..............---------------------
D. erecta       ........C...........G..G..---...C........................AACAGCGGCAGCCACGCC---
D. simulans     ------------------------------------------------------------------------------

D. ananassae    ---AACATTAACAACAATGTTAGCCATTTAAACAACGGGCTTTTGAGCCATGCC------------------GATAAT
D. melanogaster ATCC..GCCC..------------------TCG.....A..GC..........GAATGGTTGGCAAGCAAGA...G.G
D. sechellia    ATCC..GCCC..------------------TCG.....A..GC..........GAATGGTCGGCAAGCGGCG...G.G
D. yakuba       ---C..GCCC..------------------TCG.....A..GC.C..T.....GAAT------------------G.G
D. erecta       ---C..GCCC..------------------GCG.....A..GC..........GAATGGGCGGCAAGCGGCG...G.G
D. simulans     ------------------------------------------------------------------------------

D. ananassae    GTTCTGCACCAGGATCAGCATCAGGATGCGATTATCAGCTCACTGGACGTGTCCGGGTCCGAT------GGCGACGAG
D. melanogaster .AG.....GG..C.AG.....G..C.G.A....G...A...GT.......CGA.---GTG...------CC.......
D. sechellia    .AG.....GG.AC.AG..T..G..C.G.A....G...A...G........CGA.---GTG...------CC.......
D. yakuba       .AG.....GG..C.GG....CG..C.G.A....G...A...G.........GAT---GTG...GTGGATCC.......
D. erecta       .AG.....GG..C.GG.....G..C.G.A...GG...A...G.........GAT---GTG...------CC...G...
D. simulans     ------------------------------------------------------------------------------

D. ananassae    GATGAGAACGATGGGACAGCCCAGTCGCTGGCAGAGATCCTCGATCCAGAGCTACAGCCCGAACCACCGATACCAAAT
D. melanogaster ...........C..C..C.AA.....A.....C.....T..T.....G.....C.....A..G..G........C..C
D. sechellia    ...........C..C..C.AG..A..A.....C.....T..T.....G.....C.....A..G..G........C..C
D. yakuba       ...........C..C..C.AA..A........C.....T........G.....T.....A..G........T..C..C
D. erecta       ...........C..C..C.AG..A........C.....T........G.....G.....A..G..C........C..C
D. simulans     ------------------------------------------------------------------------------

D. ananassae    AATCCCGAGTCGCAGACCATATACAGGGAGCATCGGCACATGGCCAAGGAGTACCTGAGCGTCGACACCAATCTGTAC
D. melanogaster G..G....A......CT...C...C....C..C..A................................G..C..C...
D. sechellia    G..G....A......CT...C...C.......C..A................................G..C..C...
D. yakuba       G..G...........CT...C...C.......C...................................G..C..C..T
D. erecta       G..G....A......CT...C...C.......C..A.............................T..G..C..C...
D. simulans     ------------------------------------------------------------------------------

D. ananassae    TATGCGCAGGACTTTAAGGAGAAGCTCATCGTACAGATGGATCGAGCCGAGCGGGAGCAGAAGCAGGAGCTGCTGCGC
D. melanogaster ..C...........C.....C...........G........C...A.......C..A..............T......
D. sechellia    ..C...........C.....C...........G........C...A.......C..A..............T......
D. yakuba       ..C...........C.....C........T..G...........CA.......C..A.....................
D. erecta       ..............C.....C........T..G..A........CA.......C..A..............A......
D. simulans     ------------------------------------------------------------------------------

D. ananassae    AAGATTAACCATAAGGAGGACCTGCAGAGTCTCTTCAACAACTTGCAACAGCAGTGGCAGAAGCTACCCGCCGCCCAG
D. melanogaster .....G..GG.C.......GT..T........T.A......TC....G.......AC------------..TT.A.GC
D. sechellia    .....G..GG.C.......GT..........T.A......TC....G.......AC------------..TT.A.GC
D. yakuba       .....G..GG.........GT..A........T.A......TC....G.......AC------------..TT...GC
D. erecta       .....G..GG.C.......GT..........T.A......TC....G.......AC------------..TT...GC
D. simulans     ------------------------------------------------------------------------------

D. ananassae    CAACTCCAGACCGGCCATCATTCGCATCTGCACGCTCACGCCCACGCCCAACAAGGACACTCGCTTCCGCCCACCAAT
D. melanogaster .....TGC.G.....---------------------------------------------------------------
D. sechellia    .....TGC.G.....---------------------------------------------------------------
D. yakuba       .G...TGC.G.....---------------------------------------------------------------
D. erecta       .G...TGCC......---------------------------------------------------------------
D. simulans     ------------------------------------------------------------------------------

D. ananassae    CCTCACTCCCATCACTCCCATCCACATCCCCATCAACATCAGCTGCAGCATCCACATCCGCATCCCCACCAGCCACAG
D. melanogaster ---------.....TC.G..A..T.....T...------------------..G.....AA...AG.TT...------
D. sechellia    ---------.....TC.G..C..T..G......------------------..G...........G..T...------
D. yakuba       ---------.....TC.G.....G.....G..C------------------..T.....T.....G..T...------
D. erecta       ---------.....TC.G..C........G...------------------------------------...------
D. simulans     ------------------------------------------------------------------------------

D. ananassae    CCAACGCACTTGCATCCACATCCACATCCGCATTCGCATCCACACCAGCTGGAG---GAG------------------
D. melanogaster ------..TCCA..CT.......G.C..AT...------TTC-------..C..---..TGAGGGCTGTGGACTGCTG
D. sechellia    ------..TCCA..CT.G.....GA........------TTG.TTGCC...C..---..TGAGGTCTGTGGACTGCTG
D. yakuba       ------..TCCA..CT.G.....C.....C...C....CT.G..TGC..A.C..---...GAGGGCAGTGGACTGCAG
D. erecta       ------..TCC...CT.C...........C...C.....TTG..TGCC.A.C..CAG...GAGGGCTGTGGACTGCTG
D. simulans     ------------------------------------------------------------------------------

D. ananassae    ------AGTGCCGGTGGGGGAGCTGGGATCGGACTAGGAGGCGGGACAACCGATTCGGTGGAGAACGACGGATGGGTG
D. melanogaster CCCGGATCG.TGT.C..A..C---------------------------T.T..G..C.....A---..A..C......
D. sechellia    CACGGGTCG.TGT.C..A..C---------------------------T.A..G..CA....A---..A..C......
D. yakuba       TCCGGATCG.TG..C..A..C---------------------------T...A..C.....A---..A..C......
D. erecta       CCCGGATCG.TG..C..A..C---------------------------T.T..G..C......---..G..C......
D. simulans     ------------------------------------------------------------------------------

D. ananassae    GTGATCCAGCCCCATTCGAATGCG
D. melanogaster ..C....CA..G...CAC......
D. sechellia    ..C....CA..G...CAC..C...
D. yakuba       ..C....CA..G..CCAC..C...
D. erecta       ..C....CAG.G...CAC..C...
D. simulans     ------------------....T.
```

10.Tub

```
D. ananassae    ATGTCCAAGGTAAATGGCTGGAATGGGTGTGGAGCGGGAACGCACGGAAATGGGAGCAGTGCCTCCTTACCACCGACT
D. melanogaster ------.T..CGT..........C..A..C...AT...CGT...G.TG..C..A....AC.G.G..A..GGTTTAT.C
D. sechellia    ------.T..CGT..........C..A..C...AT...CGT...G.TG..C..A....AC.G.G..A..GGCTTAT.C
D. simulans     ------.T..CGT..........C..A..C...AT...CGT...G.TG..C..A....AC.G.G..A..GGCTTAT.C
```

```
D. erecta       ------.T..C.T.........C..A..C...AT...CGT...GATG..C....T..AC.G....A..GGCTTAT.C
D. yakuba       ------.T..CGT.........C..A..C...AT..ACGTT..G.TG..C.......AC.G....A..GGTTTAT.C

D. ananassae    CCTAAATACTTGCGCAGCACAGAACTCCGGCGCGTTGAGGACAACGACATTTACCGGCTGTCCAAAATCCTTGACGAA
D. melanogaster T.G..G..T.CT....A...G..G..G..A..............T..............TG.......A..A..T..G
D. sechellia    T.G..G..T.CC....A...G..G..G..A.....C........T..............TG.......A..A.....G
D. simulans     T.G..G..T.CC....A...G..G..G..A.....C........T..............TG.......A..A.....G
D. erecta       T.A..G....CC..........G..G.......C...........T..G.......TG..........A..T..G
D. yakuba       T.A..G....CC........G..G..G..A.....C.......................TG..........A......

D. ananassae    AACGCCTGTTGGCGCAAGCTCATGTCGATAATACCGAAGGGCCTGGATGTACAGACTTCAAGCGCTGCCGGCGGTTTA
D. melanogaster ...T.A..C........AT.G........C.....C......A.......G...G.C.GC....GA.....AT.C..G
D. sechellia    ...T.A..C........A..G........C.....C......A.......G...G.C.GC....GA.....AT.C...
D. simulans     ...T.A..C........A..G........C.....C......A.......G...G.C.GC....GA.....AT.CC..
D. erecta       ...T....C.....T.....G.....C..T.....C......A.......G..AG.C.GC....GA.....AA.C...
D. yakuba       ...T.T..C...........A.....T..C.....C......A.......G...G.C.GT....GA.....AAAC...

D. ananassae    AACTTTCCAAATGCCATAAAGAAAGGATTCAAGTACACAACACAGGACGCACTGCAAATTGACGAGGCGGCTAACAGA
D. melanogaster ..T.....GGCG.AA..C..A..G.....T........TG.G.......TGT.C..G...........T..A...C..
D. sechellia    ..T.....GGCG.AA..C..A..G.....T........TG.G.......TGT.C..G...........T..G...C..
D. simulans     ..T.....GGCG.AA..C..A..G.....T........TG.G.......TGT.C..G...........T..G...C..
D. erecta       ..T.....TGCG.AA..C..A.........T..A.....TG.G......ATGT.C..G...........T..A...C..
D. yakuba       ..T......GCG.AA..CC.A.........T..A..TT.TG.G...A...TTAGC..G...........T..A.G.C..

D. ananassae    CTAGCACCCGACCAGAGCAAGTCCCAGATGATGATCGATGAGTGGAAGACGTCTGGAAAATTGCACGAGCGGCCAACA
D. melanogaster ...C.G..G.....A........G..............C...........C.....C..GC.CA..........C..G
D. sechellia    ...C.G..G.....A.......................C...........C.....C..GC.CA..........C..G
D. simulans     ...C.G..G.....A.......................C...........C.....C..GC.CA..........C..G
D. erecta       ..GC.G..G..............G..A...........C...........C..C..C..GC.CA..........T...
D. yakuba       ..GT.G..A..............G..A...........C........A..C.....C..GC.TA..........C..G

D. ananassae    GTGGGCGTATTGCTCCAGCTCCTGGTTCAGGCGGAATTGTTCAGTGCAGCCGACTTCGTTGCGCTGGACTTCCTTAAC
D. melanogaster ..T..G...........A..T.....G.....A..GC.C........G..A.....T..G..A..A........A..T
D. sechellia    ..T..G..G........A..T.....G.....A..GC.C........G..A.....T..G..A..A........A...
D. simulans     ..T..G-------------------------------------------------------------------------
D. erecta       .....T..G........A..T.....G.....A..GC.C........G..A.....T..G..C..T........G..T
D. yakuba       .....G..G........A..T.....G.....A..GC.C........G..A.....T..G..A..A........A..T

D. ananassae    GAGCCCAAGCCAGATCGCCCCAGCGACGGACCAGCAGCACCTATAAGT------------------------------
D. melanogaster ...T...CC..T.CC..G..TGTT.....T..C.GT..G.TC.....C------------------------------
D. sechellia    ...T...CC..T.C...G...GTT.....T..C.GT..G.TC.....C------------------------------
D. simulans     ------------------------------------------------GGTGTTGCTCCAACTTCTGGTGCAAGCAGA
D. erecta       ...T...CC..T.C...G..GGTT.....T..T.GT..G..C..T..C------------------------------
D. yakuba       ...T...TC..T.T...G..G.TT.....T..C.....G..C..C..C------------------------------

D. ananassae    ---------------------------------------------CTGGACTTAACGGAGCTTCTTGACGAAACCATG
D. melanogaster ---------------------------------------------..T----------.....G..G..A..GGAA...
D. sechellia    ---------------------------------------------..T----------.....G..G..A..GGAA...
D. simulans     GCTCTTCAGTGCGGCAGACTTTGTGGCACTAGACTTCCTAAACGC..T----------.....G..G..A..GGAA...
D. erecta       ---------------------------------------------...----------.....G..G..A..GGAA...
D. yakuba       ---------------------------------------------...----------.....G.CG..T..TGA....

D. ananassae    GATGTTGACGGCGACGGCCTCAAT------------CCGAGTCCTTCAAATGTG------GCTGCCCAGGGAAGTATT
D. melanogaster ..A..G...AA...G..G..G.GCCTCAAATACCAGT.A..CA..G.C.C.C.TGGGGCA.AC..T..A..T..CG..
D. sechellia    ..A..G...AA...G..G..G.GCCTTAAATACCAG..A..CA..G.C.C.C.TGGGGCG..C..T..A..T..CG..
D. simulans     ..A..G...AA...G..G..G.GCCTTAAATACCAG..A..CA..G.C.C.C.TGGGGCG..C..T..A..T..CG..
D. erecta       ..A..G...AA...G..AG.G.GCCTCAAATACCAGT.A..CA..G.CGC.C.TGGGGGA.GC..T..A..T..CG..
D. yakuba       ..A..G...AA...T..G..G..CCTCAGATACCAGTTA..CA..G.CTC...TGGGGGA.GC..T..A..T..CG..

D. ananassae    GGCCTTAATTTGGACAACTTTGACAAGCAGATTATGCCGCGGAACAAAAGCCTGCCCCAACCGTCTGAGACTGTTCCT
D. melanogaster .....G..CC.............A..AG.C...G..AG....G....G..TG.......G.....A.GA.ACACC...
D. sechellia    .....G..CC.............A..AG.C...G..AG....G....G..TG....G..G.....A.GA.ACACC...
D. simulans     .....C..CC.............A..AG.C...G..AG..A.G....G..TG....G..G.....A.GA.ACACC...
D. erecta       ..A..G..CC......C..A...A..AG.C..GG.AAG.A..G....G..TG.......G.......G.GAC..C...
D. yakuba       .....G..CC......CT.....A..AG.C..GG..AG.A..G....G..TG.A.....GT....A.G..TC.CC.GC

D. ananassae    CCCATAGCGCCGCCCCGTACCTCTCGTCTGCATAGGGACGCAACCAGCTCA---------AACAATGCCACCGTATCG
D. melanogaster ...........A..T..A-------.A.A...A...AGTA.T..T.A...CAACTTTGCA.CA------------.T.
D. sechellia    ...........A..T..A-------.A.A..CA...AGTA.T..T.A...CAACTTTGCC.CA------------.T.
D. simulans     ...........A..T..A-------.A.A..CA...AGT..T..T.A...CAACTTTGCC.CA------------.T.
D. erecta       ........T..A..T..A-------.A.A..CA....GTA.T..A...A.C---------.GT------------.T.
D. yakuba       ...T...T...A..T..A-------.A.A..CAC..A.TAAT..T.....C---------.G.------------.TC

D. ananassae    ACT---GCGACCGGGTCTACGTCGGCGACCACGCCCAATATACCTAATCTAACAATTCTCAACCGCACCGAACAATTA
D. melanogaster ..A---.GC.....AA........A......TC..G...G.G..C.....G...........T.C..G.......A.T
D. sechellia    ..A---.GC......ATC......A......TC..G...G.G..C.....G...........T.C..G.......A.T
D. simulans     ..A---.GC......A.C......A......TC..G...G.G..C.....G...........T.C..G.......A.T
D. erecta       .T.---.G.C.A.TAA.CGT.C.....T...TC..G...G.G..C..C..G...........T.C..G.....C.A.T
D. yakuba       ..CGCA.GC...A.AA.C.T...........TC..A...G.G..C..CT.G........A..T.C..G.......A.T

D. ananassae    GCGGAGCAGGAGAATCAGCCTCGACCGCAAAACATCCCAAATTTGTCCATACTCATAGCAAGCTCAACATCTTCAATG
D. melanogaster CA.....CA.T.CTG.....A......ATG........CG..C....T........TT.G.A...TGGTGA..TGCG.
D. sechellia    CAA....CA.T.CTG.....A.......TG........CG..C....T.....T..T..G.A...TGGTGA..TGGG.
D. simulans     CAA....CA.T.CTG.....A.......TG........CG..C....T........T..G.A...TGGTGA..TGGG.
D. erecta       CA......A.TCCTG...T.A.......TG..T......G..C....T........T........TGGTGA....GG.
D. yakuba       CA......A.TTCTG.....A.....A.CG..T......G..C....T.....TG.T....A...GGGTGA....GG.

D. ananassae    ACAACGCAATCCACTTCTTTGGGGCAGCCAAACAGTCAACAGGATGGCTCTGCGGACACACCAAACATACCTAAAATT
D. melanogaster G....TGTG..GGACAAC------------CCA..CA.CAGAAC.A....AA....TC.G..T.....T..GCG...C
D. sechellia    G....TGTG..GGACAAC------------CCA..CA.CAGAAC.A....AA....TC.G........T..GCG...C
D. simulans     G....TGTG..GGACAAC------------CCA..CA.CAGAAC.A....AA....TC.G........T..GCG...C
D. erecta       GA...ATC...GGACAAC-------------.CA..CA.CA.A.CCA....AAG...T..GA.......T..GCG...C
D. yakuba       G....TTTG..TGGCAAC-------------.CA..CA.CA.ATCCAA...AA.A..TT.GA..........GCG...C

D. ananassae    ACGCTTTTGATTGAGAACTCTGCCCAGATAAGCCAG--------------------------CCAACGACTGAAGCA
D. melanogaster ..T.....A.....T..T....GAG.TG...A.TCTCGACCAAATCACGCTCCAGCGAAAGCTT.....G..ACCA..
```

```
D. sechellia     ..T.....A.....T..T....GAG.TG.....TCTCGACCAAATCACGCTCCAGCGAAAGCT......G..ACCA..
D. simulans      ..T.....A........T....GAG.TG.....TCTCGACCAAATCACGCTCCAGCGAAAGCT......G..ACCA..
D. erecta        ..T.....A.....T..T---------------TCTCGACCAAATCACGCCGCAGCGAAAGCT........GACC---
D. yakuba        ..T.....A.....T..T.....AG.TT.....TCTCAATCAAATCACGATGCAGCGAAAGCT......G..ACCA..

D. ananassae     GCGGAAACGAGCTTAAACAATTTGCCCATGATAAGCGCATTAAATCTGAACACTAGCAACGGAGAGTCCCCGCCCCCT
D. melanogaster  T.AACGG......C......C.....A...........C..G...A.A.GT.AAG...G.A.G...A..TT..GT...
D. sechellia     T.AACGGT.T...CG.....C.....A...........C..G..CA.A.GT.AAGA..G.A.G...A.TTT..GT...
D. simulans      T.AACGGT.T...CG.....C.....A...........C..G..CA.A.GT.AAGA..G.A.G...A.TTT..GT...
D. erecta        ------------CC......C.....A...........C..G...A.A.GT.A.GA..G...G...A.TTTA.GT...
D. yakuba        T.AATTG...CA.C......C.....A...........C..G...A.A..T.A.GA..G...G...A.TTT..GT...

D. ananassae     AACAGT---GACAGTGACAGCAGCCTGAGTAACGACGAAGATGAA---------------------GGAGATGCTGAC
D. melanogaster  G.G..CAGAAG...CTC.......T....C..A..T..T..C..TGATAACGATGGTGAGGAGGAT.....G.AA..G
D. sechellia     G.G..CAGAAG...CTC..T....T....C..A..T..T..C..TGATAACGATGGTGAGGAGGAT.....G.AA..G
D. simulans      G.G..CAGAAG...CTC.......T....C..A..T..T..C..TGATAACGATGGTGAGGAGGAT.....G.AA..G
D. erecta        G.G..CAGAAG....TC.......T....C..A..T..C..C..TGATATCGATGGTGAGGAGGAT...---------
D. yakuba        G.G..CAGAAG....TT.......T.A..C..A..T..T..C..TGATATCGATGGGGATGAGGAT.T...G.AA..A

D. ananassae     GATCCGGATGCATCGCTGCGAAACCTGAGCAACTCGGAGCAACAGAACTCTAACAACGATTCCAGCCTGACAACGGTG
D. melanogaster  T.C..A.....C.TCT...C...T.....T.....A.....G....G...A.....T..C.................T
D. sechellia     T.C..A.....C.TCT...C.........T.....A.....G........A.....T..C.................T
D. simulans      T.C..A.....C.TCT...C.........T.....A.....G........A.....T..C.................T
D. erecta        ------.....C.TC...A........T.T.....A.....G........A.....T..C..T...T..........T
D. yakuba        T.C..A.....C.TC....C.......T.T....T......G........A.....T....................T

D. ananassae     ACTGGCACCAGCGGTGATAACAGCTTCGAAATGACCAACGACTCAAGTTCGACCTCGAACGACGATTATGGCGGAAAT
D. melanogaster  ...........T..............T..GC.A............C..C..C.....A...........C.CTT.C..C
D. sechellia     ..........................T..GC..............C..C..C.....A...........C.CTT.C..C
D. simulans      ..........................T..GC..............C..C..C.....A...........C.CTT.C..C
D. erecta        ..........................T..GC..............C..C..C.....A...........C.CTT.C..C
D. yakuba        ..........................T..GC..............C..C..C.....A...........C.CTT.T..C

D. ananassae     ATTCCAAACCTGAGTGAACTGCAGCCG
D. melanogaster  ......G..T.......G.......AA
D. sechellia     ......G..T.......G.......AA
D. simulans      ......G..T.......G.......AA
D. erecta        ......G..T.......G.......AA
D. yakuba        ......G..T.......G.......AA
```
